# Supplementary material for: Prevalence of dermatological manifestations due to face mask use and its associated factors during COVID-19 among the general population of Bangladesh: A nationwide cross-sectional survey
Source: PLoS One. 2022 Jun 13;17(6):e0269922. doi: 10.1371/journal.pone.0269922 (PMC9191724; doi:10.1371/journal.pone.0269922)
Supplement: S1 File — Complete data set of this study. (PDF) [file pone.0269922.s001.pdf]

occupation	age	humidity	sex	hx_dm	hx_skin	hx_obesity	worktime	mask_use
Emloyed	23	>9 months	Female	No	Yes	No	Not applica	Yes
Emloyed	25	>9 months	Male	No	No	No	24-48 hour	Yes
Emloyed	25	>9 months	Male	No	No	No	24-48 hour	Yes
Emloyed	56	8-9 moths	Male	No	No	No	< 24 hours/	Yes
Emloyed	56	8-9 moths	Male	No	No	No	< 24 hours/	Yes
Unemploye	21	>9 months	Female	No	Yes	No	Not applica	Yes
Unemploye	20	>9 months	Female	No	No	No	Not applica	Yes
Unemploye	63	>9 months	Female	Yes	Yes	No	Not applica	Yes
Unemploye	22	>9 months	Female	No	No	No	Not applica	Yes
Emloyed	40	>9 months	Male	Yes	No	Yes	24-48 hour	Yes
Unemploye	24	>9 months	Male	No	No	No	Not applica	Yes
Unemploye	21	>9 months	Female	No	No	No	Not applica	Yes
Emloyed	56	>9 months	Male	No	Yes	No	24-48 hour	Yes
Unemploye	21	>9 months	Female	No	No	No	Not applica	Yes
Unemploye	20	>9 months	Female	No	No	No	< 24 hours/	Yes
Emloyed	38	>9 months	Female	No	No	No	>48 hours/	Yes
Emloyed	42	>9 months	Male	No	Yes	No	24-48 hour	Yes
Unemploye	21	>9 months	Male	No	No	No	Not applica	Yes
Emloyed	73	>9 months	Male	Yes	Yes	No	>48 hours/	Yes
Emloyed	36	>9 months	Female	No	No	Yes	< 24 hours/	Yes
Unemploye	47	>9 months	Female	No	No	No	Not applica	Yes
Unemploye	17	>9 months	Male	No	No	No	Not applica	Yes
Emloyed	53	>9 months	Male	Yes	No	No	< 24 hours/	Yes
Emloyed	48	>9 months	Female	No	No	No	< 24 hours/	Yes
Unemploye	22	>9 months	Male	No	No	No	Not applica	Yes
Unemploye	42	>9 months	Female	No	No	Yes	Not applica	Yes
Unemploye	20	>9 months	Female	No	No	Yes	Not applica	Yes
Unemploye	20	>9 months	Female	No	No	No	Not applica	Yes
Unemploye	17	>9 months	Female	No	Yes	No	Not applica	Yes
Unemploye	16	>9 months	Male	No	No	No	Not applica	Yes
Emloyed	35	>9 months	Female	No	No	Yes	< 24 hours/	Yes
Unemploye	55	>9 months	Female	Yes	No	Yes	Not applica	Yes
Unemploye	21	>9 months	Female	No	No	No	Not applica	Yes
Unemploye	20	>9 months	Female	No	No	No	Not applica	Yes
Unemploye	23	>9 months	Male	No	No	No	Not applica	Yes
Unemploye	21	>9 months	Female	No	No	No	Not applica	Yes
Unemploye	20	>9 months	Female	No	No	No	Not applica	Yes
Unemploye	22	>9 months	Male	No	No	No	Not applica	Yes
Unemploye	20	>9 months	Female	No	No	No	Not applica	Yes
Unemploye	18	>9 months	Male	No	No	No	Not applica	Yes
Unemploye	21	>9 months	Male	No	No	No	Not applica	Yes
Unemploye	21	>9 months	Female	No	No	No	24-48 hour	Yes
Unemploye	21	>9 months	Female	No	No	No	Not applica	Yes
Unemploye	18	>9 months	Male	No	No	No	Not applica	Yes
Unemploye	20	>9 months	Male	No	No	No	Not applica	Yes
Emloyed	26	>9 months	Male	No	No	No	< 24 hours/	Yes
Unemploye	21	>9 months	Female	No	No	Yes	Not applica	Yes
Unemploye	50	>9 months	Female	Yes	No	No	Not applica	Yes
Unemploye	21	>9 months	Female	No	No	No	< 24 hours/	Yes
Emloyed	58	>9 months	Male	Yes	No	No	< 24 hours/	Yes
Unemploye	21	>9 months	Female	No	No	No	Not applica	Yes

Unemploye	20	>9 months	Female	No	No	No	Not applica	Yes
Unemploye	20	>9 months	Female	No	No	Yes	Not applica	Yes
Emloyed	30	>9 months	Female	No	No	No	>48 hours/\	Yes
Unemploye	21	>9 months	Male	No	No	No	Not applica	Yes
Unemploye	20	>9 months	Female	No	No	Yes	< 24 hours/	Yes
Unemploye	21	>9 months	Female	No	No	No	Not applica	Yes
Unemploye	17	>9 months	Male	No	No	No	Not applica	Yes
Unemploye	20	>9 months	Female	No	No	Yes	Not applica	Yes
Unemploye	18	>9 months	Male	No	No	No	Not applica	Yes
Unemploye	17	>9 months	Male	No	No	No	Not applica	Yes
Unemploye	18	>9 months	Female	No	No	No	Not applica	Yes
Unemploye	20	>9 months	Female	No	No	No	Not applica	Yes
Unemploye	21	>9 months	Male	No	No	No	< 24 hours/	Yes
Unemploye	17	>9 months	Female	No	No	No	Not applica	Yes
Unemploye	17	>9 months	Male	No	No	No	Not applica	Yes
Unemploye	21	>9 months	Female	No	No	No	Not applica	Yes
Unemploye	15	>9 months	Female	No	No	No	Not applica	Yes
Unemploye	19	>9 months	Female	No	No	Yes	Not applica	Yes
Unemploye	19	>9 months	Male	No	No	No	Not applica	Yes
Emloyed	46	>9 months	Female	No	No	No	>48 hours/\	Yes
Unemploye	20	>9 months	Female	No	No	No	Not applica	Yes
Unemploye	24	>9 months	Male	No	No	No	Not applica	Yes
Unemploye	20	8-9 moths	Female	No	No	Yes	Not applica	Yes
Emloyed	54	>9 months	Male	Yes	No	No	< 24 hours/	Yes
Unemploye	17	>9 months	Male	No	No	No	Not applica	Yes
Unemploye	23	>9 months	Male	No	Yes	No	< 24 hours/	Yes
Unemploye	19	>9 months	Female	No	No	No	Not applica	Yes
Emloyed	33	>9 months	Male	No	No	Yes	>48 hours/\	Yes
Unemploye	24	>9 months	Male	No	No	Yes	Not applica	Yes
Unemploye	20	8-9 moths	Female	No	No	No	Not applica	Yes
Unemploye	20	>9 months	Male	No	No	No	Not applica	Yes
Unemploye	19	>9 months	Female	No	No	Yes	Not applica	Yes
Unemploye	24	8-9 moths	Female	No	No	No	Not applica	Yes
Unemploye	20	>9 months	Male	No	No	No	Not applica	Yes
Unemploye	25	>9 months	Male	No	Yes	No	Not applica	Yes
Unemploye	30	8-9 moths	Female	No	Yes	Yes	Not applica	Yes
Emloyed	38	8-9 moths	Male	No	No	No	>48 hours/\	Yes
Unemploye	20	8-9 moths	Female	No	No	No	Not applica	Yes
Unemploye	21	8-9 moths	Male	No	No	No	Not applica	Yes
Unemploye	20	8-9 moths	Male	No	No	No	Not applica	Yes
Unemploye	21	8-9 moths	Male	No	Yes	No	< 24 hours/	Yes
Unemploye	20	8-9 moths	Male	No	No	No	Not applica	Yes
Unemploye	21	< 8 months	Male	No	No	No	< 24 hours/	Yes
Unemploye	20	8-9 moths	Male	No	No	No	Not applica	Yes
Unemploye	20	>9 months	Male	No	No	No	Not applica	Yes
Unemploye	21	8-9 moths	Male	No	Yes	No	< 24 hours/	Yes
Unemploye	20	>9 months	Female	No	Yes	Yes	< 24 hours/	Yes
Unemploye	19	8-9 moths	Male	No	No	Yes	Not applica	Yes
Unemploye	20	8-9 moths	Male	No	No	No	Not applica	Yes
Unemploye	21	8-9 moths	Male	No	No	Yes	Not applica	Yes
Unemploye	21	8-9 moths	Male	No	No	Yes	Not applica	Yes
Unemploye	23	>9 months	Female	No	No	No	Not applica	Yes

Unemploye	18	>9 months	Female	No	Yes	No	Not applica	Yes
Unemploye	20	>9 months	Female	No	No	No	Not applica	Yes
Unemploye	57	>9 months	Female	Yes	No	No	Not applica	Yes
Unemploye	21	8-9 moths	Female	No	No	No	< 24 hours/	Yes
Unemploye	18	>9 months	Female	No	Yes	No	< 24 hours/	Yes
Unemploye	18	>9 months	Female	No	Yes	No	< 24 hours/	Yes
Unemploye	20	>9 months	Female	No	No	No	Not applica	Yes
Unemploye	21	>9 months	Female	No	Yes	No	< 24 hours/	Yes
Unemploye	21	>9 months	Female	No	No	Yes	Not applica	Yes
Unemploye	21	8-9 moths	Male	No	No	No	< 24 hours/	Yes
Unemploye	19	< 8 months	Male	No	No	Yes	Not applica	Yes
Unemploye	31	>9 months	Male	No	No	No	24-48 hour	Yes
Emloyed	25	>9 months	Male	Yes	No	Yes	< 24 hours/	Yes
Emloyed	39	>9 months	Male	No	No	No	>48 hours/	Yes
Unemploye	20	>9 months	Female	No	No	Yes	Not applica	Yes
Unemploye	22	< 8 months	Male	No	No	No	Not applica	Yes
Unemploye	24	>9 months	Male	No	No	No	Not applica	Yes
Unemploye	18	>9 months	Female	No	Yes	No	Not applica	Yes
Unemploye	20	8-9 moths	Female	No	No	No	Not applica	Yes
Unemploye	21	>9 months	Female	No	No	No	Not applica	Yes
Unemploye	22	< 8 months	Male	No	No	No	Not applica	Yes
Unemploye	20	>9 months	Male	No	No	Yes	< 24 hours/	Yes
Emloyed	30	>9 months	Male	No	No	Yes	< 24 hours/	Yes
Unemploye	20	>9 months	Male	No	No	No	Not applica	Yes
Emloyed	55	>9 months	Male	No	No	No	24-48 hour	Yes
Unemploye	25	>9 months	Female	No	No	No	Not applica	Yes
Unemploye	20	8-9 moths	Male	No	No	No	Not applica	Yes
Unemploye	19	>9 months	Female	No	No	No	Not applica	Yes
Unemploye	20	8-9 moths	Female	No	Yes	Yes	Not applica	Yes
Unemploye	20	>9 months	Female	No	Yes	Yes	Not applica	Yes
Unemploye	21	8-9 moths	Male	No	No	No	Not applica	Yes
Unemploye	20	>9 months	Female	No	No	No	Not applica	Yes
Unemploye	22	>9 months	Male	No	No	No	< 24 hours/	Yes
Unemploye	21	>9 months	Male	No	No	No	Not applica	Yes
Unemploye	21	>9 months	Female	No	No	No	Not applica	Yes
Unemploye	20	8-9 moths	Female	No	No	No	Not applica	Yes
Unemploye	20	>9 months	Male	No	No	No	Not applica	Yes
Unemploye	19	>9 months	Female	No	No	No	< 24 hours/	Yes
Unemploye	21	>9 months	Female	No	No	No	Not applica	Yes
Unemploye	21	>9 months	Male	No	No	No	Not applica	Yes
Unemploye	21	>9 months	Female	No	No	No	Not applica	Yes
Unemploye	21	>9 months	Male	No	Yes	No	Not applica	Yes
Unemploye	21	>9 months	Female	No	No	No	Not applica	Yes
Unemploye	21	>9 months	Female	No	No	No	< 24 hours/	Yes
Emloyed	50	>9 months	Male	Yes	No	Yes	< 24 hours/	Yes
Unemploye	46	>9 months	Female	Yes	Yes	No	Not applica	Yes
Unemploye	21	>9 months	Female	No	No	No	Not applica	Yes
Unemploye	21	>9 months	Female	No	No	No	Not applica	Yes
Unemploye	67	>9 months	Female	Yes	No	No	Not applica	Yes
Unemploye	20	>9 months	Female	No	No	No	24-48 hour	Yes
Unemploye	21	>9 months	Male	No	No	Yes	Not applica	Yes
Unemploye	20	>9 months	Female	No	No	No	Not applica	Yes

Emloyed	24	>9 months	Male	No	Yes	No	>48 hours/\	Yes
Unemploye	20	>9 months	Female	No	No	No	Not applica	Yes
Unemploye	23	>9 months	Female	No	No	No	Not applica	Yes
Unemploye	25	>9 months	Male	No	No	No	Not applica	Yes
Unemploye	20	>9 months	Male	No	No	No	Not applica	Yes
Unemploye	19	8-9 moths	Male	No	No	No	Not applica	Yes
Unemploye	20	>9 months	Male	No	No	No	< 24 hours/	Yes
Unemploye	20	8-9 moths	Male	No	No	No	Not applica	Yes
Unemploye	21	>9 months	Female	No	Yes	No	< 24 hours/	Yes
Unemploye	20	>9 months	Male	No	No	No	Not applica	Yes
Unemploye	17	>9 months	Female	No	Yes	Yes	Not applica	Yes
Unemploye	21	>9 months	Female	No	No	No	Not applica	Yes
Unemploye	20	>9 months	Female	No	No	No	Not applica	Yes
Unemploye	21	>9 months	Female	No	No	No	< 24 hours/	Yes
Unemploye	21	>9 months	Female	No	No	No	Not applica	Yes
Unemploye	20	>9 months	Male	No	No	No	Not applica	Yes
Unemploye	21	>9 months	Female	No	No	No	24-48 hour	Yes
Unemploye	21	8-9 moths	Male	No	Yes	Yes	Not applica	Yes
Unemploye	21	>9 months	Male	No	No	No	Not applica	Yes
Unemploye	21	>9 months	Female	No	No	No	Not applica	Yes
Unemploye	20	>9 months	Male	No	Yes	No	Not applica	Yes
Unemploye	18	>9 months	Female	No	No	No	Not applica	Yes
Unemploye	20	>9 months	Female	No	Yes	No	Not applica	Yes
Unemploye	22	>9 months	Female	No	No	No	Not applica	Yes
Unemploye	19	>9 months	Male	No	No	No	Not applica	Yes
Unemploye	21	>9 months	Male	No	No	No	Not applica	Yes
Unemploye	21	>9 months	Male	No	No	No	Not applica	Yes
Unemploye	21	8-9 moths	Female	No	No	Yes	24-48 hour	Yes
Unemploye	24	>9 months	Female	No	No	No	Not applica	Yes
Unemploye	20	< 8 months	Female	No	No	No	< 24 hours/	Yes
Unemploye	15	>9 months	Female	No	No	No	Not applica	Yes
Unemploye	20	8-9 moths	Female	No	No	Yes	< 24 hours/	Yes
Unemploye	21	>9 months	Female	No	No	No	Not applica	Yes
Unemploye	21	>9 months	Female	No	Yes	No	Not applica	Yes
Unemploye	19	8-9 moths	Male	No	Yes	Yes	24-48 hour	Yes
Unemploye	20	< 8 months	Female	No	No	No	Not applica	Yes
Unemploye	20	>9 months	Male	No	No	No	Not applica	Yes
Unemploye	21	>9 months	Male	No	No	No	Not applica	Yes
Unemploye	20	8-9 moths	Female	No	No	No	< 24 hours/	Yes
Unemploye	20	>9 months	Female	No	No	No	Not applica	Yes
Unemploye	20	>9 months	Female	No	No	No	Not applica	Yes
Unemploye	21	>9 months	Female	No	Yes	No	Not applica	Yes
Unemploye	21	8-9 moths	Female	No	No	No	Not applica	Yes
Unemploye	20	>9 months	Female	No	No	No	< 24 hours/	Yes
Emloyed	31	>9 months	Female	No	Yes	No	< 24 hours/	Yes
Unemploye	19	>9 months	Female	No	No	No	Not applica	Yes
Emloyed	54	>9 months	Male	No	No	No	< 24 hours/	Yes
Unemploye	20	>9 months	Male	No	No	Yes	Not applica	Yes
Unemploye	23	>9 months	Female	No	No	No	Not applica	Yes
Unemploye	20	>9 months	Female	No	No	No	Not applica	Yes
Unemploye	21	>9 months	Female	No	No	No	Not applica	Yes
Unemploye	20	>9 months	Female	No	No	No	Not applica	Yes

Unemploye	20	>9 months	Female	No	Yes	No	Not applica	Yes
Unemploye	18	>9 months	Female	No	No	No	Not applica	Yes
Emloyed	46	>9 months	Female	Yes	No	No	>48 hours/\	Yes
Emloyed	56	>9 months	Male	No	No	No	>48 hours/\	Yes
Emloyed	38	>9 months	Male	Yes	No	Yes	>48 hours/\	Yes
Emloyed	29	>9 months	Female	No	Yes	No	24-48 hour	Yes
Unemploye	22	8-9 moths	Male	No	No	Yes	Not applica	Yes
Unemploye	20	>9 months	Female	No	Yes	No	Not applica	Yes
Unemploye	21	>9 months	Female	No	No	No	Not applica	Yes
Emloyed	39	>9 months	Male	No	No	No	24-48 hour	Yes
Unemploye	20	>9 months	Female	No	No	Yes	Not applica	Yes
Unemploye	32	>9 months	Female	No	No	No	Not applica	Yes
Unemploye	24	>9 months	Female	No	No	No	Not applica	Yes
Unemploye	21	>9 months	Female	No	No	No	Not applica	Yes
Unemploye	21	>9 months	Female	No	No	No	Not applica	Yes
Unemploye	21	>9 months	Female	No	Yes	No	Not applica	Yes
Emloyed	25	>9 months	Female	No	No	No	24-48 hour	Yes
Unemploye	21	>9 months	Male	No	No	No	Not applica	Yes
Unemploye	21	>9 months	Female	No	No	No	< 24 hours/	Yes
Unemploye	21	>9 months	Female	No	No	No	24-48 hour	Yes
Unemploye	21	>9 months	Female	No	No	No	Not applica	Yes
Unemploye	21	>9 months	Female	No	No	No	Not applica	Yes
Unemploye	20	>9 months	Female	No	Yes	No	Not applica	Yes
Unemploye	21	>9 months	Female	No	No	No	Not applica	Yes
Emloyed	29	>9 months	Female	No	No	No	24-48 hour	Yes
Unemploye	37	>9 months	Female	Yes	No	No	Not applica	Yes
Emloyed	41	>9 months	Female	No	No	No	>48 hours/\	Yes
Emloyed	46	>9 months	Female	No	No	No	< 24 hours/	Yes
Emloyed	33	>9 months	Male	No	No	No	24-48 hour	Yes
Unemploye	22	>9 months	Male	No	Yes	No	Not applica	Yes
Unemploye	22	>9 months	Male	No	No	No	Not applica	Yes
Unemploye	21	>9 months	Male	No	No	No	Not applica	Yes
Unemploye	22	>9 months	Female	No	No	No	Not applica	Yes
Unemploye	21	>9 months	Male	No	No	No	< 24 hours/	Yes
Unemploye	20	8-9 moths	Male	No	No	No	Not applica	Yes
Unemploye	20	>9 months	Female	No	No	No	Not applica	Yes
Unemploye	23	8-9 moths	Female	No	No	No	Not applica	Yes
Unemploye	21	>9 months	Female	No	No	No	Not applica	Yes
Unemploye	26	>9 months	Female	No	Yes	No	Not applica	Yes
Unemploye	21	8-9 moths	Male	No	No	No	< 24 hours/	Yes
Unemploye	18	8-9 moths	Male	No	No	No	Not applica	Yes
Unemploye	19	>9 months	Male	No	No	No	Not applica	Yes
Unemploye	19	8-9 moths	Male	No	No	No	Not applica	Yes
Unemploye	20	>9 months	Male	No	No	No	Not applica	Yes
Unemploye	21	8-9 moths	Female	No	No	No	Not applica	Yes
Unemploye	20	>9 months	Female	No	No	No	< 24 hours/	Yes
Unemploye	20	8-9 moths	Female	No	No	No	Not applica	Yes
Emloyed	46	>9 months	Female	No	No	No	24-48 hour	Yes
Unemploye	20	8-9 moths	Male	No	No	No	Not applica	Yes
Unemploye	21	>9 months	Female	No	No	No	Not applica	Yes
Unemploye	21	8-9 moths	Male	No	No	No	Not applica	Yes
Unemploye	22	>9 months	Female	No	No	No	Not applica	Yes

Unemploye	22	>9 months	Male	No	No	No	Not applica	Yes
Unemploye	21	>9 months	Female	No	No	No	< 24 hours/	Yes
Unemploye	20	8-9 moths	Female	No	No	No	Not applica	Yes
Unemploye	20	>9 months	Female	No	No	No	Not applica	Yes
Unemploye	21	>9 months	Female	No	No	No	Not applica	Yes
Unemploye	20	< 8 months	Male	No	No	No	Not applica	Yes
Unemploye	48	8-9 moths	Female	Yes	No	No	Not applica	Yes
Emloyed	35	>9 months	Male	Yes	No	No	24-48 hour	Yes
Emloyed	54	8-9 moths	Male	No	No	No	< 24 hours/	Yes
Unemploye	20	8-9 moths	Female	No	No	No	Not applica	Yes
Unemploye	21	>9 months	Female	No	Yes	No	Not applica	Yes
Unemploye	22	>9 months	Female	No	No	No	Not applica	Yes
Emloyed	45	8-9 moths	Male	Yes	No	No	>48 hours/	Yes
Unemploye	48	8-9 moths	Female	Yes	No	No	Not applica	Yes
Emloyed	56	8-9 moths	Male	No	Yes	No	24-48 hour	Yes
Unemploye	59	8-9 moths	Male	Yes	No	No	Not applica	Yes
Unemploye	66	8-9 moths	Female	Yes	No	No	Not applica	Yes
Unemploye	48	>9 months	Female	Yes	Yes	Yes	Not applica	Yes
Unemploye	61	>9 months	Male	Yes	Yes	No	Not applica	Yes
Unemploye	23	8-9 moths	Male	No	Yes	Yes	Not applica	Yes
Emloyed	24	>9 months	Male	No	No	No	24-48 hour	Yes
Unemploye	22	>9 months	Male	No	No	No	Not applica	Yes
Unemploye	23	>9 months	Male	No	No	No	24-48 hour	Yes
Unemploye	20	>9 months	Male	No	Yes	No	Not applica	Yes
Unemploye	20	>9 months	Male	No	No	No	Not applica	Yes
Unemploye	14	>9 months	Female	No	No	No	Not applica	Yes
Unemploye	13	>9 months	Male	No	No	No	Not applica	Yes
Unemploye	13	>9 months	Female	No	No	No	Not applica	Yes
Unemploye	23	>9 months	Male	No	No	No	Not applica	Yes
Unemploye	18	>9 months	Male	No	No	No	Not applica	Yes
Unemploye	22	>9 months	Female	No	No	No	< 24 hours/	Yes
Unemploye	17	>9 months	Male	No	No	No	Not applica	Yes
Unemploye	16	>9 months	Male	No	No	No	Not applica	Yes
Unemploye	17	>9 months	Male	No	No	No	Not applica	Yes
Unemploye	16	>9 months	Male	No	No	No	Not applica	Yes
Unemploye	20	>9 months	Male	No	No	No	Not applica	Yes
Emloyed	25	>9 months	Male	No	No	No	< 24 hours/	Yes
Emloyed	24	>9 months	Male	No	No	No	< 24 hours/	Yes
Emloyed	24	>9 months	Male	No	No	No	>48 hours/	Yes
Emloyed	24	>9 months	Male	No	No	No	>48 hours/	Yes
Emloyed	25	>9 months	Male	No	No	No	>48 hours/	Yes
Emloyed	48	>9 months	Female	No	No	No	Not applica	Yes
Emloyed	57	>9 months	Male	Yes	No	No	>48 hours/	Yes
Emloyed	57	>9 months	Male	Yes	No	No	>48 hours/	Yes
Emloyed	45	>9 months	Female	No	No	No	Not applica	Yes
Unemploye	18	>9 months	Female	No	No	No	Not applica	Yes
Emloyed	32	>9 months	Female	No	No	No	24-48 hour	Yes
Unemploye	18	>9 months	Female	No	No	No	Not applica	Yes
Unemploye	13	>9 months	Female	No	No	No	Not applica	Yes
Unemploye	16	>9 months	Female	No	No	No	Not applica	Yes
Unemploye	24	>9 months	Male	No	No	No	>48 hours/	Yes
Emloyed	40	>9 months	Male	No	No	No	24-48 hour	Yes

Emloyed	48	>9 months	Male	No	Yes	No	24-48 hour	Yes
Emloyed	70	>9 months	Male	No	No	No	Not applica	Yes
Emloyed	55	>9 months	Female	No	No	No	Not applica	Yes
Emloyed	46	>9 months	Female	No	No	No	24-48 hour	Yes
Unemploye	25	>9 months	Male	No	No	No	24-48 hour	Yes
Unemploye	15	>9 months	Female	No	No	No	Not applica	Yes
Unemploye	23	>9 months	Male	No	No	No	24-48 hour	Yes
Unemploye	18	>9 months	Male	Yes	No	Yes	Not applica	Yes
Unemploye	21	>9 months	Female	No	No	No	Not applica	Yes
Unemploye	16	>9 months	Male	No	Yes	No	Not applica	Yes
Emloyed	49	>9 months	Male	No	No	No	>48 hours/	Yes
Unemploye	35	>9 months	Female	No	No	No	>48 hours/	Yes
Unemploye	18	>9 months	Male	No	Yes	No	24-48 hour	Yes
Unemploye	23	>9 months	Male	No	No	No	24-48 hour	Yes
Unemploye	20	>9 months	Female	No	No	No	< 24 hours	Yes
Unemploye	40	>9 months	Female	No	No	No	< 24 hours	Yes
Emloyed	28	>9 months	Male	No	No	No	>48 hours/	Yes
Emloyed	27	>9 months	Male	No	No	No	>48 hours/	Yes
Unemploye	25	>9 months	Female	No	No	No	< 24 hours	Yes
Emloyed	28	>9 months	Male	No	No	No	>48 hours/	Yes
Unemploye	22	>9 months	Female	No	No	No	< 24 hours	Yes
Unemploye	17	>9 months	Female	No	No	No	< 24 hours	Yes
Emloyed	28	>9 months	Male	No	No	No	< 24 hours	Yes
Unemploye	18	>9 months	Female	No	No	No	< 24 hours	Yes
Unemploye	17	>9 months	Female	No	No	No	< 24 hours	Yes
Unemploye	17	>9 months	Female	No	No	No	< 24 hours	Yes
Unemploye	20	>9 months	Female	No	No	No	< 24 hours	Yes
Unemploye	18	>9 months	Female	No	No	No	< 24 hours	Yes
Unemploye	19	8-9 moths	Female	No	No	No	< 24 hours	Yes
Emloyed	40	>9 months	Male	No	No	No	< 24 hours	Yes
Unemploye	23	>9 months	Male	No	No	No	< 24 hours	Yes
Unemploye	49	>9 months	Female	Yes	No	No	< 24 hours	Yes
Unemploye	18	>9 months	Male	No	No	No	< 24 hours	Yes
Emloyed	26	>9 months	Male	No	Yes	No	< 24 hours	Yes
Unemploye	24	>9 months	Male	No	No	No	< 24 hours	Yes
Unemploye	24	>9 months	Male	No	No	No	< 24 hours	Yes
Unemploye	20	>9 months	Male	No	No	No	< 24 hours	Yes
Unemploye	21	>9 months	Female	No	No	No	< 24 hours	Yes
Unemploye	20	< 8 months	Female	No	No	No	< 24 hours	Yes
Unemploye	21	8-9 moths	Female	No	No	No	< 24 hours	Yes
Unemploye	20	8-9 moths	Female	No	No	No	< 24 hours	Yes
Unemploye	18	>9 months	Male	No	No	No	< 24 hours	Yes
Unemploye	20	>9 months	Female	No	Yes	No	< 24 hours	Yes
Unemploye	21	>9 months	Female	No	Yes	No	< 24 hours	Yes
Unemploye	21	>9 months	Female	No	No	No	< 24 hours	Yes
Unemploye	22	8-9 moths	Female	No	No	Yes	< 24 hours	Yes
Unemploye	22	>9 months	Female	No	No	No	24-48 hour	Yes
Unemploye	21	>9 months	Male	No	No	No	< 24 hours	Yes
Unemploye	21	>9 months	Female	No	No	No	< 24 hours	Yes
Unemploye	23	>9 months	Male	No	No	No	< 24 hours	Yes
Unemploye	21	>9 months	Female	No	No	No	< 24 hours	Yes
Unemploye	22	>9 months	Female	No	No	No	< 24 hours	Yes

Emloyed	24	>9 months	Male	No	No	No	24-48 hour	Yes
Unemploye	20	>9 months	Male	No	No	No	< 24 hours/	Yes
Unemploye	21	>9 months	Male	No	No	No	< 24 hours/	Yes
Unemploye	22	>9 months	Female	No	No	No	< 24 hours/	Yes
Unemploye	21	>9 months	Female	No	No	No	< 24 hours/	Yes
Unemploye	21	>9 months	Female	No	Yes	No	24-48 hour	Yes
Unemploye	22	>9 months	Female	No	No	No	< 24 hours/	Yes
Unemploye	20	>9 months	Female	No	No	Yes	< 24 hours/	Yes
Emloyed	25	>9 months	Male	No	No	No	24-48 hour	Yes
Unemploye	15	>9 months	Male	No	No	No	< 24 hours/	Yes
Emloyed	55	>9 months	Male	No	No	No	< 24 hours/	Yes
Unemploye	45	>9 months	Female	No	No	No	< 24 hours/	Yes
Unemploye	17	>9 months	Male	No	No	No	< 24 hours/	Yes
Unemploye	17	>9 months	Male	No	No	Yes	< 24 hours/	Yes
Unemploye	24	8-9 moths	Male	No	No	No	24-48 hour	Yes
Emloyed	25	>9 months	Male	No	No	No	< 24 hours/	Yes
Unemploye	19	>9 months	Female	No	No	No	< 24 hours/	Yes
Unemploye	20	>9 months	Female	No	No	No	< 24 hours/	Yes
Emloyed	48	>9 months	Male	No	No	No	< 24 hours/	Yes
Unemploye	45	>9 months	Female	No	No	No	< 24 hours/	Yes
Unemploye	50	>9 months	Female	No	No	No	< 24 hours/	Yes
Emloyed	55	>9 months	Male	No	No	No	< 24 hours/	Yes
Emloyed	50	>9 months	Male	No	No	No	>48 hours/	Yes
Emloyed	29	8-9 moths	Male	No	No	No	24-48 hour	Yes
Emloyed	38	8-9 moths	Male	No	No	No	< 24 hours/	Yes
Emloyed	49	>9 months	Male	No	No	No	< 24 hours/	Yes
Unemploye	21	>9 months	Female	No	No	No	< 24 hours/	Yes
Emloyed	31	>9 months	Male	No	Yes	No	24-48 hour	Yes
Unemploye	22	>9 months	Male	No	No	No	< 24 hours/	Yes
Unemploye	21	>9 months	Female	No	No	No	24-48 hour	Yes
Unemploye	22	>9 months	Female	No	No	No	< 24 hours/	Yes
Unemploye	22	>9 months	Female	No	No	No	< 24 hours/	Yes
Unemploye	21	>9 months	Female	No	No	No	< 24 hours/	Yes
Unemploye	23	8-9 moths	Male	No	No	No	< 24 hours/	Yes
Unemploye	23	8-9 moths	Male	No	No	No	< 24 hours/	Yes
Unemploye	20	>9 months	Female	No	No	No	< 24 hours/	Yes
Emloyed	31	>9 months	Female	No	No	No	< 24 hours/	Yes
Emloyed	32	>9 months	Female	No	No	Yes	< 24 hours/	Yes
Emloyed	43	>9 months	Male	No	No	No	>48 hours/	Yes
Unemploye	21	>9 months	Male	No	No	No	< 24 hours/	Yes
Unemploye	18	>9 months	Male	No	No	No	24-48 hour	Yes
Unemploye	21	>9 months	Male	No	No	No	< 24 hours/	Yes
Emloyed	28	8-9 moths	Male	No	No	No	24-48 hour	Yes
Emloyed	26	>9 months	Male	No	Yes	No	24-48 hour	Yes
Unemploye	27	>9 months	Male	No	No	No	< 24 hours/	Yes
Unemploye	28	>9 months	Male	No	Yes	No	< 24 hours/	Yes
Unemploye	22	>9 months	Male	No	No	No	24-48 hour	Yes
Emloyed	34	8-9 moths	Male	No	No	No	>48 hours/	Yes
Unemploye	27	>9 months	Male	No	No	No	24-48 hour	Yes
Emloyed	39	>9 months	Male	No	No	No	< 24 hours/	Yes
Emloyed	29	8-9 moths	Male	No	No	No	>48 hours/	Yes
Emloyed	29	>9 months	Male	No	No	No	24-48 hour	Yes

Emloyed	31	>9 months	Male	No	No	No	24-48 hour	Yes
Emloyed	28	>9 months	Male	No	No	No	>48 hours/	Yes
Emloyed	39	>9 months	Male	No	No	No	24-48 hour	Yes
Emloyed	26	>9 months	Female	No	Yes	No	24-48 hour	Yes
Unemploye	28	8-9 moths	Female	Yes	Yes	Yes	< 24 hours	Yes
Emloyed	28	>9 months	Male	No	Yes	No	>48 hours/	Yes
Unemploye	33	>9 months	Female	No	No	No	< 24 hours	Yes
Unemploye	22	>9 months	Male	No	No	No	< 24 hours	Yes
Unemploye	21	>9 months	Male	No	No	No	< 24 hours	Yes
Unemploye	29	>9 months	Male	No	No	No	< 24 hours	Yes
Unemploye	20	>9 months	Female	No	No	No	< 24 hours	Yes
Unemploye	23	>9 months	Male	No	No	No	< 24 hours	Yes
Unemploye	24	>9 months	Female	No	No	No	< 24 hours	Yes
Unemploye	25	>9 months	Female	No	No	No	< 24 hours	Yes
Unemploye	24	< 8 months	Male	No	No	No	< 24 hours	Yes
Unemploye	22	8-9 moths	Female	No	No	No	>48 hours/	Yes
Unemploye	19	8-9 moths	Female	No	No	No	24-48 hour	Yes
Unemploye	21	8-9 moths	Male	No	No	No	< 24 hours	Yes
Emloyed	23	>9 months	Male	No	Yes	No	< 24 hours	Yes
Unemploye	20	>9 months	Male	No	No	No	< 24 hours	Yes
Unemploye	24	< 8 months	Male	No	No	No	< 24 hours	Yes
Unemploye	20	>9 months	Male	No	No	No	< 24 hours	Yes
Unemploye	21	8-9 moths	Male	No	No	No	< 24 hours	Yes
Unemploye	22	>9 months	Female	No	No	No	< 24 hours	Yes
Unemploye	21	8-9 moths	Male	No	No	No	>48 hours/	Yes
Unemploye	23	>9 months	Male	No	No	No	< 24 hours	Yes
Unemploye	19	>9 months	Female	No	Yes	No	< 24 hours	Yes
Emloyed	24	>9 months	Male	No	No	No	24-48 hour	Yes
Unemploye	21	>9 months	Female	No	Yes	No	< 24 hours	Yes
Unemploye	20	>9 months	Female	No	Yes	No	< 24 hours	Yes
Unemploye	24	>9 months	Male	No	No	No	< 24 hours	Yes
Emloyed	31	>9 months	Male	No	No	No	24-48 hour	Yes
Emloyed	24	>9 months	Male	No	No	No	< 24 hours	Yes
Unemploye	21	>9 months	Male	No	No	No	< 24 hours	Yes
Emloyed	29	>9 months	Male	No	No	No	< 24 hours	Yes
Emloyed	27	>9 months	Male	No	Yes	No	>48 hours/	Yes
Emloyed	31	>9 months	Male	No	No	No	>48 hours/	Yes
Emloyed	45	< 8 months	Male	No	No	No	>48 hours/	Yes
Emloyed	28	< 8 months	Female	No	No	No	< 24 hours	Yes
Emloyed	31	>9 months	Male	No	No	No	< 24 hours	Yes
Emloyed	36	>9 months	Male	No	No	No	>48 hours/	Yes
Unemploye	23	8-9 moths	Male	No	No	No	< 24 hours	Yes
Emloyed	49	>9 months	Male	No	No	No	< 24 hours	Yes
Unemploye	20	>9 months	Female	No	Yes	No	< 24 hours	Yes
Unemploye	21	>9 months	Male	No	No	No	< 24 hours	Yes
Unemploye	23	>9 months	Male	No	Yes	No	24-48 hour	Yes
Unemploye	18	>9 months	Female	Yes	No	No	< 24 hours	Yes
Emloyed	39	>9 months	Male	No	No	No	< 24 hours	Yes
Unemploye	23	>9 months	Female	No	Yes	No	>48 hours/	Yes
Unemploye	25	>9 months	Female	No	Yes	No	>48 hours/	Yes
Unemploye	19	>9 months	Female	No	No	No	< 24 hours	Yes
Unemploye	20	>9 months	Female	No	No	No	24-48 hour	Yes

Emloyed	28	>9 months	Male	No	No	No	24-48 hour	Yes
Emloyed	34	>9 months	Female	No	No	Yes	24-48 hour	Yes
Emloyed	36	8-9 moths	Female	Yes	No	No	24-48 hour	Yes
Emloyed	45	8-9 moths	Female	No	No	No	>48 hours/	Yes
Emloyed	28	8-9 moths	Male	No	No	No	< 24 hours	Yes
Unemploye	19	>9 months	Female	No	No	No	< 24 hours	Yes
Unemploye	16	>9 months	Male	No	No	No	< 24 hours	Yes
Unemploye	17	>9 months	Female	No	No	No	< 24 hours	Yes
Unemploye	37	>9 months	Female	No	Yes	No	< 24 hours	Yes
Unemploye	35	>9 months	Female	No	No	No	< 24 hours	Yes
Unemploye	21	>9 months	Female	No	Yes	No	< 24 hours	Yes
Unemploye	28	>9 months	Female	No	No	No	< 24 hours	Yes
Unemploye	30	>9 months	Female	No	No	No	< 24 hours	Yes
Unemploye	17	>9 months	Female	No	No	No	< 24 hours	Yes
Unemploye	18	>9 months	Female	No	No	No	< 24 hours	Yes
Unemploye	32	>9 months	Female	No	Yes	No	< 24 hours	Yes
Unemploye	40	>9 months	Female	No	No	No	< 24 hours	Yes
Unemploye	19	>9 months	Female	No	No	No	< 24 hours	Yes
Unemploye	35	>9 months	Female	No	No	No	< 24 hours	Yes
Emloyed	26	>9 months	Female	No	No	No	>48 hours/	Yes
Unemploye	60	>9 months	Female	No	No	No	< 24 hours	Yes
Emloyed	35	>9 months	Female	No	No	No	>48 hours/	Yes
Unemploye	19	>9 months	Female	No	No	No	< 24 hours	Yes
Unemploye	30	>9 months	Female	No	No	No	< 24 hours	Yes
Unemploye	21	8-9 moths	Male	No	No	No	< 24 hours	Yes
Unemploye	19	8-9 moths	Female	No	No	No	< 24 hours	Yes
Unemploye	20	>9 months	Female	No	No	Yes	< 24 hours	Yes
Unemploye	20	8-9 moths	Male	No	No	No	< 24 hours	Yes
Unemploye	24	>9 months	Male	No	No	No	24-48 hour	Yes
Unemploye	24	8-9 moths	Female	No	No	Yes	< 24 hours	Yes
Emloyed	38	>9 months	Female	No	No	No	< 24 hours	Yes
Emloyed	43	>9 months	Male	Yes	No	No	< 24 hours	Yes
Emloyed	22	>9 months	Male	No	No	No	24-48 hour	Yes
Emloyed	31	>9 months	Male	No	No	Yes	24-48 hour	Yes
Emloyed	28	>9 months	Male	No	Yes	No	24-48 hour	Yes
Unemploye	20	>9 months	Female	No	No	No	24-48 hour	Yes
Unemploye	21	>9 months	Female	No	No	No	< 24 hours	Yes
Emloyed	24	>9 months	Male	No	Yes	No	>48 hours/	Yes
Emloyed	31	>9 months	Male	Yes	No	No	24-48 hour	Yes
Emloyed	45	>9 months	Female	Yes	No	Yes	24-48 hour	Yes
Unemploye	23	>9 months	Male	No	No	No	< 24 hours	Yes
Emloyed	59	>9 months	Female	Yes	No	Yes	24-48 hour	Yes
Unemploye	68	>9 months	Male	Yes	No	No	< 24 hours	Yes
Emloyed	61	8-9 moths	Male	Yes	No	No	< 24 hours	Yes
Emloyed	45	8-9 moths	Female	Yes	No	No	< 24 hours	Yes
Unemploye	46	>9 months	Female	No	No	Yes	< 24 hours	Yes
Unemploye	23	8-9 moths	Female	No	No	No	< 24 hours	Yes
Emloyed	50	>9 months	Male	Yes	No	No	24-48 hour	Yes
Emloyed	68	>9 months	Female	Yes	No	No	24-48 hour	Yes
Emloyed	37	>9 months	Female	No	No	No	< 24 hours	Yes
Unemploye	20	>9 months	Female	No	No	Yes	< 24 hours	Yes
Unemploye	22	8-9 moths	Female	No	No	No	< 24 hours	Yes

Emloyed	54	8-9 moths	Male	Yes	No	No	< 24 hours,	Yes
Unemploye	21	>9 months	Female	No	No	No	< 24 hours,	Yes
Unemploye	23	>9 months	Female	No	No	No	< 24 hours,	Yes
Emloyed	32	>9 months	Male	No	No	No	>48 hours/	Yes
Unemploye	26	>9 months	Female	No	No	Yes	< 24 hours,	Yes
Emloyed	55	>9 months	Male	No	No	No	< 24 hours,	Yes
Unemploye	16	>9 months	Male	No	Yes	No	< 24 hours,	Yes
Unemploye	24	>9 months	Male	No	No	No	< 24 hours,	Yes
Emloyed	51	>9 months	Female	No	No	No	24-48 hour	Yes
Emloyed	27	>9 months	Female	No	No	Yes	>48 hours/	Yes
Emloyed	45	>9 months	Female	No	No	No	24-48 hour	Yes
Emloyed	31	>9 months	Male	No	No	No	>48 hours/	Yes
Emloyed	27	>9 months	Male	No	No	No	>48 hours/	Yes
Emloyed	55	>9 months	Male	Yes	No	No	< 24 hours,	Yes
Unemploye	50	>9 months	Female	Yes	No	No	< 24 hours,	Yes
Unemploye	22	>9 months	Male	No	No	No	< 24 hours,	Yes
Unemploye	40	>9 months	Female	No	No	Yes	< 24 hours,	Yes
Unemploye	43	>9 months	Female	Yes	No	Yes	< 24 hours,	Yes
Emloyed	59	>9 months	Male	Yes	No	No	24-48 hour	Yes
Emloyed	26	>9 months	Male	No	No	No	>48 hours/	Yes
Unemploye	46	>9 months	Female	No	No	No	< 24 hours,	Yes
Emloyed	52	>9 months	Male	Yes	No	No	24-48 hour	Yes
Unemploye	49	>9 months	Female	No	No	No	< 24 hours,	Yes
Unemploye	44	>9 months	Female	No	No	Yes	< 24 hours,	Yes
Emloyed	54	>9 months	Male	Yes	No	No	>48 hours/	Yes
Unemploye	18	>9 months	Female	No	No	No	< 24 hours,	Yes
Unemploye	41	>9 months	Female	No	No	No	< 24 hours,	Yes
Emloyed	36	>9 months	Male	Yes	No	Yes	>48 hours/	Yes
Emloyed	39	>9 months	Male	No	No	No	24-48 hour	Yes
Emloyed	52	>9 months	Male	No	No	No	>48 hours/	Yes
Emloyed	45	>9 months	Male	No	Yes	No	>48 hours/	Yes
Unemploye	24	>9 months	Male	No	No	No	>48 hours/	Yes
Unemploye	65	>9 months	Male	No	No	No	Not applica	Yes
Unemploye	51	>9 months	Female	Yes	No	No	< 24 hours,	Yes
Emloyed	38	>9 months	Male	No	Yes	No	>48 hours/	Yes
Emloyed	26	8-9 moths	Female	No	No	No	< 24 hours,	Yes
Emloyed	57	>9 months	Female	Yes	No	Yes	>48 hours/	Yes
Unemploye	18	>9 months	Female	No	No	No	24-48 hour	Yes
Emloyed	43	>9 months	Male	No	No	No	>48 hours/	Yes
Emloyed	48	>9 months	Male	No	No	No	>48 hours/	Yes
Emloyed	38	>9 months	Female	No	No	No	>48 hours/	Yes
Emloyed	42	>9 months	Male	No	No	No	>48 hours/	Yes
Emloyed	37	>9 months	Male	No	No	No	>48 hours/	Yes
Emloyed	48	>9 months	Male	No	No	No	>48 hours/	Yes
Emloyed	36	>9 months	Male	No	No	No	24-48 hour	Yes
Emloyed	40	>9 months	Male	No	No	No	>48 hours/	Yes
Unemploye	35	>9 months	Female	No	No	No	< 24 hours,	Yes
Emloyed	37	>9 months	Female	No	No	No	24-48 hour	Yes
Unemploye	39	>9 months	Female	No	No	No	24-48 hour	Yes
Unemploye	55	>9 months	Female	Yes	No	No	< 24 hours,	Yes
Unemploye	51	>9 months	Female	No	No	Yes	< 24 hours,	Yes
Emloyed	52	>9 months	Male	No	No	Yes	>48 hours/	Yes

Emloyed	26	>9 months	Male	No	No	No	>48 hours/	Yes
Unemploye	22	>9 months	Female	No	Yes	No	Not applica	Yes
Unemploye	41	>9 months	Female	No	No	No	< 24 hours/	Yes
Emloyed	45	>9 months	Male	No	No	No	24-48 hour	Yes
Emloyed	58	>9 months	Male	Yes	No	No	>48 hours/	Yes
Unemploye	45	>9 months	Female	No	No	No	< 24 hours/	Yes
Unemploye	60	>9 months	Male	Yes	No	No	< 24 hours/	Yes
Unemploye	55	>9 months	Female	No	No	No	< 24 hours/	Yes
Unemploye	24	>9 months	Male	No	Yes	No	>48 hours/	Yes
Emloyed	49	>9 months	Male	No	No	No	24-48 hour	Yes
Emloyed	55	>9 months	Male	No	No	No	24-48 hour	Yes
Emloyed	31	>9 months	Male	No	No	No	24-48 hour	Yes
Unemploye	22	8-9 moths	Female	No	No	No	24-48 hour	Yes
Unemploye	22	8-9 moths	Female	No	No	No	< 24 hours/	Yes
Unemploye	21	8-9 moths	Female	No	Yes	No	< 24 hours/	Yes
Unemploye	22	8-9 moths	Female	No	No	No	24-48 hour	Yes
Unemploye	22	8-9 moths	Female	No	No	No	< 24 hours/	Yes
Unemploye	24	8-9 moths	Male	No	No	No	< 24 hours/	Yes
Unemploye	27	>9 months	Male	No	No	No	>48 hours/	Yes
Unemploye	22	8-9 moths	Female	No	No	No	Not applica	Yes
Emloyed	27	>9 months	Male	No	No	No	>48 hours/	Yes
Unemploye	20	8-9 moths	Male	No	No	No	24-48 hour	Yes
Unemploye	21	>9 months	Male	No	No	No	< 24 hours/	Yes
Unemploye	21	>9 months	Male	No	No	No	Not applica	Yes
Unemploye	18	>9 months	Male	No	No	No	Not applica	Yes
Emloyed	21	>9 months	Male	No	No	No	< 24 hours/	Yes
Unemploye	23	8-9 moths	Female	No	No	No	Not applica	Yes
Unemploye	15	>9 months	Male	No	Yes	No	< 24 hours/	Yes
Unemploye	32	>9 months	Female	No	Yes	No	Not applica	Yes
Emloyed	35	>9 months	Male	No	Yes	No	>48 hours/	Yes
Unemploye	15	>9 months	Male	No	Yes	No	< 24 hours/	Yes
Unemploye	9	>9 months	Female	No	No	No	>48 hours/	Yes
Unemploye	30	>9 months	Female	Yes	No	No	>48 hours/	Yes
Unemploye	14	>9 months	Male	No	No	No	Not applica	Yes
Emloyed	33	>9 months	Male	No	Yes	No	>48 hours/	Yes
Unemploye	13	>9 months	Female	No	No	No	< 24 hours/	Yes
Unemploye	16	>9 months	Male	No	No	No	Not applica	Yes
Unemploye	16	>9 months	Male	No	No	No	Not applica	Yes
Unemploye	52	>9 months	Female	No	No	No	< 24 hours/	Yes
Unemploye	60	>9 months	Female	No	No	No	< 24 hours/	Yes
Unemploye	50	>9 months	Female	No	No	Yes	< 24 hours/	Yes
Emloyed	60	>9 months	Male	Yes	No	No	>48 hours/	Yes
Unemploye	70	>9 months	Female	Yes	No	No	< 24 hours/	Yes
Unemploye	75	>9 months	Female	Yes	No	No	< 24 hours/	Yes
Unemploye	48	>9 months	Female	Yes	No	No	< 24 hours/	Yes
Emloyed	52	>9 months	Male	Yes	No	No	24-48 hour	Yes
Emloyed	46	>9 months	Male	Yes	No	Yes	>48 hours/	Yes
Emloyed	39	>9 months	Male	No	No	No	>48 hours/	Yes
Emloyed	37	>9 months	Male	No	No	No	>48 hours/	Yes
Emloyed	39	>9 months	Male	No	No	No	>48 hours/	Yes
Emloyed	42	>9 months	Female	Yes	No	No	>48 hours/	Yes
Emloyed	47	>9 months	Male	No	No	No	>48 hours/	Yes

Emloyed	48	>9 months	Male	No	No	No	24-48 hour	Yes
Emloyed	38	>9 months	Male	No	No	No	>48 hours/	Yes
Unemploye	9	>9 months	Female	No	No	No	< 24 hours	Yes
Emloyed	58	>9 months	Male	No	Yes	No	>48 hours/	Yes
Emloyed	51	>9 months	Male	Yes	No	No	>48 hours/	Yes
Emloyed	55	>9 months	Male	No	No	Yes	>48 hours/	Yes
Emloyed	53	>9 months	Male	Yes	No	No	>48 hours/	Yes
Emloyed	55	>9 months	Female	No	No	No	>48 hours/	Yes
Emloyed	52	>9 months	Male	Yes	No	No	24-48 hour	Yes
Emloyed	44	>9 months	Male	Yes	No	No	>48 hours/	Yes
Emloyed	58	>9 months	Female	Yes	No	No	>48 hours/	Yes
Emloyed	51	>9 months	Male	No	No	No	>48 hours/	Yes
Emloyed	57	>9 months	Male	No	Yes	No	>48 hours/	Yes
Unemploye	28	>9 months	Female	No	No	No	Not applica	Yes
Unemploye	24	8-9 moths	Male	No	No	No	Not applica	Yes
Emloyed	26	>9 months	Male	No	No	No	>48 hours/	Yes
Unemploye	40	8-9 moths	Female	No	No	No	Not applica	Yes
Emloyed	51	8-9 moths	Male	Yes	No	Yes	< 24 hours	Yes
Emloyed	25	8-9 moths	Male	No	No	Yes	< 24 hours	Yes
Unemploye	21	8-9 moths	Female	No	No	Yes	< 24 hours	Yes
Unemploye	17	8-9 moths	Male	No	No	No	24-48 hour	Yes
Unemploye	20	8-9 moths	Female	No	No	No	24-48 hour	Yes
Unemploye	17	8-9 moths	Female	No	Yes	No	Not applica	Yes
Unemploye	22	8-9 moths	Female	No	No	No	< 24 hours	Yes
Emloyed	54	8-9 moths	Male	Yes	No	No	24-48 hour	Yes
Emloyed	42	8-9 moths	Male	Yes	No	No	< 24 hours	Yes
Emloyed	40	>9 months	Male	No	No	No	< 24 hours	Yes
Unemploye	13	8-9 moths	Male	No	No	No	Not applica	Yes
Emloyed	50	>9 months	Female	No	No	No	< 24 hours	Yes
Unemploye	23	8-9 moths	Female	No	No	No	< 24 hours	Yes
Emloyed	55	8-9 moths	Male	Yes	No	No	< 24 hours	Yes
Unemploye	21	8-9 moths	Female	No	No	No	Not applica	Yes
Emloyed	28	8-9 moths	Male	No	No	No	< 24 hours	Yes
Emloyed	35	< 8 months	Female	No	No	No	< 24 hours	Yes
Emloyed	40	>9 months	Male	No	No	No	< 24 hours	Yes
Emloyed	46	8-9 moths	Male	Yes	No	No	< 24 hours	Yes
Emloyed	45	8-9 moths	Male	No	No	No	< 24 hours	Yes
Emloyed	35	8-9 moths	Male	Yes	No	No	< 24 hours	Yes
Emloyed	50	8-9 moths	Female	No	No	No	>48 hours/	Yes
Emloyed	30	8-9 moths	Male	No	No	No	< 24 hours	Yes
Emloyed	30	8-9 moths	Female	No	No	No	< 24 hours	Yes
Emloyed	38	8-9 moths	Female	No	No	Yes	< 24 hours	Yes
Emloyed	40	8-9 moths	Male	No	No	No	< 24 hours	Yes
Unemploye	18	8-9 moths	Female	No	No	No	< 24 hours	Yes
Unemploye	19	8-9 moths	Female	No	No	No	< 24 hours	Yes
Unemploye	21	8-9 moths	Female	No	No	No	< 24 hours	Yes
Unemploye	18	8-9 moths	Female	No	No	No	Not applica	Yes
Emloyed	40	>9 months	Female	No	No	No	24-48 hour	Yes
Emloyed	32	>9 months	Male	No	No	No	>48 hours/	Yes
Emloyed	30	>9 months	Female	No	No	No	< 24 hours	Yes
Unemploye	21	8-9 moths	Female	No	No	No	Not applica	Yes
Unemploye	23	>9 months	Male	No	No	No	< 24 hours	Yes

Emloyed	24	8-9 moths	Male	No	No	No	>48 hours/\	Yes
Unemploye	24	8-9 moths	Male	No	No	Yes	< 24 hours,	Yes
Unemploye	22	8-9 moths	Male	No	No	No	< 24 hours,	Yes
Unemploye	23	8-9 moths	Female	No	No	No	Not applica	Yes
Unemploye	21	>9 months	Female	No	No	No	Not applica	Yes
Unemploye	15	8-9 moths	Male	No	No	No	< 24 hours,	Yes
Emloyed	50	>9 months	Male	No	No	No	24-48 hour	Yes
Unemploye	42	>9 months	Female	No	No	No	< 24 hours,	Yes
Emloyed	29	8-9 moths	Female	No	Yes	Yes	< 24 hours,	Yes
Unemploye	20	8-9 moths	Female	No	No	Yes	< 24 hours,	Yes
Unemploye	20	8-9 moths	Female	No	No	No	Not applica	Yes
Unemploye	20	8-9 moths	Female	No	No	No	Not applica	Yes
Unemploye	23	8-9 moths	Male	No	No	No	< 24 hours,	Yes
Unemploye	21	< 8 months	Female	No	No	No	Not applica	Yes
Unemploye	18	>9 months	Male	No	No	No	Not applica	Yes
Unemploye	25	>9 months	Female	No	No	No	< 24 hours,	Yes
Unemploye	23	>9 months	Female	No	No	No	Not applica	Yes
Unemploye	21	>9 months	Female	No	No	Yes	24-48 hour	Yes
Emloyed	50	>9 months	Male	No	No	No	>48 hours/\	Yes
Emloyed	40	>9 months	Female	No	No	No	24-48 hour	Yes
Emloyed	29	>9 months	Male	No	No	No	>48 hours/\	Yes
Unemploye	18	>9 months	Male	No	No	No	< 24 hours,	Yes
Emloyed	26	>9 months	Male	No	No	Yes	24-48 hour	Yes
Emloyed	27	>9 months	Male	No	No	No	>48 hours/\	Yes
Emloyed	25	8-9 moths	Male	No	No	No	>48 hours/\	Yes
Unemploye	14	>9 months	Male	No	No	No	24-48 hour	Yes
Emloyed	24	>9 months	Female	No	No	No	>48 hours/\	Yes
Unemploye	23	>9 months	Female	No	No	No	< 24 hours,	Yes
Unemploye	21	>9 months	Male	No	No	Yes	Not applica	Yes
Unemploye	23	>9 months	Male	No	Yes	No	< 24 hours,	Yes
Unemploye	23	>9 months	Male	No	No	No	Not applica	Yes
Emloyed	66	>9 months	Female	No	No	No	< 24 hours,	Yes
Unemploye	68	>9 months	Male	Yes	No	No	< 24 hours,	Yes
Unemploye	18	>9 months	Female	No	No	No	< 24 hours,	Yes
Unemploye	82	>9 months	Female	No	No	No	Not applica	Yes
Emloyed	28	8-9 moths	Female	No	No	No	>48 hours/\	Yes
Unemploye	22	>9 months	Female	No	No	No	< 24 hours,	Yes
Unemploye	22	>9 months	Female	No	No	No	Not applica	Yes
Unemploye	17	>9 months	Female	No	Yes	No	< 24 hours,	Yes
Unemploye	21	>9 months	Male	No	No	No	< 24 hours,	Yes
Unemploye	21	>9 months	Male	No	No	No	< 24 hours,	Yes
Unemploye	21	>9 months	Male	No	No	No	< 24 hours,	Yes
Unemploye	18	8-9 moths	Male	No	No	No	< 24 hours,	Yes
Unemploye	19	8-9 moths	Male	No	No	No	Not applica	Yes
Unemploye	49	>9 months	Female	No	No	No	Not applica	Yes
Unemploye	24	>9 months	Male	No	No	No	Not applica	Yes
Unemploye	23	>9 months	Male	No	No	No	Not applica	Yes
Unemploye	18	>9 months	Male	No	No	No	< 24 hours,	Yes
Unemploye	23	>9 months	Male	No	No	No	< 24 hours,	Yes
Emloyed	31	>9 months	Male	No	Yes	No	24-48 hour	Yes
Unemploye	21	>9 months	Male	No	Yes	No	Not applica	Yes
Unemploye	17	>9 months	Male	No	No	No	24-48 hour	Yes

Unemploye	51	>9 months	Female	No	No	No	Not applica	Yes
Unemploye	18	>9 months	Male	No	No	No	< 24 hours/	Yes
Unemploye	24	>9 months	Male	No	No	No	Not applica	Yes
Unemploye	21	< 8 months	Male	No	No	No	< 24 hours/	Yes
Unemploye	26	< 8 months	Male	No	Yes	No	< 24 hours/	Yes
Unemploye	22	8-9 moths	Male	No	No	No	Not applica	Yes
Unemploye	25	< 8 months	Male	No	No	No	< 24 hours/	Yes
Emloyed	48	8-9 moths	Male	Yes	No	No	24-48 hour	Yes
Unemploye	46	8-9 moths	Female	Yes	No	No	Not applica	Yes
Unemploye	26	8-9 moths	Female	No	No	No	< 24 hours/	Yes
Unemploye	22	8-9 moths	Female	No	No	No	< 24 hours/	Yes
Unemploye	28	>9 months	Female	No	No	Yes	Not applica	Yes
Emloyed	25	>9 months	Male	No	No	No	>48 hours/	Yes
Emloyed	26	>9 months	Male	No	No	No	>48 hours/	Yes
Emloyed	41	>9 months	Male	No	No	No	>48 hours/	Yes
Unemploye	22	>9 months	Male	No	No	No	< 24 hours/	Yes
Unemploye	21	< 8 months	Male	No	No	No	< 24 hours/	Yes
Unemploye	18	>9 months	Female	No	No	No	< 24 hours/	Yes
Unemploye	22	< 8 months	Female	No	No	No	< 24 hours/	Yes
Unemploye	21	>9 months	Female	No	No	No	< 24 hours/	Yes
Unemploye	21	>9 months	Female	No	No	No	Not applica	Yes
Unemploye	21	>9 months	Male	No	No	Yes	24-48 hour	Yes
Unemploye	46	>9 months	Female	No	No	No	< 24 hours/	Yes
Emloyed	53	>9 months	Male	No	No	No	24-48 hour	Yes
Unemploye	37	>9 months	Female	No	No	No	< 24 hours/	Yes
Unemploye	48	>9 months	Female	Yes	Yes	No	< 24 hours/	Yes
Unemploye	60	>9 months	Male	Yes	No	No	>48 hours/	Yes
Unemploye	23	8-9 moths	Female	No	No	No	< 24 hours/	Yes
Emloyed	35	8-9 moths	Female	No	No	No	< 24 hours/	Yes
Unemploye	57	>9 months	Female	Yes	No	Yes	< 24 hours/	Yes
Emloyed	47	>9 months	Male	No	No	No	24-48 hour	Yes
Unemploye	42	>9 months	Female	No	Yes	Yes	< 24 hours/	Yes
Emloyed	45	>9 months	Male	No	No	No	24-48 hour	Yes
Unemploye	30	>9 months	Female	No	No	No	Not applica	Yes
Emloyed	38	>9 months	Male	No	No	No	24-48 hour	Yes
Unemploye	29	>9 months	Female	No	No	No	Not applica	Yes
Unemploye	45	>9 months	Female	No	No	No	Not applica	Yes
Unemploye	55	>9 months	Female	No	No	No	Not applica	Yes
Unemploye	25	>9 months	Male	No	No	No	24-48 hour	Yes
Unemploye	12	>9 months	Female	No	No	No	Not applica	Yes
Unemploye	25	>9 months	Female	No	No	No	Not applica	Yes
Unemploye	30	>9 months	Female	No	No	No	Not applica	Yes
Emloyed	31	>9 months	Female	No	No	No	>48 hours/	Yes
Emloyed	38	< 8 months	Female	No	No	No	>48 hours/	Yes
Unemploye	15	< 8 months	Female	No	No	No	Not applica	Yes
Emloyed	28	>9 months	Male	No	No	No	>48 hours/	Yes
Emloyed	65	>9 months	Male	No	No	No	>48 hours/	Yes
Emloyed	48	>9 months	Female	No	No	No	24-48 hour	Yes
Emloyed	40	>9 months	Female	No	No	No	24-48 hour	Yes
Emloyed	56	8-9 moths	Male	No	No	No	>48 hours/	Yes
Unemploye	21	>9 months	Female	No	No	No	Not applica	Yes
Unemploye	22	>9 months	Male	No	No	No	Not applica	Yes

Emloyed	52	8-9 moths	Male	No	Yes	No	>48 hours/	Yes
Emloyed	52	8-9 moths	Male	No	No	No	>48 hours/	Yes
Emloyed	50	8-9 moths	Male	No	No	No	>48 hours/	Yes
Emloyed	49	8-9 moths	Male	No	No	No	24-48 hour	Yes
Emloyed	48	8-9 moths	Male	Yes	No	Yes	>48 hours/	Yes
Emloyed	48	8-9 moths	Male	No	No	No	>48 hours/	Yes
Emloyed	50	8-9 moths	Male	No	No	No	>48 hours/	Yes
Emloyed	38	8-9 moths	Female	Yes	No	No	>48 hours/	Yes
Emloyed	52	8-9 moths	Male	No	No	No	>48 hours/	Yes
Emloyed	39	8-9 moths	Male	No	Yes	No	>48 hours/	Yes
Emloyed	42	8-9 moths	Male	Yes	No	No	>48 hours/	Yes
Emloyed	44	8-9 moths	Male	Yes	No	No	>48 hours/	Yes
Emloyed	44	8-9 moths	Male	No	No	No	>48 hours/	Yes
Emloyed	44	8-9 moths	Male	Yes	No	No	>48 hours/	Yes
Emloyed	37	8-9 moths	Male	No	No	No	>48 hours/	Yes
Emloyed	38	8-9 moths	Male	No	No	No	>48 hours/	Yes
Emloyed	38	8-9 moths	Male	No	No	No	>48 hours/	Yes
Emloyed	44	8-9 moths	Male	No	No	Yes	>48 hours/	Yes
Emloyed	44	8-9 moths	Male	Yes	No	Yes	24-48 hour	Yes
Emloyed	44	8-9 moths	Male	No	No	No	>48 hours/	Yes
Emloyed	31	>9 months	Male	No	No	No	>48 hours/	Yes
Emloyed	46	8-9 moths	Male	No	No	No	>48 hours/	Yes
Emloyed	55	8-9 moths	Male	No	No	No	>48 hours/	Yes
Emloyed	8	8-9 moths	Male	Yes	No	No	>48 hours/	Yes

surgical	kn95	n95	others	cotton_mas	duration	perweek	mask_resu	income
Yes	No	No	No	No	> 12 month	< 8 hours	Single use	40000
Yes	No	No	No	No	> 12 month	> 32 hours	Single use	40000
Yes	No	No	No	No	> 12 month	> 32 hours	Single use	40000
Yes	Yes	No	No	No	> 12 month	8-32 hours	Single use	40000
Yes	Yes	No	No	No	> 12 month	8-32 hours	Single use	40000
Yes	No	No	No	No	> 12 month	< 8 hours	Use after c	30000
Yes	No	Yes	No	Yes	> 12 month	< 8 hours	Use after c	50000
No	No	No	No	Yes	> 12 month	< 8 hours	Use after c	30000
Yes	No	No	No	Yes	9-12 month	< 8 hours	Single use	40000
Yes	No	No	No	No	> 12 month	> 32 hours	Single use	35000
Yes	No	No	No	No	> 12 month	< 8 hours	Single use	30000
No	No	No	No	Yes	> 12 month	< 8 hours	Use after c	60000
Yes	Yes	No	No	No	> 12 month	8-32 hours	Use after c	50000
Yes	No	No	No	Yes	> 12 month	< 8 hours	Use after c	100000
Yes	Yes	No	No	No	> 12 month	< 8 hours	Single use	20000
No	No	No	No	Yes	> 12 month	< 8 hours	Use after c	15000
Yes	No	No	No	No	> 12 month	8-32 hours	Use withou	40000
Yes	Yes	No	Yes	Yes	9-12 month	< 8 hours	Use after c	30000
Yes	No	No	No	No	> 12 month	8-32 hours	Use after c	40000
Yes	No	No	No	No	> 12 month	8-32 hours	Use after c	30000
Yes	Yes	No	No	No	> 12 month	< 8 hours	Single use	100000
No	No	Yes	No	No	> 12 month	< 8 hours	Use after c	35000
Yes	No	No	No	Yes	> 12 month	8-32 hours	Use after c	40000
Yes	Yes	No	No	No	> 12 month	> 32 hours	Single use	20000
Yes	No	No	No	No	> 12 month	8-32 hours	Use after c	20000
Yes	No	No	No	No	> 12 month	8-32 hours	Use after c	50000
Yes	No	No	No	No	> 12 month	< 8 hours	Single use	50000
Yes	No	Yes	No	Yes	> 12 month	< 8 hours	Use after c	500000
Yes	No	No	No	Yes	9-12 month	< 8 hours	Use withou	200000
Yes	Yes	Yes	Yes	Yes	> 12 month	8-32 hours	Use after c	45000
No	No	No	No	Yes	> 12 month	8-32 hours	Use after c	5000
Yes	No	No	No	Yes	> 12 month	< 8 hours	Use after c	50000
Yes	No	No	No	No	> 12 month	< 8 hours	Use after c	40000
Yes	No	No	No	No	> 12 month	< 8 hours	Single use	40000
Yes	No	No	No	No	> 12 month	8-32 hours	Single use	70000
Yes	No	No	No	No	> 12 month	< 8 hours	Use after c	40000
Yes	Yes	No	No	Yes	> 12 month	< 8 hours	Single use	100000
Yes	No	No	No	No	> 12 month	< 8 hours	Single use	35000
Yes	No	No	No	Yes	9-12 month	< 8 hours	Use after c	40000
No	No	No	No	Yes	> 12 month	8-32 hours	Use after c	18000
Yes	No	No	No	No	> 12 month	< 8 hours	Single use	25000
No	No	No	No	Yes	> 12 month	> 32 hours	Use after c	50000
Yes	No	No	No	No	> 12 month	< 8 hours	Single use	30000
No	No	No	No	Yes	> 12 month	< 8 hours	Use after c	50000
Yes	No	No	No	Yes	> 12 month	8-32 hours	Use after c	50000
Yes	Yes	Yes	No	No	> 12 month	> 32 hours	Single use	80000
Yes	No	No	No	No	> 12 month	< 8 hours	Single use	40000
Yes	Yes	Yes	Yes	No	> 12 month	8-32 hours	Use after c	45000
Yes	No	No	No	No	> 12 month	8-32 hours	Single use	100000
Yes	Yes	Yes	Yes	Yes	> 12 month	8-32 hours	Use after c	45000
Yes	No	No	No	No	> 12 month	8-32 hours	Use after c	40000

No	No	No	Yes	No	> 12 montl	8-32 hours	Use after c	80000
No	No	No	Yes	No	> 12 montl	8-32 hours	Use after c	20000
Yes	Yes	Yes	No	Yes	> 12 montl	> 32 hours	Use after c	10000
Yes	No	No	No	No	> 12 montl	< 8 hours	Single use	50000
No	Yes	No	No	No	> 12 montl	8-32 hours	Use after c	30000
Yes	No	No	No	No	> 12 montl	8-32 hours	Single use	20000
Yes	No	No	No	No	> 12 montl	< 8 hours	Single use	90000
Yes	Yes	No	No	No	> 12 montl	8-32 hours	Single use	50000
No	Yes	No	No	No	9-12 montl	< 8 hours	Use after c	10000
No	No	No	Yes	No	> 12 montl	< 8 hours	Use after c	40000
Yes	No	No	No	No	9-12 montl	< 8 hours	Use after c	40000
Yes	No	No	No	No	> 12 montl	< 8 hours	Single use	50000
Yes	No	No	Yes	No	> 12 montl	8-32 hours	Use withou	70000
Yes	No	No	No	Yes	> 12 montl	< 8 hours	Use after c	50000
No	No	No	Yes	No	9-12 montl	< 8 hours	Use after c	10000
No	No	No	No	Yes	> 12 montl	< 8 hours	Use after c	10000
Yes	No	No	No	No	> 12 montl	< 8 hours	Single use	50000
No	No	No	No	Yes	> 12 montl	8-32 hours	Use after c	30000
Yes	No	No	No	No	> 12 montl	< 8 hours	Use withou	30000
Yes	No	No	No	No	> 12 montl	< 8 hours	Single use	70000
No	No	No	No	Yes	> 12 montl	< 8 hours	Use after c	25000
Yes	Yes	No	No	No	> 12 montl	< 8 hours	Single use	40000
Yes	Yes	No	No	Yes	> 12 montl	< 8 hours	Use withou	70000
No	No	No	No	Yes	> 12 montl	8-32 hours	Use after c	25000
Yes	No	No	Yes	No	9-12 montl	< 8 hours	Use after c	20000
Yes	Yes	No	No	No	> 12 montl	< 8 hours	Single use	90000
Yes	No	No	No	No	> 12 montl	< 8 hours	Single use	100000
Yes	No	No	No	No	> 12 montl	> 32 hours	Single use	20000
Yes	Yes	No	No	No	> 12 montl	< 8 hours	Use withou	50000
Yes	No	No	No	No	> 12 montl	< 8 hours	Single use	60000
Yes	No	No	No	No	> 12 montl	< 8 hours	Single use	80000
Yes	No	No	No	No	> 12 montl	< 8 hours	Use after c	35000
Yes	No	No	No	No	9-12 montl	< 8 hours	Use after c	30000
Yes	No	No	No	No	> 12 montl	> 32 hours	Single use	10000
Yes	No	No	No	No	> 12 montl	8-32 hours	Single use	200000
Yes	No	No	No	No	> 12 montl	8-32 hours	Single use	100000
Yes	No	No	No	No	> 12 montl	8-32 hours	Single use	100000
Yes	No	No	No	Yes	> 12 montl	< 8 hours	Use after c	465000
Yes	No	No	No	Yes	> 12 montl	< 8 hours	Single use	80000
No	No	No	Yes	Yes	> 12 montl	< 8 hours	Use after c	50000
No	No	No	No	Yes	> 12 montl	8-32 hours	Use after c	25000
Yes	No	No	No	No	9-12 montl	< 8 hours	Single use	120000
Yes	Yes	No	No	No	> 12 montl	8-32 hours	Single use	90000
Yes	No	No	No	No	> 12 montl	8-32 hours	Use withou	70000
Yes	No	No	No	No	> 12 montl	< 8 hours	Use after c	12000
Yes	No	No	No	No	> 12 montl	< 8 hours	Use withou	60000
Yes	No	Yes	No	Yes	> 12 montl	8-32 hours	Single use	40000
Yes	No	Yes	No	No	9-12 montl	< 8 hours	Single use	50000
No	Yes	No	No	No	> 12 montl	< 8 hours	Use withou	100000
Yes	Yes	No	No	No	> 12 montl	< 8 hours	Use withou	150000
No	No	No	No	Yes	> 12 montl	8-32 hours	Use after c	150000
Yes	No	No	No	No	> 12 montl	< 8 hours	Single use	10000

Yes	No	No	No	Yes	> 12 montl	< 8 hours	Use after c	10000
Yes	Yes	No	No	Yes	> 12 montl	< 8 hours	Use after c	10000
Yes	No	No	No	No	> 12 montl	< 8 hours	Single use	50000
Yes	No	No	No	No	9-12 montl	< 8 hours	Single use	50000
Yes	No	No	No	No	> 12 montl	8-32 hours	Single use	35000
Yes	No	No	No	No	> 12 montl	< 8 hours	Single use	45000
Yes	No	No	Yes	Yes	9-12 montl	< 8 hours	Use after c	40000
Yes	Yes	No	No	No	9-12 montl	< 8 hours	Single use	300000
Yes	No	No	No	No	> 12 montl	8-32 hours	Single use	15000
Yes	Yes	No	No	Yes	> 12 montl	< 8 hours	Use withou	50000
Yes	Yes	Yes	No	No	> 12 montl	8-32 hours	Single use	60000
Yes	No	No	No	No	> 12 montl	8-32 hours	Single use	70000
Yes	Yes	Yes	Yes	Yes	> 12 montl	8-32 hours	Use after c	20000
Yes	No	No	No	No	> 12 montl	> 32 hours	Use after c	30000
Yes	Yes	No	No	Yes	9-12 montl	< 8 hours	Use after c	40000
Yes	Yes	No	No	No	> 12 montl	< 8 hours	Use after c	150000
Yes	Yes	No	No	No	> 12 montl	< 8 hours	Use after c	40000
Yes	No	No	No	No	9-12 montl	< 8 hours	Use after c	40000
No	No	No	No	Yes	9-12 montl	< 8 hours	Use after c	70000
Yes	No	No	No	Yes	> 12 montl	< 8 hours	Use after c	60000
Yes	No	No	No	No	> 12 montl	> 32 hours	Single use	60000
Yes	No	No	No	No	> 12 montl	< 8 hours	Single use	10000
Yes	No	No	No	No	9-12 montl	> 32 hours	Use after c	100000
Yes	No	No	No	Yes	> 12 montl	8-32 hours	Use after c	80000
Yes	Yes	No	No	No	> 12 montl	< 8 hours	Single use	150000
Yes	No	No	No	No	> 12 montl	< 8 hours	Single use	60000
Yes	No	No	No	Yes	> 12 montl	< 8 hours	Use after c	40000
Yes	No	No	No	Yes	> 12 montl	< 8 hours	Use after c	50000
Yes	No	No	No	Yes	> 12 montl	< 8 hours	Use after c	100000
Yes	No	No	No	No	> 12 montl	< 8 hours	Single use	45000
Yes	Yes	No	No	No	> 12 montl	8-32 hours	Use withou	150000
No	No	No	No	Yes	> 12 montl	8-32 hours	Use after c	70000
Yes	Yes	No	No	No	> 12 montl	8-32 hours	Use after c	50000
Yes	No	No	No	No	9-12 montl	< 8 hours	Single use	50000
Yes	No	No	No	Yes	> 12 montl	< 8 hours	Use after c	20000
Yes	Yes	No	No	Yes	> 12 montl	8-32 hours	Use after c	100000
Yes	Yes	No	No	No	> 12 montl	8-32 hours	Single use	50000
Yes	No	No	No	No	> 12 montl	< 8 hours	Use after c	90000
Yes	No	No	No	No	9-12 montl	8-32 hours	Single use	50000
Yes	No	No	No	No	> 12 montl	8-32 hours	Use after c	50000
Yes	No	No	No	Yes	> 12 montl	< 8 hours	Use after c	25000
Yes	No	No	No	Yes	> 12 montl	< 8 hours	Single use	40000
No	No	No	No	Yes	> 12 montl	< 8 hours	Use after c	40000
Yes	No	No	No	No	> 12 montl	< 8 hours	Single use	60000
Yes	Yes	No	No	Yes	> 12 montl	> 32 hours	Single use	50000
Yes	No	No	No	Yes	> 12 montl	< 8 hours	Single use	40000
Yes	No	No	No	No	> 12 montl	< 8 hours	Single use	80000
Yes	No	No	No	Yes	> 12 montl	< 8 hours	Use after c	500000
Yes	Yes	No	No	Yes	> 12 montl	8-32 hours	Use after c	40000
No	Yes	No	No	No	> 12 montl	8-32 hours	Use after c	20000
No	No	No	Yes	No	> 12 montl	8-32 hours	Single use	35000
Yes	No	No	No	No	> 12 montl	< 8 hours	Single use	30000

Yes	No	No	No	Yes	> 12 month	> 32 hours	Use withou	40000
Yes	Yes	No	No	No	> 12 month	< 8 hours	Use after c	70000
Yes	No	No	No	Yes	> 12 month	< 8 hours	Single use	50000
Yes	No	No	No	No	> 12 month	8-32 hours	Use after c	50000
Yes	Yes	No	No	Yes	> 12 month	< 8 hours	Single use	100000
Yes	Yes	No	No	Yes	> 12 month	< 8 hours	Use after c	40000
Yes	No	No	No	Yes	> 12 month	< 8 hours	Use after c	40000
Yes	Yes	No	No	No	> 12 month	> 32 hours	Single use	30000
Yes	Yes	Yes	Yes	Yes	> 12 month	< 8 hours	Use after c	100000
No	No	No	No	Yes	> 12 month	> 32 hours	Single use	50000
Yes	Yes	No	No	No	> 12 month	> 32 hours	Use after c	11000
Yes	No	No	No	Yes	> 12 month	< 8 hours	Single use	100000
Yes	No	No	No	Yes	> 12 month	< 8 hours	Use after c	50000
Yes	No	No	No	Yes	> 12 month	< 8 hours	Use after c	50000
Yes	No	No	No	No	> 12 month	< 8 hours	Use after c	50000
No	Yes	No	No	No	> 12 month	8-32 hours	Use after c	70000
Yes	No	No	No	Yes	> 12 month	< 8 hours	Single use	50000
Yes	Yes	No	No	No	> 12 month	< 8 hours	Single use	50000
Yes	Yes	No	No	No	> 12 month	< 8 hours	Single use	50000
Yes	No	No	No	Yes	9-12 month	< 8 hours	Use after c	60000
No	No	No	No	Yes	> 12 month	< 8 hours	Use after c	200000
No	Yes	No	No	No	> 12 month	8-32 hours	Use after c	300000
Yes	No	No	No	No	> 12 month	< 8 hours	Use after c	1000000
Yes	No	No	No	No	> 12 month	< 8 hours	Single use	100000
Yes	No	No	No	No	> 12 month	< 8 hours	Single use	30000
Yes	No	No	No	No	9-12 month	< 8 hours	Single use	5000
Yes	No	No	Yes	No	> 12 month	8-32 hours	Single use	40000
Yes	No	No	No	Yes	> 12 month	< 8 hours	Single use	50000
Yes	No	No	No	No	> 12 month	< 8 hours	Single use	100000
Yes	Yes	No	No	No	> 12 month	< 8 hours	Use after c	56000
Yes	No	No	No	Yes	> 12 month	8-32 hours	Use after c	40000
Yes	No	No	No	No	> 12 month	< 8 hours	Single use	200000
Yes	No	No	No	No	9-12 month	< 8 hours	Use after c	50000
Yes	No	No	No	No	> 12 month	< 8 hours	Single use	60000
Yes	Yes	No	No	No	> 12 month	8-32 hours	Use after c	100000
Yes	Yes	No	No	No	> 12 month	< 8 hours	Use after c	70000
No	Yes	No	No	Yes	9-12 month	< 8 hours	Use after c	100000
No	No	No	No	Yes	> 12 month	< 8 hours	Use after c	80000
Yes	No	No	No	Yes	> 12 month	< 8 hours	Use after c	35000
Yes	No	No	No	No	> 12 month	< 8 hours	Single use	40000
Yes	No	No	No	No	9-12 month	< 8 hours	Single use	50000
Yes	No	No	No	No	> 12 month	< 8 hours	Single use	40000
Yes	No	No	No	No	9-12 month	< 8 hours	Use withou	90000
Yes	No	No	No	No	> 12 month	< 8 hours	Use after c	50000
Yes	No	No	No	Yes	> 12 month	8-32 hours	Use after c	80000
Yes	No	No	No	No	> 12 month	< 8 hours	Single use	40000
Yes	Yes	No	No	No	9-12 month	< 8 hours	Use after c	100000
Yes	No	No	No	No	> 12 month	< 8 hours	Use after c	50000
Yes	No	No	No	Yes	> 12 month	< 8 hours	Use after c	100000
Yes	No	No	No	Yes	> 12 month	< 8 hours	Use after c	30000
Yes	No	No	No	Yes	> 12 month	8-32 hours	Single use	40000
Yes	Yes	No	No	Yes	> 12 month	8-32 hours	Use after c	15000

Yes	No	No	No	No	> 12 montl	< 8 hours	Single use	60000
Yes	No	Yes	No	No	> 12 montl	< 8 hours	Single use	40000
No	No	No	No	Yes	> 12 montl	> 32 hours	Use after c	35000
Yes	No	No	No	Yes	> 12 montl	> 32 hours	Use after c	30000
Yes	No	Yes	No	No	> 12 montl	> 32 hours	Single use	25000
Yes	No	Yes	No	Yes	9-12 montl	> 32 hours	Use after c	28000
Yes	No	No	No	No	> 12 montl	< 8 hours	Use after c	20000
Yes	No	No	No	No	> 12 montl	< 8 hours	Single use	30000
Yes	No	No	No	Yes	> 12 montl	< 8 hours	Use after c	30000
Yes	No	No	No	No	> 12 montl	> 32 hours	Single use	22000
Yes	No	No	No	No	9-12 montl	< 8 hours	Single use	10000
No	No	Yes	No	No	> 12 montl	8-32 hours	Single use	30000
Yes	Yes	No	No	Yes	> 12 montl	8-32 hours	Single use	40000
Yes	No	No	No	No	> 12 montl	< 8 hours	Single use	500000
Yes	No	No	No	No	> 12 montl	< 8 hours	Use after c	60000
Yes	No	No	No	No	> 12 montl	< 8 hours	Use withou	20000
Yes	No	No	No	No	> 12 montl	> 32 hours	Single use	25000
Yes	No	No	No	Yes	> 12 montl	8-32 hours	Single use	40000
Yes	Yes	No	No	No	> 12 montl	< 8 hours	Single use	250000
Yes	No	No	No	Yes	> 12 montl	< 8 hours	Single use	80000
No	No	No	Yes	No	9-12 montl	< 8 hours	Use after c	70000
Yes	No	No	No	No	9-12 montl	< 8 hours	Single use	40000
Yes	Yes	No	No	No	> 12 montl	8-32 hours	Use after c	25000
Yes	No	No	No	No	> 12 montl	< 8 hours	Single use	50000
Yes	No	No	No	No	> 12 montl	8-32 hours	Single use	450000
Yes	No	No	No	No	9-12 montl	< 8 hours	Use after c	25000
No	No	No	No	Yes	> 12 montl	< 8 hours	Use after c	20000
No	No	No	No	Yes	> 12 montl	< 8 hours	Use after c	80000
Yes	No	No	No	No	> 12 montl	> 32 hours	Single use	50000
Yes	Yes	No	No	No	> 12 montl	8-32 hours	Use withou	8000
Yes	No	No	No	No	> 12 montl	< 8 hours	Single use	25000
Yes	No	No	No	No	> 12 montl	8-32 hours	Single use	20000
Yes	No	No	No	Yes	> 12 montl	< 8 hours	Use after c	60000
Yes	No	No	No	No	> 12 montl	< 8 hours	Use after c	35000
Yes	No	No	No	Yes	> 12 montl	< 8 hours	Single use	40000
Yes	No	No	No	No	> 12 montl	< 8 hours	Single use	50000
Yes	No	No	No	No	> 12 montl	< 8 hours	Use after c	80000
Yes	No	No	No	No	> 12 montl	8-32 hours	Use after c	100000
Yes	No	No	No	No	9-12 montl	< 8 hours	Single use	24000
No	Yes	No	No	No	> 12 montl	> 32 hours	Single use	100000
Yes	No	No	No	No	> 12 montl	< 8 hours	Single use	98000
No	No	No	No	Yes	> 12 montl	< 8 hours	Use after c	40000
Yes	No	No	No	No	> 12 montl	< 8 hours	Use after c	60000
Yes	No	No	No	No	> 12 montl	< 8 hours	Single use	80000
Yes	No	No	No	Yes	> 12 montl	< 8 hours	Single use	50000
Yes	No	No	No	Yes	> 12 montl	8-32 hours	Use after c	50000
Yes	No	No	No	No	> 12 montl	< 8 hours	Single use	200000
Yes	No	No	No	No	> 12 montl	8-32 hours	Use after c	50000
Yes	No	No	No	No	> 12 montl	8-32 hours	Use after c	80000
Yes	No	No	No	No	> 12 montl	< 8 hours	Single use	50000
Yes	No	No	No	Yes	> 12 montl	< 8 hours	Single use	100000
Yes	Yes	No	No	No	> 12 montl	< 8 hours	Use after c	50000

Yes	No	No	No	No	> 12 montl	8-32 hours	Single use	50000
Yes	No	No	No	No	> 12 montl	< 8 hours	Single use	40000
Yes	Yes	No	No	Yes	> 12 montl	8-32 hours	Use after c	78000
No	No	No	No	Yes	> 12 montl	< 8 hours	Use after c	60000
Yes	No	Yes	No	Yes	> 12 montl	< 8 hours	Single use	30000
Yes	No	No	No	Yes	> 12 montl	8-32 hours	Single use	20000
Yes	No	No	No	No	> 12 montl	8-32 hours	Use after c	80000
Yes	No	No	No	Yes	> 12 montl	8-32 hours	Single use	100000
Yes	No	No	No	No	> 12 montl	< 8 hours	Use withou	200000
No	No	No	Yes	No	> 12 montl	< 8 hours	Use after c	40000
Yes	No	Yes	No	No	> 12 montl	< 8 hours	Use after c	60000
Yes	Yes	Yes	No	Yes	> 12 montl	< 8 hours	Single use	40000
Yes	No	Yes	No	No	> 12 montl	> 32 hours	Single use	45000
Yes	No	No	No	No	> 12 montl	> 32 hours	Use after c	34000
Yes	No	No	No	Yes	> 12 montl	> 32 hours	Use after c	30000
Yes	No	No	No	No	9-12 montl	< 8 hours	Single use	35000
Yes	No	No	No	No	9-12 montl	< 8 hours	Single use	25000
Yes	No	No	No	No	> 12 montl	< 8 hours	Single use	40000
Yes	No	No	No	No	> 12 montl	8-32 hours	Single use	40000
Yes	No	No	No	No	> 12 montl	> 32 hours	Single use	100000
Yes	No	No	No	Yes	> 12 montl	8-32 hours	Use after c	300000
Yes	No	No	No	No	> 12 montl	< 8 hours	Use withou	30000
Yes	No	No	No	No	> 12 montl	> 32 hours	Single use	80000
Yes	No	No	No	No	> 12 montl	< 8 hours	Single use	30000
Yes	No	No	No	No	> 12 montl	< 8 hours	Single use	20000
Yes	No	No	No	No	> 12 montl	< 8 hours	Use after c	50000
No	No	No	No	Yes	> 12 montl	< 8 hours	Use after c	5000
Yes	No	No	No	No	> 12 montl	< 8 hours	Single use	30000
No	No	No	No	Yes	> 12 montl	8-32 hours	Use after c	5000
Yes	No	No	No	Yes	> 12 montl	8-32 hours	Single use	35000
Yes	No	No	No	No	9-12 montl	< 8 hours	Single use	50000
Yes	Yes	No	No	No	> 12 montl	8-32 hours	Use withou	40000
Yes	No	No	No	No	> 12 montl	> 32 hours	Single use	40000
No	No	No	No	Yes	> 12 montl	< 8 hours	Use withou	40000
No	No	No	Yes	No	> 12 montl	< 8 hours	Use after c	13000
Yes	No	No	No	Yes	> 12 montl	< 8 hours	Use withou	20000
Yes	No	No	No	Yes	> 12 montl	> 32 hours	Single use	40000
No	No	No	No	Yes	9-12 montl	< 8 hours	Single use	15000
Yes	No	No	No	Yes	> 12 montl	8-32 hours	Use after c	30000
Yes	No	No	No	No	9-12 montl	> 32 hours	Single use	20000
Yes	Yes	No	No	Yes	> 12 montl	> 32 hours	Use after c	30000
Yes	No	No	No	No	> 12 montl	8-32 hours	Single use	30000
Yes	No	No	No	No	> 12 montl	8-32 hours	Use after c	30000
No	No	No	No	Yes	> 12 montl	> 32 hours	Use after c	30000
No	No	No	No	Yes	> 12 montl	< 8 hours	Use after c	30000
No	No	No	No	Yes	> 12 montl	< 8 hours	Use after c	30000
Yes	No	No	No	Yes	> 12 montl	> 32 hours	Use after c	50000
Yes	Yes	No	No	Yes	> 12 montl	< 8 hours	Use after c	100000
Yes	No	No	No	Yes	> 12 montl	< 8 hours	Use withou	100000
Yes	No	No	No	No	> 12 montl	< 8 hours	Single use	15000
Yes	No	No	No	No	> 12 montl	> 32 hours	Single use	60000
Yes	No	No	No	Yes	> 12 montl	> 32 hours	Single use	50000

Yes	Yes	No	No	No	> 12 month	> 32 hours	Use after c	100000
Yes	No	No	No	Yes	> 12 month	8-32 hours	Use after c	20000
Yes	No	No	No	Yes	> 12 month	< 8 hours	Use after c	20000
Yes	No	No	No	Yes	> 12 month	> 32 hours	Use after c	100000
Yes	No	No	No	No	> 12 month	< 8 hours	Single use	25000
Yes	Yes	No	No	No	> 12 month	< 8 hours	Single use	30000
Yes	No	No	No	No	> 12 month	> 32 hours	Use withou	100000
Yes	No	No	No	No	> 12 month	> 32 hours	Single use	40000
Yes	No	No	No	No	> 12 month	< 8 hours	Single use	15000
Yes	No	No	No	No	> 12 month	8-32 hours	Single use	150000
Yes	No	No	No	No	> 12 month	> 32 hours	Single use	150000
Yes	No	No	No	No	> 12 month	< 8 hours	Single use	150000
Yes	No	No	No	No	> 12 month	8-32 hours	Single use	500000
Yes	No	No	No	No	> 12 month	< 8 hours	Use after c	15000
No	No	Yes	No	Yes	> 12 month	8-32 hours	Use after c	35000
Yes	No	No	No	Yes	> 12 month	< 8 hours	Single use	60000
Yes	No	No	No	No	> 12 month	> 32 hours	Single use	35000
Yes	No	No	No	Yes	> 12 month	< 8 hours	Use after c	40000
Yes	No	No	No	No	> 12 month	< 8 hours	Single use	50000
Yes	No	No	No	No	9-12 month	> 32 hours	Single use	20000
Yes	No	No	Yes	No	> 12 month	< 8 hours	Use after c	50000
Yes	No	No	No	Yes	> 12 month	< 8 hours	Use after c	30000
Yes	Yes	No	No	No	> 12 month	8-32 hours	Single use	30000
Yes	No	No	No	Yes	> 12 month	< 8 hours	Use after c	40000
Yes	No	No	No	No	> 12 month	< 8 hours	Use after c	24000
Yes	No	No	No	No	> 12 month	< 8 hours	Use after c	30000
Yes	Yes	No	No	No	> 12 month	< 8 hours	Single use	175000
Yes	No	No	No	No	> 12 month	< 8 hours	Use after c	20000
Yes	No	No	No	No	> 12 month	< 8 hours	Single use	8000
No	No	No	No	Yes	> 12 month	8-32 hours	Use after c	15000
Yes	No	No	No	No	> 12 month	< 8 hours	Single use	20000
Yes	No	No	No	Yes	> 12 month	< 8 hours	Single use	45000
No	No	No	Yes	No	9-12 month	< 8 hours	Use after c	210000
No	No	No	No	Yes	> 12 month	< 8 hours	Single use	250000
Yes	No	No	No	Yes	> 12 month	> 32 hours	Use after c	50000
Yes	No	No	No	No	> 12 month	< 8 hours	Use after c	60000
Yes	No	No	No	No	> 12 month	> 32 hours	Single use	50000
Yes	No	No	No	No	> 12 month	< 8 hours	Use after c	50000
Yes	No	No	No	No	> 12 month	< 8 hours	Single use	30000
Yes	Yes	No	No	Yes	9-12 month	< 8 hours	Use after c	110000
Yes	No	No	No	No	> 12 month	8-32 hours	Single use	50000
Yes	No	No	No	No	> 12 month	< 8 hours	Single use	100000
Yes	Yes	No	No	Yes	> 12 month	< 8 hours	Use after c	50000
Yes	No	No	No	Yes	> 12 month	8-32 hours	Single use	10000
No	No	No	Yes	No	> 12 month	> 32 hours	Single use	100000
Yes	Yes	No	No	No	> 12 month	8-32 hours	Single use	30000
Yes	Yes	Yes	No	No	> 12 month	8-32 hours	Use after c	60000
Yes	Yes	No	No	Yes	9-12 month	< 8 hours	Single use	100000
No	No	No	Yes	No	> 12 month	8-32 hours	Use after c	100000
Yes	Yes	Yes	No	Yes	> 12 month	8-32 hours	Use after c	50000
Yes	Yes	No	Yes	No	> 12 month	< 8 hours	Use after c	100000
Yes	No	No	No	No	> 12 month	8-32 hours	Single use	30000

Yes	No	No	No	No	> 12 montl	8-32 hours	Single use	2000
Yes	Yes	No	No	Yes	> 12 montl	< 8 hours	Use after c	100000
Yes	No	No	No	No	> 12 montl	< 8 hours	Single use	60000
Yes	Yes	No	No	No	> 12 montl	< 8 hours	Use after c	60000
Yes	No	No	No	No	> 12 montl	8-32 hours	Single use	50000
Yes	No	No	No	Yes	9-12 montl	> 32 hours	Use after c	60000
Yes	No	No	No	No	> 12 montl	< 8 hours	Single use	50000
Yes	No	No	No	No	9-12 montl	< 8 hours	Use after c	240000
Yes	No	No	No	No	> 12 montl	> 32 hours	Use withou	40000
Yes	No	No	Yes	Yes	> 12 montl	< 8 hours	Single use	40000
Yes	No	Yes	No	No	> 12 montl	8-32 hours	Use withou	35000
Yes	No	Yes	No	No	> 12 montl	8-32 hours	Use after c	35000
Yes	No	Yes	No	No	> 12 montl	8-32 hours	Use withou	30000
Yes	No	No	No	Yes	> 12 montl	< 8 hours	Use after c	100000
Yes	No	No	No	Yes	> 12 montl	8-32 hours	Use after c	35000
No	No	No	No	Yes	> 12 montl	> 32 hours	Single use	3500
Yes	No	No	No	No	9-12 montl	< 8 hours	Use after c	50000
Yes	No	No	No	No	> 12 montl	> 32 hours	Use after c	20000
Yes	No	No	No	No	> 12 montl	8-32 hours	Use after c	50000
Yes	Yes	No	No	No	> 12 montl	< 8 hours	Use after c	60000
Yes	No	No	No	No	> 12 montl	< 8 hours	Use after c	100000
Yes	No	No	No	No	> 12 montl	8-32 hours	Use after c	100000
No	No	No	No	Yes	> 12 montl	> 32 hours	Use after c	15000
Yes	Yes	No	Yes	No	> 12 montl	> 32 hours	Single use	20000
Yes	No	No	No	No	9-12 montl	8-32 hours	Single use	20000
Yes	No	No	No	No	> 12 montl	8-32 hours	Single use	90000
Yes	No	No	No	No	9-12 montl	< 8 hours	Use after c	30000
Yes	No	No	No	No	> 12 montl	8-32 hours	Single use	70000
Yes	No	No	No	No	> 12 montl	< 8 hours	Use withou	8000
Yes	No	No	No	No	9-12 montl	< 8 hours	Single use	65000
Yes	No	No	No	Yes	9-12 montl	< 8 hours	Single use	70000
Yes	No	No	Yes	Yes	> 12 montl	< 8 hours	Use after c	50000
Yes	No	No	No	No	> 12 montl	< 8 hours	Use after c	40000
Yes	No	No	No	Yes	> 12 montl	8-32 hours	Single use	40000
Yes	No	No	No	Yes	> 12 montl	8-32 hours	Single use	23000
Yes	No	No	Yes	No	> 12 montl	< 8 hours	Single use	25000
Yes	No	No	No	No	> 12 montl	> 32 hours	Single use	80000
Yes	No	No	No	No	> 12 montl	> 32 hours	Single use	60000
No	No	No	No	Yes	> 12 montl	> 32 hours	Use after c	60000
Yes	No	No	Yes	Yes	> 12 montl	< 8 hours	Use after c	30000
Yes	No	No	No	No	9-12 montl	8-32 hours	Single use	25000
Yes	No	No	No	No	> 12 montl	8-32 hours	Use after c	50000
Yes	No	No	No	No	> 12 montl	< 8 hours	Use after c	100000
Yes	No	No	No	No	> 12 montl	8-32 hours	Single use	20000
Yes	No	No	No	Yes	> 12 montl	8-32 hours	Use after c	35000
Yes	No	No	No	Yes	> 12 montl	8-32 hours	Single use	50000
No	No	No	Yes	Yes	9-12 montl	8-32 hours	Single use	50000
Yes	No	No	No	No	> 12 montl	> 32 hours	Use withou	89000
No	Yes	No	No	No	> 12 montl	8-32 hours	Single use	40000
Yes	No	No	No	No	> 12 montl	> 32 hours	Use after c	60000
No	No	No	No	Yes	> 12 montl	> 32 hours	Use after c	40000
Yes	No	No	No	No	> 12 montl	8-32 hours	Single use	30000

Yes	No	No	No	No	> 12 montl	> 32 hours	Single use	60000
Yes	No	No	No	No	> 12 montl	> 32 hours	Single use	50000
Yes	Yes	Yes	No	No	> 12 montl	> 32 hours	Single use	100000
Yes	Yes	Yes	Yes	Yes	> 12 montl	> 32 hours	Single use	100000
Yes	No	No	No	No	> 12 montl	< 8 hours	Single use	100000
Yes	No	No	No	No	> 12 montl	8-32 hours	Use after c	50000
Yes	No	No	No	Yes	> 12 montl	8-32 hours	Use after c	40000
Yes	No	No	No	No	9-12 montl	< 8 hours	Single use	25000
Yes	Yes	No	No	No	> 12 montl	8-32 hours	Single use	40000
Yes	No	No	No	No	> 12 montl	8-32 hours	Single use	50000
Yes	No	No	No	No	> 12 montl	8-32 hours	Single use	60000
Yes	No	No	No	No	9-12 montl	8-32 hours	Use withou	70000
Yes	No	No	No	Yes	> 12 montl	< 8 hours	Single use	40000
No	No	No	Yes	No	> 12 montl	< 8 hours	Use after c	22000
Yes	No	No	No	No	> 12 montl	> 32 hours	Single use	80000
Yes	Yes	No	No	Yes	> 12 montl	> 32 hours	Use after c	40000
Yes	No	No	No	No	> 12 montl	< 8 hours	Single use	50000
Yes	No	No	No	Yes	9-12 montl	< 8 hours	Single use	70000
No	No	No	No	Yes	> 12 montl	< 8 hours	Use after c	50000
No	Yes	No	No	No	> 12 montl	8-32 hours	Single use	20000
No	Yes	No	No	No	> 12 montl	8-32 hours	Use after c	50000
No	No	No	No	Yes	> 12 montl	< 8 hours	Use after c	30000
No	No	No	No	Yes	9-12 montl	< 8 hours	Use after c	100000
No	No	No	No	Yes	9-12 montl	< 8 hours	Single use	45000
Yes	No	No	No	No	> 12 montl	> 32 hours	Single use	90000
No	No	No	No	Yes	> 12 montl	8-32 hours	Use after c	20000
Yes	No	No	No	Yes	9-12 montl	< 8 hours	Single use	20000
Yes	No	No	No	Yes	> 12 montl	8-32 hours	Single use	300000
Yes	No	No	No	No	> 12 montl	8-32 hours	Use after c	40000
Yes	No	No	Yes	Yes	> 12 montl	8-32 hours	Single use	40000
Yes	No	No	No	Yes	> 12 montl	8-32 hours	Single use	30000
Yes	No	No	No	No	> 12 montl	> 32 hours	Single use	80000
Yes	No	No	No	No	> 12 montl	< 8 hours	Single use	25000
Yes	No	No	No	No	> 12 montl	8-32 hours	Use after c	25000
Yes	No	No	No	No	> 12 montl	< 8 hours	Single use	40000
No	No	No	Yes	No	> 12 montl	> 32 hours	Use after c	20000
Yes	No	No	No	No	> 12 montl	> 32 hours	Single use	50000
Yes	No	No	No	No	> 12 montl	> 32 hours	Single use	8000
Yes	No	No	No	No	> 12 montl	8-32 hours	Single use	60000
Yes	No	No	No	No	> 12 montl	< 8 hours	Single use	30000
Yes	No	No	No	No	> 12 montl	8-32 hours	Single use	30000
Yes	No	No	No	No	> 12 montl	< 8 hours	Single use	50000
Yes	Yes	No	No	No	> 12 montl	8-32 hours	Use after c	75000
Yes	Yes	No	No	Yes	> 12 montl	< 8 hours	Use after c	40000
Yes	Yes	No	Yes	Yes	9-12 montl	< 8 hours	Use after c	40000
Yes	No	No	No	Yes	> 12 montl	< 8 hours	Use withou	45000
No	No	No	Yes	No	9-12 montl	8-32 hours	Use after c	34000
Yes	No	No	No	No	> 12 montl	8-32 hours	Use after c	70000
Yes	Yes	No	No	Yes	> 12 montl	> 32 hours	Use after c	50000
Yes	No	No	No	Yes	> 12 montl	> 32 hours	Use after c	35000
Yes	No	No	Yes	No	> 12 montl	< 8 hours	Single use	90000
Yes	No	No	No	No	> 12 montl	8-32 hours	Single use	200000

Yes	No	No	No	No	> 12 montl	> 32 hours	Single use	150000
Yes	No	No	No	No	> 12 montl	8-32 hours	Single use	100000
Yes	No	No	No	Yes	> 12 montl	> 32 hours	Single use	30000
Yes	No	No	No	Yes	9-12 montl	> 32 hours	Single use	30000
Yes	No	No	No	No	> 12 montl	> 32 hours	Single use	25000
Yes	No	No	No	No	> 12 montl	8-32 hours	Single use	100000
Yes	No	No	No	No	> 12 montl	< 8 hours	Single use	20000
Yes	No	No	Yes	Yes	> 12 montl	8-32 hours	Single use	15000
Yes	No	No	No	Yes	> 12 montl	< 8 hours	Use withou	15000
Yes	No	No	No	Yes	> 12 montl	< 8 hours	Use after c	12000
Yes	No	No	No	Yes	> 12 montl	< 8 hours	Use after c	16000
Yes	No	No	No	No	> 12 montl	< 8 hours	Single use	10000
Yes	No	No	No	Yes	> 12 montl	< 8 hours	Single use	20000
Yes	No	No	No	No	> 12 montl	< 8 hours	Single use	40000
Yes	No	No	No	No	> 12 montl	< 8 hours	Single use	30000
Yes	No	No	No	Yes	> 12 montl	< 8 hours	Single use	10000
Yes	No	No	No	Yes	> 12 montl	< 8 hours	Single use	10000
Yes	No	No	No	Yes	> 12 montl	< 8 hours	Single use	15000
Yes	No	No	No	Yes	> 12 montl	< 8 hours	Single use	10000
Yes	No	No	No	No	> 12 montl	> 32 hours	Single use	15000
Yes	No	No	No	No	> 12 montl	< 8 hours	Single use	10000
Yes	No	No	No	No	> 12 montl	> 32 hours	Single use	10000
No	No	No	No	Yes	> 12 montl	< 8 hours	Use after c	10000
Yes	No	No	No	No	> 12 montl	> 32 hours	Use after c	15000
Yes	No	No	No	No	> 12 montl	< 8 hours	Use after c	25000
Yes	No	No	No	No	9-12 montl	< 8 hours	Single use	30000
Yes	No	No	Yes	No	> 12 montl	< 8 hours	Single use	40000
Yes	No	No	No	No	> 12 montl	< 8 hours	Single use	20000
Yes	No	No	No	No	> 12 montl	> 32 hours	Single use	30000
Yes	No	No	No	No	> 12 montl	< 8 hours	Use withou	30000
No	No	No	No	Yes	9-12 montl	8-32 hours	Use after c	50000
Yes	No	No	No	Yes	9-12 montl	8-32 hours	Use after c	40000
Yes	No	No	No	Yes	> 12 montl	> 32 hours	Use after c	22000
Yes	No	No	No	Yes	> 12 montl	> 32 hours	Use after c	25000
Yes	No	No	No	Yes	> 12 montl	> 32 hours	Use after c	20000
No	No	No	No	Yes	> 12 montl	8-32 hours	Use after c	15000
Yes	Yes	No	Yes	No	> 12 montl	< 8 hours	Use after c	40000
Yes	No	No	No	Yes	> 12 montl	> 32 hours	Use after c	16000
Yes	No	No	No	Yes	> 12 montl	> 32 hours	Use after c	25000
No	No	No	No	Yes	> 12 montl	> 32 hours	Use after c	18000
Yes	Yes	No	No	No	> 12 montl	8-32 hours	Use after c	100000
Yes	Yes	No	No	No	> 12 montl	> 32 hours	Use after c	100000
Yes	Yes	No	No	No	> 12 montl	< 8 hours	Use after c	75000
Yes	No	No	No	No	> 12 montl	> 32 hours	Use after c	40000
Yes	No	No	No	No	9-12 montl	8-32 hours	Use withou	30000
Yes	No	No	No	Yes	> 12 montl	8-32 hours	Use after c	100000
Yes	No	No	No	No	> 12 montl	< 8 hours	Single use	50000
No	No	No	No	Yes	> 12 montl	> 32 hours	Use after c	100000
No	No	No	No	Yes	> 12 montl	8-32 hours	Use after c	20000
Yes	Yes	No	No	No	> 12 montl	< 8 hours	Use after c	8000
Yes	No	No	No	No	> 12 montl	< 8 hours	Single use	40000
Yes	No	No	No	No	9-12 montl	8-32 hours	Single use	45000

Yes	No	No	No	Yes	> 12 month	8-32 hours	Single use	40000
Yes	No	No	No	Yes	> 12 month	> 32 hours	Use after c	100000
Yes	No	No	No	No	> 12 month	8-32 hours	Use withou	100000
Yes	No	No	No	No	> 12 month	> 32 hours	Single use	150000
Yes	No	No	No	No	> 12 month	8-32 hours	Use after c	150000
Yes	No	No	No	No	> 12 month	8-32 hours	Use withou	20000
Yes	No	No	No	No	> 12 month	8-32 hours	Use after c	150000
Yes	No	No	No	Yes	> 12 month	< 8 hours	Single use	60000
Yes	No	No	No	No	> 12 month	> 32 hours	Single use	200000
Yes	No	No	No	No	> 12 month	> 32 hours	Single use	80000
No	No	No	No	Yes	> 12 month	> 32 hours	Single use	34000
Yes	No	No	No	Yes	> 12 month	> 32 hours	Use after c	18000
Yes	No	No	No	Yes	> 12 month	> 32 hours	Single use	25000
Yes	Yes	No	Yes	Yes	> 12 month	8-32 hours	Single use	60000
Yes	No	No	No	Yes	> 12 month	< 8 hours	Single use	60000
Yes	No	No	No	No	> 12 month	< 8 hours	Single use	20000
Yes	No	No	No	No	> 12 month	< 8 hours	Use withou	200000
Yes	No	No	No	No	> 12 month	< 8 hours	Use withou	100000
Yes	No	No	No	No	> 12 month	8-32 hours	Single use	100000
Yes	No	No	No	No	> 12 month	> 32 hours	Single use	100000
Yes	No	No	No	No	> 12 month	< 8 hours	Single use	100000
Yes	No	No	No	No	> 12 month	> 32 hours	Single use	100000
Yes	No	No	No	No	> 12 month	< 8 hours	Use after c	10000
Yes	No	No	No	No	> 12 month	< 8 hours	Single use	30000
Yes	No	No	No	Yes	> 12 month	> 32 hours	Single use	30000
No	No	No	No	Yes	9-12 month	< 8 hours	Use after c	109384
Yes	No	No	No	No	> 12 month	< 8 hours	Single use	100000
Yes	No	No	No	Yes	9-12 month	> 32 hours	Use after c	25000
No	No	No	No	Yes	> 12 month	> 32 hours	Single use	27000
Yes	Yes	No	No	No	> 12 month	> 32 hours	Use after c	30000
Yes	No	No	No	Yes	> 12 month	> 32 hours	Use after c	25000
Yes	No	No	No	No	> 12 month	< 8 hours	Single use	100000
Yes	No	No	No	Yes	> 12 month	< 8 hours	Single use	100000
Yes	No	No	No	No	> 12 month	< 8 hours	Single use	100000
Yes	No	No	No	Yes	> 12 month	> 32 hours	Single use	25000
Yes	No	No	No	Yes	> 12 month	8-32 hours	Use after c	100000
Yes	No	No	No	Yes	> 12 month	> 32 hours	Single use	20000
Yes	No	No	No	Yes	> 12 month	8-32 hours	Use after c	100000
No	No	No	No	Yes	> 12 month	> 32 hours	Single use	22000
Yes	No	No	No	Yes	9-12 month	> 32 hours	Use after c	25000
Yes	No	No	No	Yes	> 12 month	> 32 hours	Use after c	35000
No	No	No	No	Yes	9-12 month	> 32 hours	Use after c	26000
Yes	No	No	No	No	> 12 month	> 32 hours	Use after c	29000
Yes	No	No	No	Yes	> 12 month	> 32 hours	Use after c	28000
No	No	No	No	Yes	> 12 month	> 32 hours	Use after c	27000
Yes	No	No	No	Yes	> 12 month	> 32 hours	Use after c	50000
No	No	No	No	Yes	> 12 month	< 8 hours	Use after c	50000
Yes	No	No	No	Yes	> 12 month	> 32 hours	Use after c	70000
Yes	No	No	No	No	> 12 month	< 8 hours	Use after c	60000
Yes	No	No	No	No	> 12 month	< 8 hours	Use after c	20000
Yes	No	No	No	No	> 12 month	< 8 hours	Use withou	100000
Yes	No	No	No	No	> 12 month	> 32 hours	Single use	100000

Yes	No	No	No	No	> 12 montł	> 32 hours	Single use	10000
Yes	No	No	No	Yes	> 12 montł	8-32 hours	Use after c	30000
Yes	No	No	No	No	> 12 montł	< 8 hours	Single use	50000
Yes	No	No	No	No	> 12 montł	> 32 hours	Single use	50000
Yes	No	No	No	No	> 12 montł	> 32 hours	Single use	10000
Yes	No	No	No	No	> 12 montł	< 8 hours	Single use	100000
Yes	No	No	No	No	> 12 montł	8-32 hours	Single use	100000
Yes	No	No	No	No	> 12 montł	< 8 hours	Single use	100000
Yes	No	No	Yes	Yes	9-12 montł	> 32 hours	Single use	15000
Yes	No	No	No	Yes	> 12 montł	8-32 hours	Use after c	30000
Yes	Yes	No	No	No	> 12 montł	8-32 hours	Use after c	60000
Yes	No	No	No	No	> 12 montł	8-32 hours	Single use	50000
No	No	No	No	Yes	> 12 montł	8-32 hours	Use after c	45000
Yes	No	No	No	No	9-12 montł	8-32 hours	Single use	100000
Yes	No	No	No	Yes	> 12 montł	< 8 hours	Use after c	30000
Yes	No	No	No	No	> 12 montł	> 32 hours	Single use	100000
Yes	Yes	No	No	No	> 12 montł	8-32 hours	Single use	60000
Yes	No	No	No	No	> 12 montł	> 32 hours	Single use	17000
Yes	Yes	Yes	Yes	Yes	> 12 montł	8-32 hours	Use after c	25000
Yes	No	No	No	No	> 12 montł	8-32 hours	Single use	50000
Yes	No	No	No	Yes	9-12 montł	< 8 hours	Use after c	20000
Yes	No	No	No	No	> 12 montł	8-32 hours	Single use	30000
No	No	No	Yes	No	> 12 montł	< 8 hours	Use after c	20000
No	No	No	Yes	No	> 12 montł	< 8 hours	Single use	20000
No	Yes	No	No	No	> 12 montł	> 32 hours	Use after c	30000
No	No	No	Yes	No	9-12 montł	8-32 hours	Single use	30000
Yes	No	No	No	No	> 12 montł	< 8 hours	Single use	20000
Yes	No	No	No	Yes	> 12 montł	8-32 hours	Use after c	50000
Yes	No	No	No	Yes	> 12 montł	< 8 hours	Use after c	50000
Yes	No	No	No	Yes	> 12 montł	8-32 hours	Use after c	50000
Yes	No	No	No	Yes	> 12 montł	8-32 hours	Single use	100000
Yes	No	No	No	Yes	> 12 montł	8-32 hours	Use after c	50000
Yes	No	No	No	Yes	> 12 montł	> 32 hours	Single use	100000
Yes	No	No	No	Yes	> 12 montł	< 8 hours	Use after c	50000
Yes	Yes	Yes	No	No	> 12 montł	> 32 hours	Single use	100000
Yes	No	No	No	Yes	> 12 montł	8-32 hours	Use after c	100000
Yes	No	No	No	No	> 12 montł	< 8 hours	Single use	50000
Yes	No	No	No	No	> 12 montł	< 8 hours	Single use	50000
Yes	No	No	No	No	> 12 montł	< 8 hours	Single use	100000
Yes	No	No	No	No	> 12 montł	< 8 hours	Single use	100000
Yes	No	No	No	No	> 12 montł	< 8 hours	Single use	100000
Yes	No	No	No	No	> 12 montł	> 32 hours	Single use	100000
Yes	No	No	No	No	> 12 montł	< 8 hours	Single use	100000
Yes	No	No	No	No	> 12 montł	< 8 hours	Single use	100000
Yes	No	No	No	Yes	> 12 montł	< 8 hours	Single use	100000
Yes	No	No	No	No	> 12 montł	> 32 hours	Single use	100000
Yes	No	No	No	Yes	> 12 montł	> 32 hours	Use after c	25000
Yes	No	No	No	No	> 12 montł	> 32 hours	Single use	24000
No	No	No	No	Yes	> 12 montł	> 32 hours	Use after c	15000
No	No	No	No	Yes	> 12 montł	> 32 hours	Use after c	35000
No	No	No	No	Yes	> 12 montł	> 32 hours	Use after c	22000
No	No	No	No	Yes	9-12 montł	> 32 hours	Use after c	25000

Yes	No	No	No	No	> 12 montl	> 32 hours	Single use	20000
Yes	No	No	No	No	> 12 montl	> 32 hours	Single use	18000
No	No	No	No	Yes	> 12 montl	< 8 hours	Single use	10000
Yes	No	No	No	No	> 12 montl	> 32 hours	Single use	25000
No	No	No	No	Yes	9-12 montl	> 32 hours	Use after c	16000
Yes	No	No	No	No	> 12 montl	8-32 hours	Use after c	24000
Yes	No	No	No	Yes	> 12 montl	> 32 hours	Use after c	35000
Yes	No	No	No	Yes	9-12 montl	> 32 hours	Use after c	30000
No	No	No	No	Yes	> 12 montl	> 32 hours	Use after c	25000
Yes	No	No	No	No	9-12 montl	> 32 hours	Single use	30000
Yes	No	No	No	No	9-12 montl	> 32 hours	Single use	20000
Yes	No	No	No	No	9-12 montl	> 32 hours	Use after c	34000
Yes	No	No	No	No	> 12 montl	> 32 hours	Single use	26000
Yes	No	No	No	No	> 12 montl	< 8 hours	Use after c	20000
No	Yes	No	No	No	> 12 montl	8-32 hours	Use after c	25000
Yes	No	No	No	No	> 12 montl	> 32 hours	Use after c	50000
Yes	No	No	No	No	9-12 montl	< 8 hours	Single use	25000
Yes	No	No	No	Yes	> 12 montl	8-32 hours	Use after c	30000
Yes	No	No	No	Yes	> 12 montl	8-32 hours	Use after c	18000
Yes	No	No	No	Yes	> 12 montl	8-32 hours	Single use	39000
Yes	No	No	No	No	> 12 montl	> 32 hours	Use after c	30000
Yes	No	No	No	No	> 12 montl	8-32 hours	Use after c	50000
Yes	No	No	No	Yes	> 12 montl	< 8 hours	Use after c	150000
Yes	No	No	No	No	> 12 montl	< 8 hours	Single use	50000
Yes	No	No	No	No	> 12 montl	> 32 hours	Single use	40000
Yes	No	No	No	No	> 12 montl	8-32 hours	Single use	50000
Yes	No	No	No	No	> 12 montl	8-32 hours	Single use	50000
Yes	No	No	No	No	> 12 montl	8-32 hours	Use after c	150000
Yes	No	No	No	No	> 12 montl	8-32 hours	Single use	50000
No	Yes	No	No	No	> 12 montl	8-32 hours	Use after c	50000
Yes	No	No	No	No	> 12 montl	8-32 hours	Single use	50000
Yes	No	No	No	Yes	> 12 montl	< 8 hours	Use after c	500000
Yes	No	No	No	Yes	> 12 montl	8-32 hours	Use after c	50000
Yes	No	No	No	No	> 12 montl	8-32 hours	Single use	50000
Yes	No	No	No	No	> 12 montl	8-32 hours	Single use	50000
Yes	No	No	No	Yes	> 12 montl	8-32 hours	Use after c	50000
Yes	No	No	No	No	> 12 montl	8-32 hours	Single use	50000
Yes	No	No	No	Yes	> 12 montl	8-32 hours	Use after c	50000
Yes	No	No	No	No	> 12 montl	> 32 hours	Use after c	50000
Yes	No	No	No	No	> 12 montl	8-32 hours	Single use	50000
Yes	No	No	No	No	> 12 montl	8-32 hours	Single use	50000
Yes	No	No	No	Yes	> 12 montl	8-32 hours	Use after c	50000
Yes	No	No	No	No	> 12 montl	8-32 hours	Single use	50000
Yes	No	No	No	Yes	9-12 montl	8-32 hours	Use after c	50000
Yes	No	No	No	Yes	9-12 montl	8-32 hours	Use after c	50000
Yes	No	No	No	No	> 12 montl	< 8 hours	Single use	100000
Yes	No	No	No	No	> 12 montl	8-32 hours	Single use	60000
Yes	No	No	No	No	> 12 montl	8-32 hours	Use withou	15000
Yes	No	No	No	Yes	> 12 montl	> 32 hours	Use after c	100000
Yes	No	No	No	No	> 12 montl	8-32 hours	Single use	30000
Yes	No	No	No	No	> 12 montl	8-32 hours	Single use	100000
No	Yes	No	No	No	> 12 montl	< 8 hours	Use after c	40000

No	No	No	No	Yes	> 12 montl	< 8 hours	Use after c	20000
Yes	No	No	No	No	> 12 montl	> 32 hours	Use withou	20000
Yes	Yes	No	No	No	> 12 montl	> 32 hours	Single use	45000
Yes	Yes	No	No	Yes	> 12 montl	< 8 hours	Use after c	60000
Yes	No	No	No	Yes	> 12 montl	< 8 hours	Single use	40000
Yes	No	No	No	No	> 12 montl	8-32 hours	Use after c	40000
Yes	No	No	No	Yes	> 12 montl	8-32 hours	Use after c	100000
Yes	No	No	No	No	9-12 montl	< 8 hours	Single use	50000
Yes	No	No	No	Yes	> 12 montl	8-32 hours	Use after c	50000
Yes	No	No	No	No	> 12 montl	< 8 hours	Use after c	100000
Yes	Yes	No	No	No	> 12 montl	< 8 hours	Single use	60000
Yes	Yes	Yes	No	Yes	> 12 montl	< 8 hours	Single use	60000
Yes	No	No	No	No	> 12 montl	> 32 hours	Single use	50000
Yes	No	No	No	No	> 12 montl	< 8 hours	Single use	40000
Yes	No	No	No	No	> 12 montl	> 32 hours	Single use	20000
Yes	No	No	No	No	> 12 montl	8-32 hours	Single use	30000
Yes	No	No	No	No	> 12 montl	< 8 hours	Single use	85000
Yes	No	No	No	Yes	> 12 montl	8-32 hours	Single use	50000
Yes	No	No	No	No	> 12 montl	> 32 hours	Single use	50000
No	No	No	No	Yes	> 12 montl	8-32 hours	Use after c	50000
Yes	No	No	No	Yes	> 12 montl	8-32 hours	Single use	30000
Yes	No	No	No	No	> 12 montl	< 8 hours	Single use	15000
Yes	No	No	No	Yes	> 12 montl	8-32 hours	Single use	50000
No	No	No	No	Yes	> 12 montl	8-32 hours	Use after c	50000
No	No	No	Yes	No	> 12 montl	> 32 hours	Use after c	30000
Yes	No	No	No	No	> 12 montl	8-32 hours	Use after c	20000
Yes	No	No	No	Yes	> 12 montl	< 8 hours	Single use	200000
Yes	No	No	No	No	9-12 montl	< 8 hours	Use after c	2000000
Yes	No	No	No	No	> 12 montl	8-32 hours	Single use	60000
No	No	No	Yes	No	> 12 montl	8-32 hours	Use after c	150000
Yes	No	No	Yes	Yes	> 12 montl	< 8 hours	Single use	10000
Yes	No	Yes	No	Yes	> 12 montl	8-32 hours	Single use	200000
Yes	Yes	Yes	No	No	> 12 montl	8-32 hours	Single use	200000
Yes	Yes	No	Yes	No	> 12 montl	> 32 hours	Use withou	100000
Yes	Yes	No	No	No	9-12 montl	< 8 hours	Single use	100000
Yes	No	No	No	Yes	> 12 montl	< 8 hours	Use after c	30000
Yes	No	No	No	No	> 12 montl	< 8 hours	Use after c	30000
Yes	No	No	No	No	> 12 montl	> 32 hours	Single use	25000
Yes	No	No	No	Yes	> 12 montl	> 32 hours	Use after c	65000
Yes	No	No	No	No	> 12 montl	< 8 hours	Use after c	30000
No	No	No	Yes	No	> 12 montl	8-32 hours	Use after c	30000
Yes	No	No	No	Yes	> 12 montl	8-32 hours	Single use	150000
Yes	No	No	No	Yes	> 12 montl	> 32 hours	Single use	95000
Yes	No	No	No	No	> 12 montl	8-32 hours	Single use	100000
Yes	No	No	No	No	> 12 montl	< 8 hours	Single use	150000
Yes	Yes	No	No	No	> 12 montl	8-32 hours	Single use	50000
Yes	No	No	No	No	9-12 montl	< 8 hours	Single use	40000
Yes	No	No	No	No	> 12 montl	> 32 hours	Single use	100000
Yes	No	No	No	No	> 12 montl	< 8 hours	Single use	30000
Yes	No	No	No	No	> 12 montl	> 32 hours	Single use	30000
Yes	No	No	No	No	> 12 montl	< 8 hours	Use after c	20000
No	No	No	No	Yes	> 12 montl	> 32 hours	Use after c	100000

Yes	No	No	No	Yes	> 12 montl	8-32 hours	Use after c	100000
Yes	No	No	No	No	> 12 montl	> 32 hours	Single use	200000
Yes	No	No	No	Yes	> 12 montl	< 8 hours	Use after c	85000
Yes	No	No	No	No	> 12 montl	< 8 hours	Single use	60000
Yes	No	No	No	No	> 12 montl	< 8 hours	Use after c	50000
Yes	No	No	No	No	> 12 montl	< 8 hours	Single use	40000
Yes	No	No	No	No	> 12 montl	8-32 hours	Single use	100000
Yes	Yes	No	No	No	> 12 montl	8-32 hours	Single use	47000
Yes	No	No	No	No	9-12 montl	8-32 hours	Single use	47000
Yes	No	No	No	No	> 12 montl	< 8 hours	Single use	50000
Yes	No	No	No	No	> 12 montl	8-32 hours	Use after c	30000
No	No	No	No	Yes	9-12 montl	8-32 hours	Use after c	50000
Yes	No	No	No	Yes	> 12 montl	8-32 hours	Use after c	10000
Yes	No	No	No	Yes	> 12 montl	8-32 hours	Use after c	10000
Yes	No	No	No	Yes	> 12 montl	8-32 hours	Use after c	20000
Yes	No	No	No	No	> 12 montl	8-32 hours	Single use	30000
Yes	No	No	No	No	> 12 montl	< 8 hours	Single use	50000
Yes	No	No	No	No	> 12 montl	8-32 hours	Single use	20000
Yes	No	No	No	No	> 12 montl	8-32 hours	Single use	50000
Yes	No	No	No	Yes	> 12 montl	8-32 hours	Use after c	25000
Yes	Yes	No	No	Yes	> 12 montl	8-32 hours	Use after c	120000
Yes	No	No	No	No	9-12 montl	8-32 hours	Single use	100000
Yes	No	No	No	No	> 12 montl	< 8 hours	Single use	100000
Yes	No	No	No	No	> 12 montl	> 32 hours	Single use	100000
Yes	No	No	No	No	> 12 montl	< 8 hours	Single use	100000
Yes	No	No	No	No	> 12 montl	< 8 hours	Single use	100000
Yes	No	No	No	No	> 12 montl	> 32 hours	Single use	100000
Yes	No	No	No	No	> 12 montl	< 8 hours	Use after c	30000
No	No	No	No	Yes	9-12 montl	8-32 hours	Use after c	10000
Yes	Yes	No	No	No	> 12 montl	< 8 hours	Single use	100000
Yes	No	No	No	No	> 12 montl	> 32 hours	Single use	100000
Yes	No	No	No	No	> 12 montl	< 8 hours	Single use	100000
Yes	No	No	No	No	> 12 montl	> 32 hours	Single use	100000
Yes	No	No	No	No	> 12 montl	< 8 hours	Single use	100000
Yes	No	No	No	No	> 12 montl	8-32 hours	Single use	100000
Yes	No	No	No	No	> 12 montl	< 8 hours	Single use	100000
Yes	No	No	No	No	> 12 montl	< 8 hours	Single use	100000
Yes	No	No	No	No	> 12 montl	< 8 hours	Single use	100000
Yes	No	No	No	No	> 12 montl	> 32 hours	Single use	50000
No	No	No	No	Yes	> 12 montl	< 8 hours	Use after c	100000
Yes	No	No	No	No	> 12 montl	< 8 hours	Single use	60000
Yes	No	No	No	No	> 12 montl	8-32 hours	Single use	10000
Yes	No	No	No	No	9-12 montl	< 8 hours	Use after c	15000
Yes	No	No	No	Yes	9-12 montl	8-32 hours	Use withou	12000
Yes	No	No	No	Yes	9-12 montl	8-32 hours	Use withou	12000
Yes	No	No	No	No	> 12 montl	> 32 hours	Single use	12000
Yes	No	No	No	No	> 12 montl	< 8 hours	Single use	12000
No	No	No	No	Yes	9-12 montl	8-32 hours	Use after c	10000
No	No	No	No	Yes	> 12 montl	> 32 hours	Use after c	15000
Yes	No	No	No	No	9-12 montl	> 32 hours	Use after c	20000
Yes	Yes	No	No	Yes	> 12 montl	< 8 hours	Single use	200000
Yes	No	No	No	No	> 12 montl	> 32 hours	Single use	45000

No	No	No	No	Yes	> 12 month	> 32 hours	Use after c	25000
Yes	No	No	No	Yes	> 12 month	> 32 hours	Use after c	22000
Yes	No	No	No	Yes	> 12 month	> 32 hours	Use after c	20000
Yes	No	No	No	Yes	9-12 month	> 32 hours	Use after c	24000
Yes	No	No	No	No	> 12 month	> 32 hours	Use after c	25000
Yes	No	No	No	Yes	> 12 month	> 32 hours	Use after c	26000
Yes	No	No	No	No	> 12 month	> 32 hours	Use after c	20000
Yes	No	No	No	No	9-12 month	> 32 hours	Use after c	22000
Yes	No	No	No	Yes	> 12 month	> 32 hours	Single use	28000
Yes	No	No	No	No	9-12 month	> 32 hours	Use after c	20000
Yes	No	No	No	No	9-12 month	> 32 hours	Single use	26000
Yes	No	No	No	Yes	9-12 month	> 32 hours	Single use	25000
Yes	No	No	No	No	9-12 month	> 32 hours	Use after c	22000
Yes	No	No	No	No	> 12 month	> 32 hours	Single use	30000
Yes	No	No	No	Yes	> 12 month	> 32 hours	Single use	25000
Yes	No	No	No	Yes	9-12 month	> 32 hours	Use after c	15000
Yes	No	No	No	Yes	> 12 month	> 32 hours	Single use	28000
Yes	No	No	No	Yes	> 12 month	> 32 hours	Use after c	28000
Yes	No	No	No	Yes	> 12 month	> 32 hours	Use after c	30000
Yes	No	No	No	Yes	9-12 month	> 32 hours	Use after c	30000
No	No	No	No	Yes	> 12 month	8-32 hours	Use after c	30000
Yes	No	No	No	Yes	> 12 month	> 32 hours	Use after c	20000
Yes	No	No	No	Yes	> 12 month	> 32 hours	Use after c	36000
No	No	No	Yes	No	9-12 month	> 32 hours	Use withou	45000

education	marital	religion	ac	covid_inf	covid_vacc	multiple_m	dry_skin	red_skin
Graduation	Unmarried	Hinduism	Yes	No	Both doses	No	Yes	No
Post-gradu	Married	Islam	No	No	Both doses	Yes	No	Yes
Graduation	Unmarried	Islam	No	No	Both doses	Yes	No	No
Graduation	Unmarried	Hinduism	Yes	No	Both doses	Yes	No	No
Graduation	Unmarried	Hinduism	Yes	No	Both doses	Yes	No	No
Graduation	Unmarried	Hinduism	No	No	Not started	Yes	No	No
Graduation	Unmarried	Hinduism	No	No	Both doses	No	No	No
Post-gradu	Married	Hinduism	No	No	Both doses	No	No	No
Graduation	Unmarried	Islam	No	Yes	Both doses	No	No	No
Post-gradu	Married	Islam	Yes	No	Both doses	Yes	No	No
Higher sec	Unmarried	Islam	No	No	Not started	No	No	No
Higher sec	Unmarried	Islam	No	Yes	Both doses	No	Yes	No
Post-gradu	Married	Hinduism	Yes	No	Both doses	Yes	Yes	Yes
Higher sec	Unmarried	Islam	No	No	Both doses	No	No	No
Graduation	Unmarried	Hinduism	Yes	No	Both doses	Yes	No	No
Higher sec	Married	Islam	No	No	Both doses	No	Yes	No
Higher sec	Married	Hinduism	No	No	Both doses	No	No	No
Graduation	Unmarried	Hinduism	No	No	Both doses	Yes	No	No
Post-gradu	Married	Hinduism	Yes	No	Both doses	No	No	No
Post-gradu	Married	Hinduism	No	No	Both doses	No	Yes	No
Higher sec	Married	Islam	No	No	Both doses	No	No	No
Higher sec	Unmarried	Hinduism	No	No	Not started	Yes	No	No
Post-gradu	Married	Hinduism	No	No	Both doses	No	No	No
Graduation	Married	Hinduism	No	No	Both doses	Yes	No	No
Graduation	Unmarried	Hinduism	No	No	Not started	No	No	No
Post-gradu	Married	Hinduism	No	No	Both doses	Yes	No	No
Graduation	Unmarried	Islam	No	Yes	Only 1st dc	Yes	No	No
Graduation	Unmarried	Islam	No	No	Both doses	Yes	Yes	Yes
Higher sec	Unmarried	Islam	No	No	Not started	No	No	No
Higher sec	Unmarried	Islam	No	No	Not started	Yes	No	No
Higher sec	Married	Islam	No	No	Not started	No	No	No
Graduation	Married	Hinduism	No	No	Both doses	Yes	No	No
Graduation	Unmarried	Islam	No	No	Not started	Yes	No	No
Graduation	Unmarried	Hinduism	No	No	Both doses	Yes	No	No
Graduation	Unmarried	Hinduism	No	No	Not started	No	No	No
Graduation	Unmarried	Hinduism	No	Yes	Not started	No	No	No
Higher sec	Unmarried	Islam	No	Yes	Both doses	Yes	Yes	Yes
Higher sec	Married	Hinduism	No	No	Only 1st dc	Yes	No	No
Higher sec	Unmarried	Islam	No	No	Not started	Yes	No	No
Higher sec	Unmarried	Hinduism	No	No	Both doses	No	No	No
Graduation	Unmarried	Islam	No	No	Both doses	Yes	No	No
Graduation	Unmarried	Islam	No	No	Both doses	No	No	No
Higher sec	Unmarried	Hinduism	No	No	Both doses	Yes	No	No
Higher sec	Unmarried	Hinduism	No	No	Not started	No	No	No
Higher sec	Unmarried	Hinduism	No	No	Not started	No	No	No
Graduation	Unmarried	Hinduism	Yes	No	Both doses	Yes	Yes	No
Graduation	Unmarried	Islam	No	No	Both doses	No	No	No
Graduation	Married	Islam	No	No	Only 1st dc	Yes	No	No
Graduation	Unmarried	Islam	No	No	Both doses	Yes	No	No
Post-gradu	Married	Islam	Yes	Yes	Not started	Yes	No	No
Higher sec	Unmarried	Hinduism	No	No	Not started	No	No	No

Graduation	Unmarried	Islam	No	No	Not started	No	No	No
Higher sec	Unmarried	Hinduism	Yes	No	Only 1st dc	No	No	No
Post-gradu	Married	Hinduism	Yes	No	Both doses	Yes	No	No
Higher sec	Unmarried	Islam	No	No	Both doses	No	Yes	No
Higher sec	Unmarried	Islam	No	No	Both doses	No	No	No
Graduation	Unmarried	Hinduism	No	No	Only 1st dc	No	No	No
Higher sec	Married	Hinduism	No	No	Only 1st dc	Yes	No	No
Higher sec	Unmarried	Hinduism	No	Yes	Not started	No	No	No
Higher sec	Unmarried	Hinduism	No	No	Not started	Yes	Yes	No
Higher sec	Unmarried	Hinduism	No	No	Not started	No	No	No
Higher sec	Unmarried	Hinduism	No	No	Not started	Yes	No	No
Graduation	Unmarried	Hinduism	No	No	Both doses	No	No	Yes
Higher sec	Unmarried	Hinduism	No	No	Both doses	Yes	No	No
Higher sec	Unmarried	Islam	No	No	Not started	No	No	No
Higher sec	Unmarried	Hinduism	No	No	Not started	Yes	No	No
Higher sec	Unmarried	Hinduism	No	No	Not started	No	No	No
Higher sec	Unmarried	Hinduism	No	No	Not started	No	Yes	No
Graduation	Unmarried	Islam	No	No	Both doses	No	No	No
Graduation	Unmarried	Islam	No	Yes	Not started	No	No	No
Post-gradu	Unmarried	Hinduism	No	Yes	Both doses	No	No	No
Graduation	Unmarried	Hinduism	No	No	Not started	No	No	No
Post-gradu	Unmarried	Hinduism	No	No	Not started	Yes	No	No
Higher sec	Unmarried	Islam	Yes	No	Both doses	No	No	No
Post-gradu	Married	Hinduism	No	No	Both doses	No	No	No
Higher sec	Married	Hinduism	No	No	Not started	No	No	No
Graduation	Unmarried	Hinduism	Yes	Yes	Not started	Yes	No	No
Graduation	Unmarried	Hinduism	No	No	Only 1st dc	No	No	No
Post-gradu	Unmarried	Hinduism	Yes	Yes	Not started	No	No	No
Graduation	Unmarried	Buddhism	No	No	Both doses	Yes	No	No
Graduation	Unmarried	Islam	No	No	Both doses	Yes	No	Yes
Graduation	Unmarried	Hinduism	No	Yes	Both doses	No	No	No
Higher sec	Unmarried	Hinduism	No	No	Only 1st dc	No	No	No
Graduation	Unmarried	Islam	No	No	Not started	No	No	No
Graduation	Married	Islam	No	No	Both doses	Yes	No	No
Graduation	Unmarried	Islam	No	Yes	Only 1st dc	No	No	Yes
Graduation	Married	Hinduism	No	No	Both doses	Yes	No	No
Post-gradu	Married	Hinduism	Yes	No	Both doses	Yes	No	No
Higher sec	Unmarried	Islam	Yes	No	Both doses	Yes	No	No
Graduation	Unmarried	Islam	No	No	Both doses	Yes	No	No
Graduation	Unmarried	Islam	No	No	Both doses	No	No	No
Higher sec	Unmarried	Islam	No	No	Both doses	No	No	No
Higher sec	Unmarried	Islam	Yes	No	Not started	Yes	No	No
Graduation	Unmarried	Islam	No	Yes	Both doses	Yes	No	No
Graduation	Unmarried	Hinduism	No	No	Both doses	No	No	No
Higher sec	Unmarried	Hinduism	No	No	Both doses	No	No	No
Higher sec	Unmarried	Islam	No	Yes	Both doses	No	No	No
Graduation	Unmarried	Islam	No	No	Both doses	Yes	No	No
Higher sec	Unmarried	Islam	No	No	Not started	Yes	No	No
Higher sec	Unmarried	Islam	No	Yes	Not started	Yes	No	No
Higher sec	Unmarried	Islam	Yes	No	Both doses	Yes	No	No
Graduation	Unmarried	Islam	No	No	Both doses	No	No	No
Graduation	Unmarried	Islam	No	No	Both doses	No	No	No

Higher sec	Unmarried	Islam	No	No	Not started	No	No	No
Graduation	Unmarried	Islam	No	No	Not started	No	No	No
Graduation	Married	Islam	No	Yes	Only 1st dc	Yes	No	No
Higher sec	Unmarried	Islam	Yes	No	Both doses	No	No	No
Graduation	Unmarried	Hinduism	Yes	No	Only 1st dc	Yes	No	No
Graduation	Unmarried	Hinduism	Yes	No	Only 1st dc	Yes	No	No
Graduation	Unmarried	Hinduism	No	No	Both doses	Yes	No	No
Higher sec	Unmarried	Islam	Yes	No	Not started	No	Yes	No
Higher sec	Unmarried	Islam	No	No	Both doses	No	No	No
Graduation	Unmarried	Islam	No	No	Not started	No	No	No
Graduation	Unmarried	Hinduism	Yes	No	Only 1st dc	Yes	No	No
Post-gradu	Married	Hinduism	No	No	Both doses	Yes	No	No
Graduation	Unmarried	Islam	No	No	Not started	Yes	No	No
Graduation	Married	Hinduism	No	No	Both doses	No	No	No
Higher sec	Unmarried	Islam	No	No	Both doses	Yes	No	No
Graduation	Unmarried	Islam	No	No	Both doses	No	No	No
Graduation	Unmarried	Hinduism	No	No	Both doses	Yes	No	No
Higher sec	Unmarried	Islam	Yes	No	Not started	Yes	No	Yes
Graduation	Unmarried	Islam	Yes	No	Both doses	Yes	No	No
Graduation	Unmarried	Hinduism	No	No	Only 1st dc	Yes	No	No
Graduation	Unmarried	Islam	No	No	Not started	Yes	No	No
Higher sec	Unmarried	Islam	No	No	Both doses	Yes	No	No
Post-gradu	Married	Hinduism	Yes	No	Both doses	No	No	No
Higher sec	Unmarried	Hinduism	No	No	Both doses	Yes	No	No
Post-gradu	Married	Hinduism	Yes	No	Both doses	Yes	No	No
Graduation	Married	Hinduism	No	No	Both doses	Yes	No	No
Graduation	Unmarried	Islam	No	No	Both doses	Yes	No	No
Higher sec	Unmarried	Islam	No	No	Only 1st dc	No	No	No
Graduation	Unmarried	Islam	No	No	Both doses	No	No	No
Higher sec	Unmarried	Hinduism	No	No	Not started	No	No	No
Higher sec	Unmarried	Islam	Yes	No	Both doses	No	No	No
Graduation	Unmarried	Islam	No	No	Not started	No	No	No
Graduation	Unmarried	Hinduism	No	No	Not started	Yes	No	No
Higher sec	Unmarried	Hinduism	No	No	Both doses	No	No	No
Graduation	Unmarried	Hinduism	No	No	Both doses	No	No	No
Graduation	Unmarried	Islam	No	No	Both doses	Yes	No	No
Higher sec	Unmarried	Hinduism	No	No	Only 1st dc	No	No	No
Higher sec	Unmarried	Buddhism	No	No	Both doses	Yes	No	No
Graduation	Unmarried	Hinduism	No	No	Only 1st dc	No	No	No
Higher sec	Unmarried	Islam	No	No	Both doses	No	No	No
Graduation	Unmarried	Hinduism	No	No	Both doses	No	No	No
Higher sec	Unmarried	Islam	No	No	Not started	Yes	No	No
Graduation	Unmarried	Islam	No	No	Both doses	No	No	No
Graduation	Unmarried	Hinduism	No	No	Both doses	Yes	No	No
Post-gradu	Married	Islam	No	No	Both doses	Yes	No	No
Graduation	Married	Islam	No	No	Both doses	No	No	No
Graduation	Unmarried	Islam	No	No	Not started	No	No	No
Higher sec	Unmarried	Hinduism	No	No	Both doses	Yes	No	No
Higher sec	Married	Islam	No	No	Both doses	No	No	No
Higher sec	Unmarried	Hinduism	No	No	Both doses	No	No	No
Graduation	Unmarried	Hinduism	No	No	Not started	No	No	No
Graduation	Unmarried	Islam	No	No	Both doses	No	No	No

Post-gradu	Unmarried	Hinduism	No	No	Not started	No	No	No
Higher sec	Unmarried	Hinduism	No	No	Both doses	Yes	No	No
Higher sec	Unmarried	Islam	No	No	Both doses	Yes	No	No
Graduation	Unmarried	Hinduism	No	No	Both doses	Yes	No	No
Graduation	Unmarried	Islam	No	No	Both doses	Yes	No	No
Graduation	Unmarried	Islam	No	No	Not started	Yes	No	No
Graduation	Unmarried	Islam	No	No	Both doses	No	Yes	No
Higher sec	Unmarried	Islam	No	No	Both doses	Yes	No	No
Graduation	Unmarried	Hinduism	Yes	No	Both doses	No	No	No
Higher sec	Unmarried	Islam	No	No	Not started	Yes	No	No
Higher sec	Unmarried	Islam	No	Yes	Not started	Yes	Yes	No
Graduation	Unmarried	Islam	No	No	Both doses	Yes	No	No
Higher sec	Unmarried	Islam	No	No	Both doses	Yes	No	No
Higher sec	Unmarried	Islam	No	No	Both doses	No	No	No
Graduation	Unmarried	Hinduism	No	No	Not started	No	Yes	Yes
Graduation	Unmarried	Islam	No	No	Both doses	Yes	No	No
Graduation	Unmarried	Islam	No	No	Both doses	Yes	No	No
Graduation	Unmarried	Islam	No	No	Both doses	Yes	No	No
Graduation	Unmarried	Islam	No	No	Both doses	Yes	No	No
Graduation	Unmarried	Hinduism	No	No	Not started	Yes	No	No
Graduation	Unmarried	Islam	No	No	Not started	No	No	No
Graduation	Unmarried	Islam	No	No	Not started	No	No	No
Graduation	Unmarried	Hinduism	No	No	Only 1st do	No	No	No
Graduation	Unmarried	Islam	Yes	No	Both doses	No	No	No
Higher sec	Unmarried	Islam	No	No	Not started	Yes	No	No
Graduation	Unmarried	Hinduism	No	No	Both doses	No	No	No
Graduation	Unmarried	Islam	No	No	Not started	Yes	Yes	No
Graduation	Unmarried	Islam	Yes	Yes	Only 1st do	No	No	No
Graduation	Unmarried	Hinduism	No	No	Both doses	No	No	No
Graduation	Unmarried	Islam	No	No	Both doses	Yes	No	No
Higher sec	Unmarried	Hinduism	No	No	Not started	Yes	No	No
Graduation	Unmarried	Islam	No	No	Both doses	Yes	No	Yes
Graduation	Unmarried	Buddhism	No	No	Both doses	No	No	No
Higher sec	Unmarried	Islam	No	No	Both doses	No	No	No
Graduation	Unmarried	Islam	No	No	Only 1st do	No	No	Yes
Graduation	Unmarried	Islam	No	No	Both doses	No	No	No
Graduation	Unmarried	Hinduism	No	No	Not started	No	No	No
Graduation	Unmarried	Islam	No	No	Only 1st do	Yes	No	No
Graduation	Unmarried	Islam	Yes	No	Both doses	Yes	No	No
Graduation	Unmarried	Islam	No	No	Both doses	No	No	No
Graduation	Unmarried	Hinduism	No	No	Both doses	No	No	No
Graduation	Unmarried	Islam	No	Yes	Not started	No	No	No
Graduation	Unmarried	Islam	No	No	Both doses	No	No	No
Graduation	Unmarried	Islam	No	No	Not started	No	No	No
Post-gradu	Unmarried	Hinduism	Yes	No	Not started	Yes	No	No
Graduation	Unmarried	Islam	No	No	Both doses	No	No	No
Graduation	Married	Hinduism	No	No	Both doses	No	No	No
Higher sec	Unmarried	Hinduism	No	No	Only 1st do	No	No	No
Graduation	Unmarried	Hinduism	No	No	Only 1st do	Yes	No	No
Higher sec	Unmarried	Hinduism	No	No	Only 1st do	No	No	No
Graduation	Unmarried	Islam	No	No	Not started	Yes	No	No
Higher sec	Unmarried	Islam	Yes	No	Both doses	Yes	No	No

Graduation	Unmarried	Islam	No	No	Both doses	Yes	No	Yes
Higher sec	Unmarried	Islam	No	Yes	Only 1st dc	Yes	No	No
Higher sec	Married	Islam	No	No	Only 1st dc	No	Yes	Yes
Higher sec	Married	Islam	No	Yes	Not started	No	No	No
Graduation	Married	Islam	Yes	No	Both doses	Yes	Yes	No
Post-gradu	Married	Hinduism	Yes	Yes	Both doses	No	No	No
Higher sec	Unmarried	Hinduism	Yes	Yes	Both doses	Yes	No	No
Graduation	Unmarried	Islam	No	No	Both doses	Yes	No	No
Higher sec	Unmarried	Islam	No	No	Both doses	Yes	No	No
Post-gradu	Married	Hinduism	Yes	No	Both doses	No	No	No
Graduation	Unmarried	Islam	No	No	Both doses	No	No	No
Higher sec	Married	Hinduism	No	No	Both doses	Yes	No	Yes
Graduation	Unmarried	Islam	No	No	Both doses	Yes	No	No
Graduation	Unmarried	Islam	No	No	Both doses	No	No	No
Graduation	Unmarried	Islam	No	No	Both doses	Yes	No	No
Graduation	Unmarried	Islam	No	No	Both doses	No	No	No
Graduation	Married	Hinduism	No	No	Only 1st dc	Yes	No	No
Graduation	Unmarried	Islam	No	No	Both doses	Yes	No	No
Graduation	Unmarried	Islam	Yes	Yes	Both doses	No	No	No
Graduation	Unmarried	Hinduism	No	No	Both doses	No	No	Yes
Graduation	Unmarried	Islam	No	No	Both doses	No	No	No
Higher sec	Unmarried	Islam	No	No	Not started	No	No	No
Graduation	Unmarried	Islam	No	No	Both doses	No	No	No
Graduation	Unmarried	Hinduism	No	No	Both doses	No	No	No
Post-gradu	Married	Hinduism	No	No	Both doses	No	No	Yes
Graduation	Married	Hinduism	No	No	Both doses	Yes	No	No
Higher sec	Married	Islam	No	Yes	Both doses	Yes	Yes	No
Post-gradu	Married	Islam	Yes	No	Both doses	Yes	No	No
Post-gradu	Married	Hinduism	Yes	No	Not started	Yes	No	Yes
Graduation	Unmarried	Islam	No	No	Both doses	Yes	Yes	No
Higher sec	Unmarried	Islam	No	No	Both doses	No	No	No
Graduation	Unmarried	Islam	No	Yes	Both doses	Yes	No	No
Graduation	Unmarried	Hinduism	No	No	Both doses	Yes	No	No
Graduation	Unmarried	Islam	Yes	No	Both doses	Yes	No	No
Higher sec	Unmarried	Hinduism	Yes	No	Not started	No	No	No
Higher sec	Unmarried	Islam	No	Yes	Both doses	No	No	No
Graduation	Unmarried	Islam	No	No	Both doses	Yes	No	No
Graduation	Unmarried	Hinduism	No	No	Not started	Yes	No	No
Post-gradu	Unmarried	Hinduism	No	No	Both doses	No	No	Yes
Graduation	Unmarried	Islam	Yes	No	Only 1st dc	Yes	No	No
Higher sec	Unmarried	Islam	No	No	Both doses	Yes	No	No
Graduation	Unmarried	Hinduism	No	No	Only 1st dc	No	No	No
Graduation	Unmarried	Hinduism	No	No	Both doses	No	No	No
Higher sec	Unmarried	Hinduism	No	No	Only 1st dc	No	No	No
Graduation	Unmarried	Islam	No	No	Both doses	Yes	Yes	No
Graduation	Unmarried	Islam	No	No	Both doses	No	No	No
Graduation	Unmarried	Islam	Yes	Yes	Both doses	No	No	No
Post-gradu	Married	Islam	No	No	Both doses	Yes	No	Yes
Higher sec	Unmarried	Islam	No	No	Both doses	No	No	No
Graduation	Unmarried	Hinduism	No	No	Both doses	Yes	No	No
Higher sec	Unmarried	Islam	No	No	Both doses	Yes	Yes	No
Graduation	Unmarried	Islam	No	No	Both doses	No	No	No

Graduation	Unmarried	Hinduism	No	Yes	Both doses	No	No	No
Graduation	Unmarried	Islam	Yes	No	Both doses	Yes	No	No
Higher sec	Unmarried	Islam	No	No	Both doses	Yes	No	Yes
Graduation	Unmarried	Islam	No	Yes	Only 1st dc	No	No	No
Graduation	Unmarried	Hinduism	No	No	Both doses	No	No	No
Graduation	Unmarried	Islam	No	No	Both doses	No	No	No
Graduation	Married	Islam	No	No	Both doses	No	No	No
Post-gradu	Married	Islam	Yes	No	Not started	No	No	No
Post-gradu	Married	Islam	Yes	Yes	Both doses	No	No	No
Graduation	Unmarried	Islam	Yes	No	Both doses	Yes	No	No
Graduation	Unmarried	Islam	No	No	Both doses	No	No	No
Graduation	Unmarried	Hinduism	No	No	Both doses	No	No	No
Post-gradu	Married	Hinduism	Yes	Yes	Both doses	No	No	No
Post-gradu	Married	Hinduism	No	No	Both doses	Yes	Yes	No
Graduation	Married	Hinduism	No	Yes	Both doses	No	Yes	Yes
Post-gradu	Married	Hinduism	No	No	Both doses	No	No	No
Higher sec	Married	Hinduism	No	No	Both doses	No	No	No
Higher sec	Married	Islam	No	No	Both doses	Yes	Yes	No
Graduation	Married	Islam	No	No	Both doses	Yes	Yes	No
Graduation	Unmarried	Hinduism	No	No	Both doses	Yes	Yes	No
Post-gradu	Unmarried	Islam	No	No	Not started	No	No	No
Graduation	Unmarried	Islam	No	No	Both doses	Yes	No	No
Graduation	Unmarried	Islam	No	Yes	Not started	Yes	No	No
Graduation	Unmarried	Islam	No	No	Not started	No	No	No
Graduation	Unmarried	Islam	No	No	Not started	Yes	No	No
Higher sec	Unmarried	Islam	No	No	Not started	No	No	Yes
Higher sec	Unmarried	Islam	No	No	Not started	No	No	No
Higher sec	Unmarried	Islam	No	No	Not started	No	No	No
Graduation	Unmarried	Islam	No	No	Not started	No	No	No
Higher sec	Unmarried	Islam	No	No	Not started	No	No	No
Graduation	Unmarried	Islam	No	No	Both doses	Yes	No	No
Higher sec	Unmarried	Islam	No	No	Not started	No	No	No
Higher sec	Unmarried	Islam	No	No	Not started	No	No	No
Higher sec	Unmarried	Islam	No	No	Not started	Yes	No	No
Higher sec	Unmarried	Islam	No	No	Not started	No	No	No
Graduation	Unmarried	Islam	No	No	Not started	Yes	No	No
Graduation	Married	Islam	No	No	Not started	Yes	No	No
Graduation	Unmarried	Islam	Yes	No	Not started	Yes	No	No
Graduation	Unmarried	Hinduism	No	No	Not started	Yes	No	No
Higher sec	Unmarried	Islam	No	No	Not started	No	No	No
Graduation	Unmarried	Islam	No	No	Both doses	No	No	No
Graduation	Married	Islam	Yes	No	Not started	Yes	No	No
Post-gradu	Married	Islam	No	Yes	Only 1st dc	Yes	No	No
Post-gradu	Married	Hinduism	No	No	Both doses	No	No	No
Post-gradu	Married	Hinduism	No	No	Both doses	No	No	No
Higher sec	Unmarried	Hinduism	No	No	Both doses	No	No	No
Post-gradu	Married	Hinduism	No	No	Both doses	Yes	No	No
Higher sec	Unmarried	Hinduism	No	No	Only 1st dc	Yes	No	Yes
Higher sec	Unmarried	Hinduism	No	No	Not started	Yes	No	No
Higher sec	Unmarried	Islam	No	No	Not started	No	No	No
Graduation	Unmarried	Islam	No	Yes	Both doses	No	Yes	No
Post-gradu	Married	Hinduism	Yes	No	Both doses	Yes	No	No

Post-gradu	Married	Hinduism	No	No	Both doses	Yes	No	No
Graduation	Married	Hinduism	No	No	Both doses	Yes	No	No
Higher sec	Married	Hinduism	No	No	Both doses	Yes	No	No
Post-gradu	Married	Hinduism	No	No	Both doses	Yes	No	No
Graduation	Unmarried	Buddhism	No	No	Only 1st dc	Yes	No	No
Higher sec	Unmarried	Hinduism	No	No	Not started	No	No	No
Graduation	Unmarried	Islam	No	No	Both doses	Yes	No	No
Higher sec	Unmarried	Hinduism	No	No	Not started	Yes	No	No
Graduation	Unmarried	Islam	No	No	Not started	No	No	No
Higher sec	Unmarried	Islam	No	No	Not started	No	No	No
Graduation	Married	Islam	Yes	No	Both doses	Yes	No	No
Graduation	Married	Islam	Yes	No	Both doses	No	No	No
Higher sec	Unmarried	Islam	Yes	No	Not started	No	No	No
Graduation	Unmarried	Islam	No	No	Not started	No	No	No
Graduation	Unmarried	Hinduism	No	No	Not started	No	Yes	No
Graduation	Married	Islam	No	No	Both doses	No	No	No
Graduation	Unmarried	Islam	Yes	No	Only 1st dc	Yes	No	No
Graduation	Unmarried	Islam	No	No	Only 1st dc	No	No	No
Graduation	Unmarried	Islam	No	No	Not started	No	No	No
Post-gradu	Married	Islam	Yes	No	Both doses	No	No	No
Higher sec	Unmarried	Islam	No	No	Both doses	Yes	No	No
Higher sec	Unmarried	Hinduism	No	No	Not started	No	No	No
Post-gradu	Unmarried	Islam	Yes	No	Not started	No	Yes	No
Higher sec	Unmarried	Hinduism	No	No	Only 1st dc	Yes	Yes	No
Higher sec	Unmarried	Hinduism	No	No	Not started	Yes	No	No
Higher sec	Unmarried	Hinduism	No	No	Not started	Yes	No	No
Higher sec	Unmarried	Islam	No	No	Both doses	No	No	No
Higher sec	Unmarried	Hinduism	No	No	Not started	No	No	No
Higher sec	Unmarried	Hinduism	No	No	Not started	No	No	No
Uneducate	Married	Islam	No	No	Not started	No	No	No
Post-gradu	Unmarried	Islam	No	No	Not started	No	No	No
Higher sec	Married	Islam	No	Yes	Both doses	Yes	No	No
Higher sec	Unmarried	Islam	No	No	Not started	Yes	No	No
Post-gradu	Unmarried	Islam	Yes	Yes	Both doses	No	No	No
Graduation	Unmarried	Hinduism	No	No	Not started	Yes	No	No
Graduation	Unmarried	Islam	Yes	No	Both doses	No	No	No
Graduation	Unmarried	Hinduism	No	No	Only 1st dc	No	No	No
Graduation	Unmarried	Islam	No	Yes	Not started	Yes	No	No
Graduation	Unmarried	Islam	No	No	Both doses	No	No	No
Higher sec	Unmarried	Islam	No	Yes	Both doses	Yes	No	No
Graduation	Unmarried	Hinduism	No	No	Both doses	Yes	No	No
Higher sec	Unmarried	Islam	No	Yes	Not started	Yes	No	No
Graduation	Unmarried	Islam	No	Yes	Not started	Yes	No	No
Graduation	Unmarried	Islam	No	Yes	Both doses	Yes	Yes	No
Graduation	Unmarried	Islam	No	Yes	Not started	Yes	No	No
Graduation	Unmarried	Islam	No	Yes	Both doses	Yes	Yes	No
Graduation	Unmarried	Hinduism	Yes	No	Both doses	Yes	No	Yes
Higher sec	Unmarried	Islam	Yes	No	Both doses	No	No	No
Higher sec	Unmarried	Hinduism	No	No	Only 1st dc	Yes	No	No
Graduation	Unmarried	Islam	No	No	Both doses	Yes	No	No
Graduation	Unmarried	Islam	No	No	Both doses	No	No	No
Graduation	Unmarried	Islam	No	Yes	Not started	No	No	No

Higher sec	Unmarried	Islam	No	No	Not started	No	No	No
Higher sec	Unmarried	Hinduism	No	No	Both doses	Yes	No	No
Graduation	Unmarried	Islam	No	No	Not started	No	No	No
Graduation	Unmarried	Islam	No	No	Both doses	No	Yes	Yes
Higher sec	Unmarried	Buddhism	No	Yes	Both doses	No	No	No
Graduation	Unmarried	Hinduism	Yes	No	Not started	Yes	Yes	Yes
Higher sec	Unmarried	Islam	No	Yes	Both doses	No	No	No
Graduation	Unmarried	Hinduism	No	No	Not started	Yes	No	No
Post-gradu	Unmarried	Islam	No	Yes	Not started	No	No	No
Higher sec	Unmarried	Hinduism	No	Yes	Not started	Yes	No	No
Graduation	Married	Hinduism	No	No	Both doses	Yes	No	No
Graduation	Married	Hinduism	No	No	Both doses	Yes	No	No
Higher sec	Unmarried	Hinduism	No	No	Not started	Yes	No	No
Higher sec	Unmarried	Hinduism	No	No	Not started	Yes	No	No
Graduation	Unmarried	Islam	Yes	No	Both doses	Yes	No	No
Graduation	Unmarried	Islam	Yes	No	Both doses	Yes	No	No
Higher sec	Unmarried	Hinduism	No	No	Not started	No	No	No
Graduation	Unmarried	Hinduism	No	No	Both doses	Yes	No	No
Post-gradu	Married	Hinduism	No	No	Both doses	Yes	No	No
Post-gradu	Married	Buddhism	No	No	Both doses	Yes	No	No
Post-gradu	Married	Buddhism	No	No	Both doses	Yes	No	No
Post-gradu	Married	Buddhism	Yes	Yes	Both doses	Yes	No	No
Higher sec	Married	Islam	No	Yes	Not started	No	Yes	No
Graduation	Unmarried	Hinduism	Yes	No	Both doses	Yes	No	No
Higher sec	Married	Islam	No	No	Only 1st dc	Yes	No	No
Post-gradu	Married	Hinduism	Yes	Yes	Both doses	Yes	No	No
Higher sec	Unmarried	Hinduism	Yes	No	Not started	No	No	Yes
Post-gradu	Unmarried	Islam	No	No	Only 1st dc	No	No	No
Higher sec	Unmarried	Hinduism	No	No	Only 1st dc	No	No	No
Graduation	Married	Islam	No	No	Not started	No	No	No
Higher sec	Unmarried	Islam	No	Yes	Not started	Yes	No	No
Higher sec	Unmarried	Islam	No	Yes	Not started	No	No	No
Graduation	Unmarried	Hinduism	No	Yes	Not started	No	No	Yes
Higher sec	Unmarried	Islam	No	No	Not started	Yes	No	No
Higher sec	Unmarried	Islam	No	No	Not started	Yes	No	No
Graduation	Unmarried	Hinduism	No	No	Only 1st dc	No	No	No
Post-gradu	Married	Hinduism	Yes	No	Both doses	No	No	No
Post-gradu	Married	Hinduism	No	No	Both doses	No	No	No
Post-gradu	Married	Hinduism	No	No	Both doses	Yes	No	No
Graduation	Unmarried	Hinduism	No	No	Both doses	Yes	No	No
Higher sec	Unmarried	Hinduism	No	No	Not started	No	No	Yes
Graduation	Unmarried	Islam	No	No	Both doses	No	No	No
Post-gradu	Unmarried	Islam	No	Yes	Not started	No	No	No
Post-gradu	Unmarried	Islam	No	No	Only 1st dc	No	No	No
Graduation	Unmarried	Buddhism	No	No	Both doses	Yes	No	No
Post-gradu	Unmarried	Hinduism	Yes	No	Both doses	No	No	No
Graduation	Unmarried	Islam	No	No	Only 1st dc	No	No	Yes
Graduation	Married	Hinduism	Yes	Yes	Both doses	Yes	No	No
Post-gradu	Unmarried	Hinduism	Yes	No	Both doses	No	No	No
Graduation	Married	Hinduism	No	No	Both doses	Yes	No	No
Graduation	Married	Islam	Yes	No	Both doses	No	No	No
Post-gradu	Unmarried	Islam	No	No	Only 1st dc	No	No	No

Post-gradu	Married	Hinduism	No	No	Both doses	No	No	No
Post-gradu	Unmarried	Hinduism	No	Yes	Only 1st dc	Yes	No	No
Post-gradu	Married	Hinduism	Yes	No	Both doses	Yes	No	No
Post-gradu	Unmarried	Islam	Yes	No	Only 1st dc	No	No	Yes
Post-gradu	Married	Islam	Yes	No	Only 1st dc	No	No	No
Post-gradu	Unmarried	Islam	No	No	Both doses	Yes	No	No
Post-gradu	Married	Islam	No	Yes	Both doses	Yes	Yes	No
Graduation	Unmarried	Islam	No	No	Not started	Yes	No	Yes
Graduation	Unmarried	Islam	No	No	Not started	Yes	No	No
Graduation	Unmarried	Hinduism	No	Yes	Both doses	No	No	No
Graduation	Unmarried	Islam	No	No	Not started	No	No	No
Higher sec	Unmarried	Islam	No	No	Not started	Yes	No	No
Graduation	Unmarried	Islam	No	No	Not started	No	No	No
Post-gradu	Unmarried	Hinduism	No	Yes	Only 1st dc	Yes	No	No
Graduation	Unmarried	Hinduism	No	No	Not started	No	No	No
Higher sec	Unmarried	Islam	No	No	Only 1st dc	Yes	No	No
Higher sec	Unmarried	Hinduism	Yes	No	Only 1st dc	No	No	No
Graduation	Unmarried	Islam	No	No	Both doses	No	No	No
Graduation	Unmarried	Islam	No	No	Not started	No	No	No
Graduation	Unmarried	Hinduism	No	No	Both doses	Yes	No	No
Graduation	Unmarried	Islam	No	No	Not started	Yes	No	No
Graduation	Unmarried	Islam	No	No	Not started	No	No	No
Graduation	Unmarried	Islam	No	No	Both doses	No	No	No
Graduation	Unmarried	Hinduism	No	Yes	Both doses	Yes	No	No
Higher sec	Unmarried	Hinduism	No	Yes	Not started	Yes	No	Yes
Higher sec	Unmarried	Hinduism	Yes	No	Not started	No	No	No
Higher sec	Unmarried	Hinduism	No	No	Not started	No	No	No
Post-gradu	Unmarried	Islam	No	No	Not started	Yes	No	No
Graduation	Unmarried	Islam	No	No	Both doses	No	No	No
Higher sec	Unmarried	Hinduism	No	No	Not started	Yes	No	No
Graduation	Unmarried	Islam	No	No	Not started	No	No	No
Post-gradu	Married	Hinduism	No	No	Only 1st dc	No	No	No
Higher sec	Married	Hinduism	Yes	No	Not started	No	No	No
Graduation	Unmarried	Hinduism	No	No	Not started	Yes	No	No
Post-gradu	Unmarried	Islam	No	No	Both doses	No	No	No
Higher sec	Married	Hinduism	No	No	Not started	Yes	No	No
Post-gradu	Married	Islam	No	No	Only 1st dc	No	No	No
Higher sec	Married	Islam	No	No	Not started	No	No	No
Post-gradu	Unmarried	Islam	No	No	Both doses	No	No	No
Post-gradu	Married	Islam	No	No	Both doses	No	No	No
Post-gradu	Unmarried	Islam	No	No	Both doses	No	No	No
Graduation	Unmarried	Islam	No	No	Not started	No	No	No
Graduation	Married	Hinduism	No	No	Both doses	Yes	No	No
Graduation	Unmarried	Islam	Yes	Yes	Both doses	No	No	No
Graduation	Unmarried	Hinduism	Yes	No	Not started	No	No	No
Graduation	Unmarried	Islam	No	No	Both doses	No	No	No
Higher sec	Married	Islam	Yes	No	Only 1st dc	Yes	No	No
Post-gradu	Married	Hinduism	No	Yes	Both doses	Yes	No	No
Graduation	Unmarried	Islam	No	No	Both doses	Yes	No	No
Graduation	Unmarried	Islam	No	No	Both doses	Yes	Yes	No
Graduation	Unmarried	Hinduism	No	No	Not started	No	No	No
Graduation	Unmarried	Hinduism	Yes	No	Both doses	Yes	No	No

Post-gradu	Unmarried	Islam	No	No	Only 1st dc	No	No	No
Post-gradu	Married	Buddhism	No	No	Both doses	No	No	No
Higher sec	Married	Islam	No	No	Both doses	No	Yes	No
Higher sec	Married	Islam	No	No	Not started	No	No	No
Higher sec	Unmarried	Islam	No	No	Not started	No	No	No
Graduation	Unmarried	Islam	No	No	Both doses	No	No	No
Higher sec	Unmarried	Islam	No	Yes	Not started	Yes	No	No
Higher sec	Unmarried	Islam	No	No	Not started	Yes	No	No
Higher sec	Married	Islam	No	No	Not started	No	No	No
Higher sec	Married	Islam	No	No	Not started	No	No	No
Higher sec	Unmarried	Islam	No	No	Not started	No	No	No
Higher sec	Married	Islam	No	No	Not started	No	No	No
Higher sec	Married	Islam	No	No	Not started	No	No	No
Higher sec	Married	Islam	No	No	Not started	Yes	No	No
Higher sec	Unmarried	Islam	No	Yes	Not started	No	No	No
Higher sec	Married	Islam	Yes	No	Not started	No	No	No
Higher sec	Married	Islam	No	No	Not started	No	No	No
Higher sec	Unmarried	Islam	No	No	Not started	No	No	No
Higher sec	Married	Islam	No	No	Not started	No	No	No
Higher sec	Married	Islam	Yes	No	Not started	No	No	No
Uneducate	Married	Islam	No	No	Not started	No	No	No
Uneducate	Married	Islam	Yes	No	Not started	No	No	No
Higher sec	Unmarried	Islam	No	No	Not started	No	No	No
Graduation	Unmarried	Islam	No	No	Not started	Yes	No	No
Graduation	Unmarried	Islam	No	No	Only 1st dc	Yes	No	No
Graduation	Married	Islam	No	No	Not started	No	No	Yes
Graduation	Unmarried	Islam	No	No	Only 1st dc	Yes	No	Yes
Higher sec	Unmarried	Islam	No	No	Only 1st dc	No	No	No
Graduation	Unmarried	Islam	Yes	No	Only 1st dc	No	No	Yes
Graduation	Unmarried	Islam	No	No	Not started	No	No	No
Post-gradu	Married	Islam	No	Yes	Not started	No	No	No
Higher sec	Married	Islam	Yes	Yes	Not started	Yes	No	No
Higher sec	Unmarried	Islam	No	No	Not started	No	No	No
Higher sec	Married	Islam	No	No	Not started	No	Yes	No
Higher sec	Married	Islam	No	No	Not started	No	No	Yes
Higher sec	Married	Islam	No	No	Not started	No	No	No
Graduation	Unmarried	Islam	No	No	Only 1st dc	No	No	Yes
Higher sec	Unmarried	Islam	No	Yes	Not started	No	No	No
Higher sec	Married	Islam	No	No	Not started	No	No	No
Uneducate	Married	Islam	No	No	Not started	No	No	No
Graduation	Unmarried	Hinduism	Yes	No	Both doses	No	No	No
Post-gradu	Married	Hinduism	No	Yes	Both doses	Yes	No	No
Graduation	Married	Hinduism	No	No	Both doses	Yes	No	No
Graduation	Married	Hinduism	Yes	No	Both doses	Yes	No	No
Graduation	Married	Hinduism	No	No	Both doses	Yes	No	No
Graduation	Married	Islam	No	No	Both doses	No	No	No
Higher sec	Married	Islam	Yes	No	Not started	No	No	No
Graduation	Married	Islam	No	No	Both doses	No	No	No
Higher sec	Married	Islam	No	No	Both doses	No	No	No
Higher sec	Married	Islam	No	Yes	Both doses	No	No	No
Graduation	Unmarried	Islam	Yes	Yes	Both doses	No	No	No
Higher sec	Unmarried	Islam	Yes	No	Only 1st dc	No	No	No

Graduation	Married	Islam	No	No	Both doses	Yes	No	No
Graduation	Unmarried	Islam	No	No	Both doses	Yes	No	No
Graduation	Unmarried	Islam	No	No	Both doses	No	No	No
Post-gradu	Married	Islam	Yes	No	Both doses	No	No	No
Graduation	Married	Islam	No	No	Both doses	No	No	No
Higher sec	Married	Islam	No	No	Both doses	No	No	No
Higher sec	Unmarried	Islam	No	No	Not started	No	No	No
Graduation	Unmarried	Hinduism	Yes	No	Both doses	Yes	No	No
Graduation	Married	Islam	No	No	Both doses	Yes	No	No
Post-gradu	Married	Islam	No	Yes	Only 1st dc	No	No	No
Uneducate	Married	Islam	No	No	Not started	Yes	No	No
Higher sec	Married	Islam	No	No	Not started	No	No	No
Higher sec	Unmarried	Islam	No	No	Not started	No	No	No
Graduation	Married	Hinduism	No	No	Both doses	Yes	No	No
Graduation	Married	Hinduism	No	No	Both doses	Yes	No	No
Higher sec	Unmarried	Islam	Yes	No	Only 1st dc	Yes	No	No
Higher sec	Married	Islam	No	No	Not started	No	No	No
Higher sec	Married	Islam	No	No	Both doses	No	No	No
Higher sec	Married	Islam	No	No	Both doses	No	No	No
Post-gradu	Unmarried	Islam	Yes	No	Both doses	No	No	No
Higher sec	Married	Islam	No	No	Both doses	No	Yes	No
Graduation	Married	Islam	No	No	Both doses	No	No	No
Higher sec	Married	Islam	No	No	Both doses	No	No	No
Graduation	Married	Islam	No	No	Both doses	No	No	No
Graduation	Married	Islam	Yes	No	Both doses	No	No	No
Graduation	Unmarried	Islam	Yes	No	Not started	Yes	No	No
Graduation	Married	Islam	No	No	Both doses	No	No	No
Higher sec	Married	Islam	Yes	No	Not started	Yes	Yes	No
Higher sec	Married	Islam	No	No	Not started	Yes	No	Yes
Graduation	Married	Islam	No	Yes	Only 1st dc	No	Yes	No
Higher sec	Married	Islam	No	No	Not started	No	No	No
Graduation	Unmarried	Islam	No	No	Both doses	Yes	No	No
Post-gradu	Married	Islam	No	No	Both doses	Yes	No	No
Graduation	Married	Islam	No	No	Both doses	Yes	No	No
Higher sec	Married	Islam	No	No	Not started	No	No	No
Graduation	Unmarried	Islam	Yes	No	Only 1st dc	Yes	No	No
Uneducate	Married	Islam	No	No	Not started	No	No	Yes
Higher sec	Unmarried	Islam	No	No	Not started	Yes	No	No
Higher sec	Married	Islam	No	No	Not started	No	No	No
Higher sec	Married	Islam	No	No	Not started	No	No	Yes
Higher sec	Married	Islam	No	Yes	Only 1st dc	No	No	No
Higher sec	Married	Islam	No	Yes	Only 1st dc	No	No	Yes
Higher sec	Married	Islam	No	No	Not started	No	No	No
Higher sec	Married	Islam	No	No	Not started	No	No	No
Higher sec	Married	Islam	No	No	Not started	No	No	No
Graduation	Married	Islam	No	No	Both doses	No	No	No
Graduation	Married	Islam	No	No	Both doses	No	No	No
Higher sec	Married	Islam	Yes	No	Both doses	No	No	No
Higher sec	Married	Islam	No	No	Only 1st dc	No	No	No
Higher sec	Married	Islam	No	No	Both doses	No	No	No
Graduation	Married	Islam	No	No	Both doses	No	No	No
Graduation	Married	Islam	No	No	Both doses	No	No	No

Graduation	Unmarried	Islam	Yes	No	Both doses	No	No	No
Graduation	Unmarried	Hinduism	No	No	Not started	No	No	No
Higher sec	Married	Islam	No	No	Both doses	No	No	No
Graduation	Married	Islam	No	No	Both doses	No	No	No
Post-gradu	Married	Islam	No	No	Both doses	No	No	No
Graduation	Married	Islam	No	No	Both doses	No	No	No
Graduation	Married	Islam	No	No	Both doses	No	No	No
Graduation	Married	Islam	No	No	Both doses	No	No	No
Graduation	Unmarried	Islam	No	Yes	Not started	No	No	No
Post-gradu	Married	Hinduism	Yes	No	Both doses	Yes	Yes	No
Graduation	Married	Islam	No	No	Both doses	Yes	No	No
Graduation	Unmarried	Hinduism	No	No	Both doses	No	No	No
Graduation	Married	Islam	No	Yes	Not started	No	No	No
Graduation	Unmarried	Islam	Yes	No	Both doses	No	No	No
Higher sec	Unmarried	Islam	No	No	Not started	Yes	No	No
Graduation	Unmarried	Islam	No	No	Not started	Yes	Yes	Yes
Graduation	Unmarried	Islam	Yes	No	Only 1st dc	No	No	Yes
Higher sec	Unmarried	Islam	No	No	Not started	Yes	No	No
Post-gradu	Unmarried	Islam	No	No	Not started	No	No	No
Graduation	Married	Islam	No	No	Not started	No	No	No
Higher sec	Married	Islam	No	No	Only 1st dc	No	No	No
Higher sec	Unmarried	Hinduism	No	Yes	Not started	No	No	No
Graduation	Unmarried	Hinduism	No	No	Not started	No	No	No
Higher sec	Unmarried	Islam	No	No	Not started	No	No	No
Higher sec	Unmarried	Islam	Yes	No	Not started	No	No	No
Higher sec	Unmarried	Islam	No	No	Not started	No	No	No
Graduation	Unmarried	Islam	No	No	Not started	No	No	No
Higher sec	Unmarried	Islam	No	No	Not started	Yes	No	Yes
Higher sec	Married	Islam	No	No	Not started	Yes	No	No
Higher sec	Married	Islam	No	No	Not started	Yes	No	No
Higher sec	Unmarried	Islam	No	No	Not started	Yes	No	No
Higher sec	Unmarried	Islam	No	No	Not started	No	No	No
Higher sec	Married	Islam	No	No	Not started	No	No	No
Uneducate	Unmarried	Islam	No	No	Not started	No	No	No
Higher sec	Married	Islam	Yes	No	Both doses	No	No	No
Higher sec	Unmarried	Islam	No	No	Not started	No	No	No
Higher sec	Unmarried	Hinduism	No	No	Not started	Yes	No	No
Higher sec	Unmarried	Hinduism	No	No	Not started	Yes	No	No
Graduation	Married	Islam	Yes	No	Both doses	No	No	No
Graduation	Married	Islam	No	No	Both doses	No	No	No
Higher sec	Married	Islam	No	No	Both doses	No	No	No
Post-gradu	Married	Islam	No	No	Both doses	No	No	No
Higher sec	Married	Islam	No	No	Both doses	No	No	No
Higher sec	Married	Islam	No	No	Both doses	No	No	No
Graduation	Married	Islam	No	No	Both doses	No	No	No
Graduation	Married	Islam	No	No	Both doses	No	No	No
Higher sec	Married	Islam	Yes	No	Not started	No	Yes	No
Higher sec	Married	Islam	No	No	Not started	No	No	No
Uneducate	Married	Islam	No	No	Not started	No	No	No
Higher sec	Married	Islam	No	Yes	Only 1st dc	No	No	No
Uneducate	Married	Islam	No	No	Not started	No	Yes	No
Higher sec	Married	Islam	No	No	Not started	No	No	No

Higher sec	Married	Islam	No	No	Not started	No	No	No
Higher sec	Married	Islam	No	No	Only 1st dc	Yes	No	No
Higher sec	Unmarried	Islam	No	No	Not started	No	No	No
Higher sec	Married	Islam	No	Yes	Both doses	No	No	No
Higher sec	Married	Islam	No	No	Only 1st dc	No	Yes	No
Higher sec	Married	Islam	No	No	Not started	No	No	No
Higher sec	Married	Islam	No	No	Not started	No	Yes	No
Higher sec	Married	Islam	No	No	Not started	Yes	No	No
Higher sec	Married	Islam	No	No	Not started	Yes	No	No
Higher sec	Married	Islam	No	No	Not started	No	No	No
Higher sec	Married	Islam	No	No	Not started	No	Yes	No
Higher sec	Married	Islam	No	No	Not started	No	No	No
Higher sec	Married	Islam	No	No	Only 1st dc	No	No	No
Higher sec	Married	Islam	No	No	Not started	No	No	No
Graduation	Unmarried	Hinduism	No	Yes	Not started	No	No	No
Graduation	Unmarried	Islam	Yes	No	Not started	Yes	No	No
Higher sec	Married	Islam	No	No	Both doses	No	No	Yes
Higher sec	Married	Islam	No	No	Both doses	No	No	Yes
Higher sec	Married	Islam	No	No	Not started	No	No	Yes
Graduation	Unmarried	Islam	No	No	Not started	No	No	Yes
Higher sec	Unmarried	Islam	No	No	Not started	Yes	No	No
Graduation	Unmarried	Islam	No	No	Both doses	No	No	No
Higher sec	Unmarried	Islam	No	No	Not started	No	Yes	No
Graduation	Unmarried	Hinduism	Yes	No	Both doses	No	No	No
Post-gradu	Married	Islam	Yes	Yes	Both doses	Yes	No	No
Graduation	Married	Islam	Yes	No	Both doses	Yes	No	No
Post-gradu	Married	Islam	Yes	No	Both doses	No	No	No
Higher sec	Unmarried	Islam	No	No	Not started	No	No	No
Graduation	Married	Islam	Yes	No	Both doses	No	No	No
Graduation	Unmarried	Islam	No	No	Both doses	Yes	No	Yes
Post-gradu	Married	Islam	Yes	No	Both doses	No	No	No
Higher sec	Unmarried	Islam	No	Yes	Not started	No	No	No
Post-gradu	Unmarried	Islam	Yes	No	Both doses	No	No	No
Post-gradu	Married	Hinduism	Yes	No	Both doses	No	No	No
Graduation	Married	Islam	Yes	No	Both doses	No	No	No
Post-gradu	Married	Islam	Yes	No	Both doses	No	No	No
Post-gradu	Married	Islam	Yes	No	Both doses	No	No	No
Post-gradu	Married	Islam	No	No	Both doses	No	No	Yes
Post-gradu	Married	Islam	Yes	Yes	Both doses	Yes	No	No
Graduation	Unmarried	Islam	Yes	No	Both doses	No	No	No
Graduation	Married	Islam	Yes	No	Both doses	No	No	No
Post-gradu	Married	Islam	Yes	No	Both doses	No	No	No
Post-gradu	Married	Hinduism	Yes	No	Both doses	No	No	No
Higher sec	Unmarried	Islam	No	No	Not started	No	No	No
Higher sec	Unmarried	Islam	No	No	Not started	No	No	No
Graduation	Unmarried	Islam	Yes	No	Not started	Yes	No	No
Higher sec	Unmarried	Islam	No	Yes	Both doses	Yes	No	No
Higher sec	Married	Islam	No	No	Both doses	No	No	No
Post-gradu	Married	Islam	Yes	No	Both doses	No	No	No
Graduation	Married	Islam	No	No	Both doses	No	No	No
Higher sec	Unmarried	Islam	Yes	No	Not started	Yes	No	No
Graduation	Unmarried	Islam	No	No	Both doses	No	No	Yes

Graduation	Unmarried	Islam	Yes	No	Not started	Yes	No	No
Graduation	Unmarried	Islam	Yes	No	Not started	Yes	No	No
Graduation	Unmarried	Islam	No	No	Only 1st dc	No	No	No
Graduation	Unmarried	Islam	No	Yes	Only 1st dc	No	No	Yes
Graduation	Unmarried	Hinduism	No	Yes	Both doses	Yes	No	No
Higher sec	Unmarried	Islam	No	No	Not started	No	No	No
Post-gradu	Married	Hinduism	No	Yes	Both doses	Yes	No	No
Higher sec	Married	Hinduism	No	No	Not started	No	No	No
Graduation	Married	Islam	Yes	Yes	Only 1st dc	No	Yes	No
Graduation	Unmarried	Islam	Yes	Yes	Only 1st dc	No	No	Yes
Graduation	Unmarried	Islam	No	Yes	Only 1st dc	Yes	No	No
Graduation	Unmarried	Islam	No	Yes	Only 1st dc	No	No	No
Graduation	Unmarried	Islam	Yes	Yes	Both doses	No	No	No
Graduation	Unmarried	Islam	No	No	Only 1st dc	No	No	No
Graduation	Unmarried	Hinduism	Yes	No	Not started	Yes	No	No
Graduation	Married	Hinduism	No	No	Not started	Yes	No	No
Graduation	Married	Hinduism	No	Yes	Not started	No	No	No
Graduation	Unmarried	Islam	Yes	No	Both doses	Yes	No	No
Post-gradu	Married	Islam	No	Yes	Not started	No	No	No
Graduation	Married	Islam	No	No	Not started	No	No	No
Post-gradu	Unmarried	Islam	Yes	No	Both doses	Yes	No	No
Higher sec	Unmarried	Islam	No	No	Not started	Yes	Yes	No
Post-gradu	Married	Islam	Yes	Yes	Both doses	Yes	Yes	No
Post-gradu	Unmarried	Hinduism	Yes	Yes	Both doses	No	No	No
Graduation	Unmarried	Islam	No	No	Not started	Yes	No	No
Higher sec	Unmarried	Buddhism	No	No	Not started	No	No	No
Graduation	Married	Hinduism	Yes	Yes	Only 1st dc	No	No	No
Graduation	Unmarried	Hinduism	Yes	Yes	Both doses	Yes	No	No
Graduation	Unmarried	Islam	No	No	Only 1st dc	No	No	No
Graduation	Unmarried	Buddhism	Yes	No	Both doses	No	No	No
Graduation	Unmarried	Buddhism	No	No	Both doses	No	No	No
Post-gradu	Married	Buddhism	No	Yes	Both doses	Yes	Yes	No
Post-gradu	Married	Buddhism	No	Yes	Both doses	Yes	Yes	No
Higher sec	Unmarried	Buddhism	No	No	Not started	Yes	No	No
Higher sec	Married	Buddhism	Yes	No	Both doses	No	No	No
Post-gradu	Unmarried	Buddhism	Yes	Yes	Both doses	No	Yes	No
Graduation	Unmarried	Buddhism	No	No	Both doses	Yes	No	No
Graduation	Unmarried	Islam	No	No	Both doses	No	No	No
Higher sec	Unmarried	Buddhism	No	No	Not started	Yes	No	No
Graduation	Unmarried	Hinduism	No	No	Both doses	Yes	No	No
Graduation	Unmarried	Buddhism	Yes	No	Both doses	No	No	No
Graduation	Unmarried	Hinduism	No	No	Not started	No	No	No
Higher sec	Unmarried	Buddhism	Yes	No	Not started	No	No	No
Higher sec	Unmarried	Islam	No	No	Both doses	No	No	No
Post-gradu	Married	Hinduism	No	No	Both doses	No	No	No
Higher sec	Unmarried	Buddhism	Yes	Yes	Not started	No	No	No
Graduation	Unmarried	Islam	No	No	Not started	No	No	No
Higher sec	Unmarried	Buddhism	No	Yes	Not started	Yes	No	No
Graduation	Unmarried	Islam	No	No	Only 1st dc	No	No	No
Graduation	Married	Buddhism	Yes	No	Both doses	No	No	No
Graduation	Unmarried	Buddhism	No	No	Both doses	No	Yes	Yes
Higher sec	Unmarried	Hinduism	No	No	Not started	Yes	No	No

Graduation	Married	Buddhism	Yes	No	Both doses	Yes	No	No
Higher sec	Unmarried	Islam	No	No	Only 1st dc	Yes	No	No
Graduation	Unmarried	Hinduism	Yes	No	Both doses	No	No	No
Graduation	Unmarried	Islam	No	Yes	Only 1st dc	No	No	No
Graduation	Unmarried	Islam	No	No	Both doses	Yes	No	No
Graduation	Unmarried	Islam	No	No	Both doses	No	No	No
Post-gradu	Unmarried	Hinduism	Yes	No	Only 1st dc	Yes	No	No
Graduation	Married	Islam	No	No	Both doses	No	No	No
Graduation	Married	Islam	No	No	Both doses	No	No	No
Graduation	Married	Islam	Yes	No	Both doses	No	No	No
Graduation	Unmarried	Hinduism	No	No	Both doses	Yes	No	No
Graduation	Married	Islam	No	No	Only 1st dc	Yes	No	No
Higher sec	Unmarried	Islam	No	No	Not started	No	No	No
Higher sec	Unmarried	Islam	No	No	Only 1st dc	No	No	No
Higher sec	Married	Islam	No	No	Only 1st dc	Yes	No	No
Graduation	Unmarried	Islam	Yes	No	Only 1st dc	No	No	No
Graduation	Unmarried	Islam	Yes	Yes	Not started	No	No	No
Graduation	Unmarried	Islam	No	No	Both doses	Yes	No	No
Graduation	Unmarried	Islam	No	No	Both doses	No	Yes	No
Graduation	Unmarried	Hinduism	No	No	Not started	Yes	No	No
Graduation	Unmarried	Buddhism	No	No	Both doses	Yes	Yes	Yes
Higher sec	Unmarried	Islam	No	No	Not started	No	No	No
Graduation	Married	Islam	Yes	Yes	Both doses	No	No	No
Graduation	Married	Islam	No	Yes	Both doses	No	No	No
Graduation	Married	Islam	No	No	Both doses	No	No	No
Graduation	Married	Islam	No	No	Both doses	No	No	No
Graduation	Married	Islam	Yes	No	Both doses	No	No	No
Graduation	Unmarried	Islam	Yes	Yes	Both doses	Yes	No	Yes
Higher sec	Married	Islam	No	No	Both doses	No	No	Yes
Higher sec	Married	Islam	No	No	Both doses	No	No	No
Graduation	Married	Islam	Yes	No	Both doses	No	No	No
Graduation	Married	Islam	No	No	Both doses	No	No	No
Graduation	Married	Islam	No	No	Both doses	No	No	No
Higher sec	Married	Islam	No	No	Both doses	Yes	No	No
Graduation	Married	Islam	No	No	Both doses	No	No	No
Higher sec	Married	Islam	No	No	Both doses	No	No	No
Graduation	Married	Islam	No	No	Both doses	No	No	No
Graduation	Married	Islam	No	No	Both doses	No	No	No
Graduation	Unmarried	Islam	No	No	Both doses	Yes	No	No
Higher sec	Unmarried	Islam	No	No	Not started	No	No	No
Higher sec	Married	Islam	No	No	Not started	No	No	No
Higher sec	Married	Islam	No	No	Only 1st dc	Yes	No	No
Uneducate	Married	Islam	No	No	Not started	No	No	No
Uneducate	Married	Islam	No	No	Both doses	No	No	No
Higher sec	Unmarried	Islam	No	No	Not started	No	No	No
Higher sec	Married	Islam	No	No	Both doses	No	No	No
Uneducate	Married	Islam	No	No	Both doses	No	No	No
Uneducate	Married	Islam	No	No	Not started	No	No	No
Uneducate	Married	Islam	No	No	Not started	No	No	No
Uneducate	Married	Islam	No	No	Not started	No	No	No
Graduation	Unmarried	Islam	No	No	Both doses	No	No	No
Graduation	Unmarried	Islam	No	No	Both doses	No	No	No

Uneducate	Married	Islam	Yes	No	Not started	No	No	No
Uneducate	Married	Islam	Yes	No	Not started	No	No	No
Higher sec	Married	Islam	No	No	Not started	No	No	No
Higher sec	Married	Islam	No	No	Not started	Yes	No	No
Higher sec	Married	Islam	No	No	Only 1st dc	No	No	No
Higher sec	Married	Islam	No	No	Only 1st dc	No	Yes	No
Uneducate	Married	Islam	No	Yes	Not started	Yes	Yes	No
Higher sec	Married	Islam	No	Yes	Not started	No	No	No
Higher sec	Married	Islam	No	No	Not started	No	Yes	No
Higher sec	Married	Islam	No	No	Not started	No	No	No
Higher sec	Married	Islam	No	Yes	Not started	No	No	Yes
Higher sec	Married	Islam	No	No	Not started	Yes	Yes	No
Higher sec	Married	Islam	No	No	Not started	No	Yes	No
Higher sec	Married	Islam	No	No	Not started	Yes	No	No
Higher sec	Married	Islam	No	No	Not started	No	Yes	No
Uneducate	Married	Islam	No	No	Not started	No	No	No
Higher sec	Married	Islam	No	No	Not started	No	No	No
Higher sec	Married	Islam	No	No	Not started	No	Yes	No
Higher sec	Married	Islam	No	No	Only 1st dc	No	No	No
Higher sec	Married	Islam	No	Yes	Not started	No	Yes	No
Higher sec	Unmarried	Islam	No	No	Not started	No	No	No
Higher sec	Married	Islam	No	No	Not started	No	No	No
Uneducate	Married	Islam	No	No	Not started	Yes	No	No
Higher sec	Married	Islam	Yes	Yes	Both doses	Yes	No	No

acne	crack_skin	color_skin	press_nose	press_ear	rash	burn_skin	itch	hairfall
No	No	No	No	No	No	No	No	No
No	No	No	No	No	No	No	No	No
No	No	No	No	No	No	No	No	No
No	No	No	No	No	No	No	No	No
No	No	No	No	No	No	No	No	No
Yes	Yes	Yes	No	No	No	No	No	No
Yes	No	No	No	No	No	No	No	Yes
No	No	No	No	No	No	No	No	No
Yes	No	No	No	No	No	No	No	No
No	No	No	No	No	No	No	No	No
No	No	No	No	No	No	No	No	No
Yes	No	No	No	No	Yes	No	No	No
No	Yes	No	Yes	Yes	No	No	No	No
No	No	No	No	No	No	No	No	No
No	No	No	No	No	No	No	No	No
No	Yes	No	No	No	No	Yes	No	No
No	No	No	No	Yes	Yes	Yes	Yes	No
No	No	No	No	No	No	No	No	No
No	No	No	No	No	No	No	No	No
No	No	No	No	Yes	Yes	No	Yes	No
No	No	No	No	No	No	No	No	No
No	No	No	No	No	No	No	No	No
No	No	No	No	No	No	No	No	No
No	No	No	No	No	No	No	No	No
No	No	No	No	No	Yes	No	No	No
No	No	No	No	No	No	No	No	No
Yes	No	No	No	No	No	No	No	No
Yes	Yes	No	No	No	Yes	No	Yes	No
Yes	No	No	No	No	No	No	Yes	No
No	No	No	No	No	No	No	No	No
No	No	No	No	Yes	No	No	No	No
No	No	No	No	Yes	No	No	No	No
No	No	No	No	No	No	No	No	No
Yes	No	No	No	No	No	No	No	No
No	No	No	No	No	No	No	No	No
Yes	No	No	No	No	No	No	No	No
No	No	No	No	No	No	No	No	Yes
No	No	No	No	Yes	No	No	No	No
Yes	No	No	No	No	No	No	Yes	No
No	No	No	No	No	No	No	No	No
No	No	No	No	No	No	No	No	No
Yes	No	No	No	No	Yes	No	No	No
No	No	No	No	No	No	No	No	No
No	No	No	No	No	No	No	No	No
Yes	No	No	No	No	No	No	No	No
No	No	No	No	No	Yes	No	Yes	No
No	No	No	No	No	No	No	No	No
No	No	No	No	No	No	No	Yes	No
No	No	No	No	No	No	No	No	No
No	No	No	No	No	No	No	Yes	No
No	No	No	No	No	No	No	No	No

No	No	No	No	No	No	No	No	No
No	Yes	No	No	No	No	No	No	No
No	No	No	No	No	No	No	No	No
Yes	No	No	No	No	No	No	No	No
Yes	No	No	Yes	No	No	Yes	Yes	No
No	No	No	No	No	No	No	No	No
No	No	No	No	No	No	No	No	No
No	No	No	No	No	No	No	No	No
No	Yes	No	No	No	Yes	Yes	Yes	Yes
No	No	No	No	No	No	No	No	No
Yes	No	No	No	No	Yes	No	No	No
Yes	No	No	No	Yes	No	Yes	Yes	No
No	No	No	No	No	No	No	No	No
No	No	No	No	No	No	No	No	No
No	No	No	No	No	No	No	No	No
No	No	No	No	No	No	No	No	No
No	No	No	No	No	No	No	No	No
No	Yes	No	No	No	Yes	No	Yes	No
No	No	No	No	No	No	No	No	No
No	No	No	No	No	No	No	Yes	No
No	No	No	No	No	Yes	No	Yes	No
Yes	No	No	No	No	No	No	No	No
No	No	No	No	No	No	No	No	No
No	No	No	No	No	Yes	No	No	No
No	No	No	No	No	No	No	No	No
No	No	No	No	No	No	No	No	No
No	No	No	No	No	No	No	Yes	No
No	No	No	No	No	No	No	No	No
Yes	No	No	No	Yes	No	Yes	No	No
No	No	No	No	No	No	No	No	No
No	No	No	No	Yes	No	No	No	No
No	No	No	No	No	No	No	No	No
No	No	No	No	No	No	No	No	Yes
No	No	No	No	No	No	No	No	No
No	No	No	No	No	No	No	No	No
Yes	No	No	No	Yes	Yes	No	Yes	No
No	No	No	Yes	Yes	No	No	No	No
No	No	No	No	No	No	No	No	No
No	No	No	No	No	No	No	No	No
No	No	No	No	No	No	No	No	No
No	No	No	No	No	No	No	No	No
No	No	No	No	No	No	No	No	No
No	No	No	No	No	No	No	No	No
No	No	No	No	No	No	No	No	No
No	No	No	No	No	No	No	No	No
No	No	No	No	No	No	No	No	No
Yes	No	No	No	No	Yes	Yes	Yes	No
No	No	No	No	No	No	No	No	No
No	No	No	No	No	No	No	No	No
No	No	No	No	No	No	No	No	No
No	No	No	No	Yes	No	No	No	No
No	No	No	No	No	No	No	No	No

No	No	No	No	No	No	No	No	No
No	No	No	No	No	No	No	No	No
No	No	No	No	No	No	No	No	No
Yes	No	No	No	No	Yes	No	Yes	No
No	No	No	No	Yes	No	Yes	No	No
No	No	No	No	No	No	Yes	No	No
No	No	No	No	No	No	No	No	Yes
No	Yes	No	No	No	No	No	Yes	No
No	No	No	No	No	No	No	No	No
No	No	No	No	No	No	No	No	No
No	No	No	No	Yes	No	No	No	No
No	No	No	No	No	No	No	No	No
No	No	No	No	No	No	No	Yes	No
No	No	No	No	No	No	No	No	No
No	No	No	Yes	Yes	No	No	No	Yes
Yes	No	No	No	No	No	No	Yes	Yes
No	No	No	No	No	No	No	No	No
No	No	No	No	No	No	No	No	No
No	No	No	No	No	No	No	No	No
No	No	No	No	No	No	No	No	No
No	No	No	No	No	No	No	No	No
Yes	No	No	No	No	No	No	No	No
No	No	No	No	Yes	No	No	No	No
No	No	No	No	No	No	No	No	No
No	No	No	No	No	No	No	No	No
No	No	No	No	No	No	No	No	No
Yes	No	No	No	No	No	No	No	Yes
No	No	No	No	No	No	No	No	No
Yes	No	No	No	Yes	No	No	No	No
No	No	No	No	No	No	No	No	No
No	No	No	No	No	No	No	No	No
No	No	No	No	No	No	No	No	No
No	Yes	No	Yes	Yes	Yes	No	No	Yes
No	No	No	No	No	No	No	No	No
No	No	No	No	No	No	No	No	No
No	No	No	No	No	No	No	No	No
No	No	No	No	No	No	No	No	No
No	No	No	No	No	No	No	No	No
No	No	No	No	No	No	No	No	No
No	No	No	No	No	Yes	No	Yes	No
No	No	No	No	No	No	No	No	No
No	No	No	Yes	No	No	No	No	No
No	No	No	No	No	No	No	No	No
No	No	No	No	No	No	No	No	No
No	No	No	No	No	No	No	No	No
No	No	No	No	No	No	No	No	No
No	No	No	No	No	No	No	No	No
No	No	No	No	No	No	No	No	No
No	No	No	No	No	No	No	No	No
No	No	No	No	No	No	No	No	No
No	No	No	No	No	No	No	No	No
Yes	No	No	No	No	No	No	No	No

No	No	No	No	No	No	No	No	No
No	No	No	No	No	No	No	No	No
No	No	No	No	No	No	No	No	No
No	No	No	No	No	No	No	No	Yes
No	No	No	No	No	No	No	No	No
No	No	No	No	No	No	No	No	No
Yes	No	No	No	No	No	Yes	Yes	No
No	No	No	No	No	No	No	No	No
No	No	No	Yes	Yes	No	No	No	No
No	No	No	No	Yes	No	No	No	No
Yes	Yes	No	Yes	Yes	Yes	Yes	Yes	Yes
No	No	No	No	No	No	No	No	No
No	No	No	No	No	No	No	No	No
No	No	No	No	No	No	No	No	No
Yes	Yes	Yes	No	No	No	No	No	No
No	No	No	No	No	No	No	No	No
No	No	No	No	No	No	No	No	No
No	No	No	No	No	No	No	No	No
No	No	No	No	Yes	No	No	No	No
Yes	No	No	No	No	No	No	No	No
No	No	No	No	Yes	No	No	Yes	Yes
No	No	No	No	No	No	No	No	No
No	No	No	No	Yes	No	No	No	No
Yes	No	No	No	No	No	No	No	No
No	No	No	No	No	No	No	No	No
No	No	No	No	No	No	No	No	No
Yes	No	No	No	No	No	No	No	No
No	No	No	No	No	No	No	No	No
Yes	No	No	No	No	No	No	No	No
No	No	No	No	No	No	No	No	No
No	No	No	No	No	No	No	No	No
Yes	No	No	No	Yes	No	No	Yes	No
No	Yes	No	No	Yes	No	No	No	No
No	No	No	No	No	No	No	No	Yes
Yes	No	No	No	Yes	No	Yes	Yes	No
No	No	No	No	No	No	No	No	No
No	No	No	No	No	No	No	No	No
No	No	No	No	No	No	No	No	No
Yes	No	No	No	Yes	No	No	No	No
No	No	No	No	No	No	No	No	No
No	No	No	No	No	No	No	No	No
Yes	No	No	No	No	No	No	No	No
No	No	No	No	No	No	No	No	No
No	No	No	No	No	No	No	No	No
Yes	No	No	No	Yes	No	No	No	No
No	No	No	No	No	No	No	No	No
No	No	No	No	No	No	No	No	No
No	No	No	No	No	No	No	No	No
No	No	No	No	No	No	No	No	No
No	No	No	No	No	No	No	No	No
No	No	No	No	No	No	No	No	No
No	No	No	No	No	No	No	No	No

Yes	No	No	No	No	Yes	No	Yes	Yes
No	No	No	No	No	No	No	No	No
No	No	No	No	No	Yes	No	No	No
No	No	No	No	No	No	No	No	No
Yes	No	No	No	No	Yes	No	No	No
Yes	No	No	No	No	No	Yes	Yes	No
No	No	No	Yes	Yes	No	No	No	No
No	No	No	No	No	No	No	No	No
Yes	No	No	No	No	Yes	No	No	No
No	No	No	No	No	No	Yes	No	No
No	No	No	No	No	No	No	No	No
No	No	No	No	No	No	No	No	No
No	No	No	No	No	No	No	No	No
Yes	No	No	No	No	No	No	No	No
No	No	No	No	No	No	No	No	No
No	No	No	No	No	No	Yes	No	No
No	No	No	No	No	No	No	No	No
Yes	No	No	No	No	No	No	No	No
Yes	No	No	No	Yes	Yes	No	No	No
Yes	No	No	No	No	Yes	No	Yes	No
No	No	No	No	No	No	No	No	No
No	No	No	No	No	No	No	No	No
No	No	No	No	No	No	No	No	No
No	No	No	No	No	No	No	No	No
No	No	No	No	Yes	No	No	No	No
No	No	No	No	No	No	No	No	No
No	No	No	No	No	No	No	No	No
No	No	No	No	No	No	No	No	No
No	No	No	No	No	No	No	No	No
Yes	No	No	No	No	Yes	Yes	Yes	No
No	No	No	No	No	No	No	No	No
No	No	Yes	No	No	No	No	No	Yes
No	No	No	No	No	No	No	No	No
No	No	No	No	Yes	No	No	No	No
No	No	No	No	No	No	No	No	No
Yes	No	No	No	No	No	No	No	No
No	No	No	No	No	No	No	No	No
No	No	No	No	No	No	No	No	No
No	No	No	Yes	No	No	No	Yes	No
No	No	No	Yes	No	No	No	No	No
Yes	No	No	No	No	No	No	No	No
No	No	No	No	No	No	No	No	No
No	No	No	No	No	No	No	No	No
No	No	No	Yes	Yes	No	No	Yes	No
No	No	No	Yes	Yes	No	No	No	No
No	No	No	No	No	No	No	No	No
Yes	No	No	No	No	No	No	No	No
No	No	No	No	No	No	No	No	No
Yes	No	Yes	No	No	Yes	No	No	Yes
No	No	No	Yes	Yes	No	No	No	No
No	No	No	Yes	No	No	No	No	No
No	No	No	No	No	No	No	No	No

No	No	No	No	No	No	No	No	No
No	No	No	No	No	No	No	No	No
No	No	No	No	No	No	No	No	No
No	No	No	No	No	No	No	No	No
Yes	No	No	No	No	No	No	Yes	No
Yes	No	No	No	No	No	No	No	No
No	No	No	No	Yes	No	Yes	No	No
No	No	No	No	No	No	No	No	No
No	No	No	No	No	No	No	No	No
No	No	No	No	No	No	No	No	No
No	No	No	No	No	No	No	No	No
No	No	No	No	No	No	No	No	No
No	No	No	No	No	No	No	No	No
Yes	No	No	No	No	No	No	No	No
No	No	No	No	No	Yes	No	No	No
No	No	No	No	No	No	No	No	No
No	No	No	No	No	No	No	No	No
No	No	No	Yes	No	No	Yes	Yes	No
No	No	No	Yes	Yes	No	Yes	No	No
No	Yes	No	No	Yes	No	No	No	No
No	No	No	No	Yes	No	No	No	No
No	No	No	No	No	No	No	No	No
Yes	No	No	No	No	No	No	No	No
No	No	No	No	No	No	No	No	No
No	No	No	No	No	No	No	No	No
No	No	No	No	No	No	No	No	No
No	No	No	No	No	No	No	No	No
No	No	No	No	No	No	No	No	No
No	No	No	No	No	No	No	No	No
No	No	No	No	No	No	No	No	No
No	No	No	No	No	No	Yes	No	Yes
Yes	No	No	No	No	No	No	No	No
No	No	No	No	No	No	No	No	No
No	No	No	No	No	No	No	No	No
No	No	No	No	No	No	No	No	No
No	No	No	No	No	No	No	Yes	No
No	No	No	No	Yes	No	No	Yes	No
No	No	No	No	No	No	Yes	No	No
No	No	No	No	No	No	No	No	No
No	No	No	No	No	No	No	No	No
No	No	No	No	No	No	No	No	No
No	No	No	No	No	No	No	No	No
No	No	No	No	Yes	No	No	No	No
No	No	No	No	No	No	No	No	No
No	No	No	No	No	No	No	No	No
No	No	No	No	No	No	No	No	No
No	No	No	No	No	No	No	No	No
No	No	No	No	No	No	No	Yes	Yes
No	No	No	No	No	No	No	No	No
No	No	No	No	No	No	No	No	No
Yes	No	No	No	No	No	No	No	Yes
No	No	No	No	No	No	No	No	No

No	No	No	No	No	No	No	Yes	No
No	No	No	No	No	No	No	No	No
No	No	No	No	No	No	No	No	No
No	No	No	No	No	No	No	No	No
No	No	No	No	No	No	No	No	No
No	No	No	No	No	No	No	No	No
No	No	No	No	Yes	No	No	No	No
No	No	No	No	No	No	No	No	No
No	No	No	No	No	No	No	No	No
No	No	No	No	No	No	No	No	No
No	No	No	No	No	No	No	No	No
No	No	No	No	No	No	No	No	No
No	No	No	No	No	No	No	No	No
No	No	No	No	No	No	No	No	No
Yes	Yes	No	No	No	Yes	No	Yes	Yes
No	No	No	No	No	No	No	No	No
No	No	No	No	No	No	No	No	No
No	No	No	No	Yes	No	No	No	No
Yes	No	No	No	No	No	No	Yes	No
No	No	No	No	No	No	No	No	No
No	No	No	No	No	No	No	No	No
Yes	No	No	No	No	No	No	No	No
Yes	No	Yes	No	No	No	No	Yes	Yes
Yes	No	No	No	No	No	No	Yes	No
No	No	No	No	No	No	No	No	No
No	No	No	No	No	No	No	No	No
Yes	No	No	No	No	No	No	No	No
No	No	No	No	No	No	No	No	No
No	No	No	No	No	No	No	No	No
No	No	No	No	No	No	No	No	No
No	No	No	No	No	No	No	No	No
No	No	No	No	No	No	No	Yes	No
Yes	No	No	No	No	No	No	No	No
No	No	No	No	No	No	No	No	No
No	No	No	No	No	No	No	Yes	No
No	No	No	No	No	No	No	No	No
No	No	No	No	No	No	No	No	No
No	No	No	No	No	No	No	No	No
No	No	No	No	No	No	No	No	Yes
No	No	No	No	No	No	No	No	No
No	No	No	No	No	No	No	No	No
Yes	No	No	No	No	No	No	Yes	No
No	No	No	No	Yes	No	No	No	Yes
Yes	Yes	No	No	No	Yes	No	Yes	No
No	No	No	No	No	No	No	No	No
Yes	No	No	No	No	Yes	No	No	No
No	No	No	No	No	No	No	No	No
No	No	No	No	No	No	No	Yes	No
No	No	No	No	No	No	No	No	No
No	No	No	No	Yes	No	No	No	No
No	Yes	No	No	No	No	No	No	No
No	No	No	No	No	No	No	No	No

No	No	No	No	No	No	No	No	No
No	No	No	No	No	No	No	No	No
No	No	No	No	No	No	No	No	No
Yes	No	No	No	No	No	No	No	No
No	No	No	No	No	No	No	No	No
Yes	No	No	Yes	Yes	Yes	No	Yes	No
No	No	No	No	Yes	No	No	No	No
No	No	No	No	No	No	No	Yes	No
No	No	No	No	No	No	No	No	No
No	No	No	No	No	No	No	No	No
No	No	No	No	No	No	No	No	No
No	No	No	No	No	No	No	No	No
No	No	No	No	No	No	No	No	No
No	No	No	No	No	No	No	No	No
No	No	No	No	No	No	No	No	No
No	No	No	No	No	No	No	No	No
No	No	No	No	No	No	No	No	No
No	No	No	No	No	No	No	No	No
No	No	No	No	No	No	No	No	No
No	No	No	No	No	No	No	No	No
No	No	No	No	No	No	No	No	No
No	No	No	No	No	No	No	No	No
No	Yes	No	No	No	No	Yes	No	No
No	No	No	No	Yes	No	No	No	No
No	No	No	No	No	No	No	No	No
No	No	No	No	Yes	No	No	No	No
No	No	No	No	Yes	No	No	Yes	Yes
No	No	No	No	No	No	No	No	No
No	No	No	No	No	No	No	No	No
No	No	No	No	No	No	No	No	No
No	No	No	No	No	No	No	No	No
No	No	No	No	No	No	No	No	No
Yes	No	No	No	No	No	No	No	No
No	No	No	No	No	No	No	No	No
No	No	No	No	No	No	No	No	No
No	No	No	No	No	No	No	No	No
No	No	No	No	Yes	No	No	No	No
No	No	No	No	No	No	No	No	No
No	No	No	No	No	No	No	No	No
Yes	Yes	No	Yes	Yes	Yes	No	No	No
Yes	No	No	No	No	No	No	Yes	No
No	No	No	No	Yes	No	No	No	No
No	No	No	No	No	No	No	No	No
No	Yes	No	No	No	No	No	No	No
No	No	No	No	No	No	No	No	No
No	No	No	No	No	No	No	No	No
No	No	No	No	No	No	No	No	No
No	No	No	Yes	No	No	No	No	No
No	No	No	No	No	No	No	No	No
No	No	No	No	No	No	No	No	No
No	No	No	No	No	No	No	No	No
No	No	No	No	No	No	No	No	No

No	No	No	No	No	No	No	No	No
No	No	No	Yes	No	No	No	No	No
No	No	No	No	No	No	No	No	No
No	No	No	No	No	Yes	No	No	No
No	No	No	No	No	No	No	No	No
No	No	No	No	No	No	No	No	No
No	No	No	No	No	Yes	No	Yes	No
No	No	No	No	No	No	No	Yes	No
No	No	No	Yes	Yes	No	No	No	No
No	No	No	No	Yes	No	No	No	No
No	No	No	No	No	No	No	No	No
No	No	No	No	No	No	No	No	Yes
No	No	No	No	No	No	No	No	No
No	No	No	No	No	No	No	No	No
No	No	No	No	No	No	No	No	No
No	No	No	No	No	Yes	No	No	No
No	No	No	No	No	No	No	No	No
Yes	Yes	No	No	No	No	No	No	No
No	No	No	No	No	No	No	No	No
No	No	No	No	No	No	No	No	No
No	No	No	Yes	Yes	No	No	No	No
Yes	No	No	No	No	No	No	No	No
No	No	No	No	No	No	No	No	No
No	No	No	No	No	No	No	No	No
No	No	No	No	No	No	No	Yes	No
No	No	No	No	No	No	No	No	No
No	No	No	No	No	No	No	No	No
Yes	No	No	No	Yes	No	No	No	No
Yes	No	No	No	No	No	No	No	No
No	No	No	No	No	No	No	No	No
No	No	No	No	No	No	No	No	No
No	No	No	No	No	No	No	No	No
No	No	No	No	No	No	No	Yes	No
No	No	No	No	No	No	No	No	No
Yes	No	No	No	No	No	No	Yes	No
No	No	No	No	No	No	No	No	No
No	No	No	No	Yes	No	No	Yes	No
No	No	No	No	No	No	No	No	No
No	No	No	No	No	Yes	No	No	No
No	No	No	No	No	No	No	No	No
No	No	No	No	No	No	No	Yes	No
No	No	No	No	No	No	No	No	No
No	No	No	No	No	No	No	No	No
Yes	No	No	No	No	No	Yes	No	No
No	No	No	No	No	No	No	No	No
No	No	No	No	No	No	No	No	Yes
No	No	Yes	No	No	No	No	No	No
No	No	No	No	No	No	No	No	No
Yes	No	No	No	No	No	Yes	No	No
No	No	No	No	No	No	Yes	No	No
No	No	No	No	No	No	No	No	No
No	No	No	No	No	No	No	No	No

No	No	No	No	No	No	No	No	No
No	No	No	No	No	No	No	No	No
No	No	No	Yes	Yes	No	No	No	No
Yes	No	No	Yes	No	No	No	No	No
No	No	No	No	No	No	No	No	No
No	No	No	No	No	No	No	No	No
No	No	No	No	No	No	No	No	No
No	No	No	No	No	No	No	No	No
No	No	No	No	No	No	No	No	No
No	No	No	No	No	No	No	No	No
No	No	No	No	No	No	No	No	No
No	No	No	No	No	No	No	No	No
No	No	Yes	No	No	No	No	No	No
No	No	No	No	No	No	No	No	No
Yes	No	No	Yes	No	No	Yes	No	No
No	No	No	No	No	No	No	No	No
No	No	No	Yes	No	No	No	No	No
No	No	No	No	No	No	No	No	No
No	No	No	No	No	No	No	No	No
No	No	No	No	No	No	No	No	No
No	No	No	No	No	No	No	No	No
No	No	No	No	No	No	No	No	No
No	No	No	No	No	No	No	No	No
No	No	No	No	No	No	No	No	No
No	No	No	No	No	No	No	No	No
No	No	No	No	No	No	No	No	No
Yes	No	No	No	No	No	No	No	No
No	No	No	No	No	No	No	Yes	Yes
No	No	No	No	Yes	No	No	No	No
No	No	No	No	No	No	No	No	No
No	No	No	No	No	No	No	No	No
No	No	No	No	No	Yes	No	Yes	No
No	No	Yes	Yes	Yes	No	No	No	No
No	No	No	No	Yes	No	Yes	No	No
No	No	Yes	Yes	No	No	No	No	No
No	No	No	No	No	No	No	No	No
No	No	No	No	No	No	No	No	No
No	No	No	No	Yes	No	No	No	No
No	No	No	No	No	No	No	No	No
No	No	Yes	No	No	No	No	Yes	No
No	No	No	No	No	No	No	No	No
No	No	No	No	No	No	No	No	No
No	No	No	No	No	No	No	No	No
No	No	No	No	No	No	No	No	No
No	No	No	No	No	No	No	No	No
No	No	No	No	No	No	No	No	No
No	No	No	No	No	No	No	No	No
No	No	No	No	No	No	No	No	No
No	No	No	No	No	No	No	No	No
No	No	No	Yes	No	No	No	No	No
Yes	No	No	No	Yes	No	No	No	No
No	No	No	No	Yes	No	No	No	No

No	No	No	No	No	No	No	No	No
No	No	No	No	No	No	No	No	No
No	No	No	No	No	No	No	No	No
No	No	No	No	No	No	No	No	No
No	No	No	No	No	No	No	No	No
No	No	No	No	No	No	No	No	No
Yes	No	No	No	No	No	No	No	No
No	No	No	No	No	No	No	Yes	No
No	Yes	No	Yes	No	No	No	No	No
No	No	No	No	No	No	No	No	No
No	No	No	No	No	No	No	No	No
No	No	No	No	Yes	No	No	No	No
No	No	No	No	No	Yes	No	Yes	No
No	No	No	No	No	No	No	No	No
No	No	No	No	No	No	No	No	No
No	No	No	No	No	No	No	No	No
No	No	No	No	No	No	No	No	No
No	No	No	No	No	No	No	No	No
No	No	No	No	No	No	No	No	No
No	No	No	No	No	No	No	No	No
Yes	No	No	No	No	No	No	Yes	No
No	No	No	No	No	No	No	No	No
No	No	No	No	No	No	No	No	No
No	No	No	No	No	No	No	No	No
No	No	No	No	No	No	No	No	No
Yes	No	No	No	No	No	No	No	No
No	No	No	No	No	No	No	No	No
No	No	No	No	No	Yes	No	Yes	No
No	No	No	Yes	No	No	No	No	No
No	No	No	Yes	No	No	Yes	No	No
No	Yes	No	No	No	No	Yes	No	No
No	No	No	Yes	No	No	No	No	No
No	No	No	No	No	No	No	No	No
No	No	No	No	No	No	No	No	No
No	No	No	No	No	Yes	No	Yes	No
No	No	No	No	No	No	No	Yes	No
No	No	No	No	No	No	No	No	Yes
No	No	No	No	No	Yes	No	Yes	No
No	No	No	No	No	No	No	No	No
No	No	No	Yes	Yes	No	No	No	No
No	Yes	No	No	Yes	No	No	No	No
No	No	No	No	No	No	No	No	No
No	Yes	No	No	Yes	No	No	No	No
No	No	No	No	No	No	No	No	No
No	No	No	No	No	No	No	No	No
No	No	No	No	No	No	No	No	No
No	No	No	No	No	No	No	No	No
No	No	No	No	No	No	No	No	No
No	No	No	No	No	No	No	No	No
No	No	No	No	No	No	No	No	No
No	No	No	No	No	No	No	No	No
No	No	No	No	No	No	No	No	No

No	No	No	No	No	No	No	No	No
No	No	No	No	No	No	Yes	No	No
No	No	No	No	No	No	No	Yes	No
No	No	No	No	No	No	No	No	No
No	No	No	No	No	No	No	No	No
No	No	No	No	No	No	No	No	No
No	No	No	No	No	No	No	No	No
No	No	No	No	No	No	No	No	No
No	No	No	No	No	No	No	No	No
No	Yes	No	No	Yes	No	No	No	No
No	No	No	No	No	No	No	No	No
Yes	No	No	No	No	No	No	No	No
No	No	No	No	No	No	No	No	No
No	No	No	No	No	No	No	Yes	Yes
Yes	No	No	No	No	No	No	No	No
Yes	Yes	No	No	No	Yes	Yes	Yes	Yes
No	No	No	No	No	No	No	No	No
No	No	No	No	No	No	No	No	No
No	No	No	No	No	No	No	No	No
No	No	No	No	Yes	No	No	No	Yes
Yes	No	No	No	Yes	No	No	No	No
No	No	No	No	Yes	Yes	No	Yes	No
Yes	No	No	No	No	No	No	No	Yes
No	No	No	No	No	No	No	No	No
No	No	No	No	No	No	No	No	No
No	No	Yes	No	No	No	No	No	No
No	No	No	No	No	No	No	No	No
No	No	No	No	No	No	No	Yes	No
No	No	No	No	No	No	No	No	No
No	No	No	No	No	No	No	No	No
No	No	No	No	Yes	No	No	No	No
No	No	No	No	Yes	No	No	No	No
No	No	Yes	No	No	No	No	No	No
No	No	No	No	No	No	No	No	No
No	No	Yes	No	No	No	No	No	No
No	No	No	No	Yes	No	No	No	No
No	No	No	No	No	No	No	No	No
No	No	No	No	No	No	No	No	No
No	No	No	No	No	No	No	No	No
No	No	No	No	No	No	No	No	No
No	No	No	No	No	No	No	No	No
No	No	No	No	No	No	No	No	No
No	No	No	No	No	No	No	No	No
No	No	No	No	No	No	No	No	No
No	No	No	No	No	No	No	No	No
No	No	No	No	No	No	No	No	No
No	No	No	No	No	No	No	No	No
No	No	No	No	Yes	No	No	No	No
No	No	No	No	No	No	No	No	No
No	No	No	Yes	No	Yes	No	No	No
No	No	No	No	No	No	No	No	No
No	No	No	No	Yes	Yes	No	Yes	No

No	No	No	No	No	No	No	No	No
No	No	No	No	No	No	No	No	No
No	No	Yes	No	No	No	No	No	No
No	No	No	No	No	No	No	No	No
No	No	No	No	No	No	No	No	No
No	No	No	No	No	No	Yes	No	No
No	No	No	No	No	No	No	No	No
No	No	No	No	No	No	No	No	No
No	No	No	No	No	No	No	No	No
No	No	No	No	No	No	No	No	No
No	No	No	No	No	No	No	No	No
No	No	No	No	No	No	No	No	No
No	No	No	No	No	No	No	No	No
No	No	No	No	No	No	No	No	No
No	No	No	No	No	No	No	No	No
No	No	No	No	No	No	No	No	No
No	No	No	No	No	No	No	No	No
No	No	No	No	No	No	No	No	No
No	No	No	Yes	No	No	No	No	No
No	No	No	No	No	No	No	No	No
No	No	No	No	No	No	No	No	No
No	No	No	No	No	No	No	No	No
Yes	Yes	No	No	Yes	No	No	No	No
No	No	No	No	No	No	No	No	No
No	No	No	No	No	No	No	No	No
No	No	No	Yes	Yes	No	No	No	No
No	No	No	No	No	No	No	No	No
No	No	No	No	No	No	No	No	No
No	No	No	No	No	No	No	No	No
No	No	No	Yes	Yes	No	No	No	No
No	No	No	No	No	No	No	No	No
No	No	No	No	No	No	No	No	No
No	No	Yes	Yes	Yes	No	No	No	No
No	No	No	No	No	No	No	No	No
No	No	No	No	No	No	No	No	No
No	No	No	Yes	Yes	No	No	No	No
No	No	No	No	No	No	No	No	No
No	No	No	Yes	Yes	No	No	No	No
No	No	No	No	No	No	No	No	No
No	No	No	No	No	No	No	No	No
No	No	No	No	No	No	No	No	No
Yes	No	No	Yes	Yes	No	No	No	No
No	No	No	No	No	No	No	No	No
No	No	No	Yes	Yes	No	No	No	No
No	No	No	Yes	Yes	No	No	No	No
No	No	No	No	No	No	No	No	No
Yes	No	No	No	No	No	No	No	No
No	No	No	No	No	No	No	No	No
No	No	No	No	No	No	No	No	No
No	No	No	No	No	No	No	No	No
Yes	No	No	No	Yes	No	No	Yes	No
No	No	No	No	No	No	No	No	No

No	No	No	No	No	No	No	No	No
No	No	No	No	Yes	No	No	No	No
No	No	No	No	No	No	No	No	No
No	No	No	No	No	Yes	No	No	No
No	No	No	No	Yes	No	No	No	No
No	No	No	No	No	No	No	No	No
No	No	No	No	Yes	No	No	No	No
No	No	No	No	No	No	No	No	No
Yes	No	No	No	No	No	Yes	No	No
Yes	No	No	No	No	No	No	Yes	No
Yes	No	No	No	No	No	No	No	No
Yes	No	No	No	No	No	No	No	No
No	No	No	No	No	No	No	No	No
No	No	No	No	No	No	No	No	No
No	No	No	No	No	No	No	No	No
No	No	No	No	No	No	No	No	No
No	No	No	No	No	No	No	No	No
Yes	Yes	No	No	No	Yes	No	No	Yes
No	No	No	No	No	No	No	No	No
No	No	No	No	No	No	No	No	No
No	No	No	No	No	No	No	No	No
Yes	No	No	No	No	No	No	No	No
No	No	No	No	No	Yes	No	Yes	No
No	No	No	No	No	No	No	No	No
No	No	No	No	No	No	No	No	No
Yes	No	No	No	No	No	No	No	No
Yes	No	No	No	No	No	No	No	No
No	No	No	No	No	No	No	No	No
No	No	No	No	No	No	No	No	No
No	No	No	No	No	No	No	No	No
Yes	No	No	No	No	No	No	No	No
Yes	No	No	No	No	No	No	Yes	No
Yes	Yes	No	No	No	No	No	No	No
No	No	No	No	No	No	No	Yes	No
No	No	No	No	No	No	No	Yes	No
Yes	Yes	No	No	No	No	No	No	Yes
No	No	No	No	No	No	No	No	No
Yes	Yes	No	No	No	No	No	No	No
Yes	No	No	No	No	No	No	No	No
No	No	No	No	No	No	No	No	No
No	No	No	No	No	No	No	No	No
No	No	No	No	No	No	No	No	No
No	No	No	No	No	No	No	No	No
No	No	No	No	No	No	No	Yes	No
No	No	No	No	No	No	No	No	No
No	No	No	No	No	No	No	No	No
No	No	No	No	No	No	No	No	No
No	No	No	No	No	No	No	No	No
No	No	No	No	No	No	No	No	No
No	No	No	No	No	No	No	Yes	Yes
Yes	Yes	No	No	No	Yes	No	Yes	No
No	No	No	No	Yes	No	No	No	Yes

No	No	No	No	No	No	No	No	No
No	No	No	No	No	No	No	No	No
No	No	No	No	No	No	No	No	No
No	No	No	No	No	No	No	No	No
No	No	No	No	No	No	No	No	No
No	No	No	No	No	No	No	No	No
No	No	No	No	No	No	No	No	No
No	No	No	No	No	No	No	No	No
No	No	No	No	No	No	No	No	No
No	No	No	No	No	No	No	No	No
Yes	No	No	No	No	No	No	No	No
No	No	No	No	No	No	No	No	No
No	No	No	No	No	No	No	No	No
No	No	No	No	No	No	No	No	No
No	No	No	No	No	No	No	No	No
No	No	No	No	No	No	No	No	No
No	No	No	No	No	No	No	No	No
No	No	No	No	No	No	No	Yes	No
Yes	Yes	Yes	No	Yes	No	No	No	No
Yes	No	No	No	No	No	No	Yes	No
No	No	No	No	No	No	No	Yes	No
No	No	No	Yes	No	No	No	No	No
No	No	No	No	No	No	No	No	No
No	No	No	No	No	No	No	No	No
No	No	No	No	No	No	No	No	No
No	No	No	No	No	No	No	No	No
No	No	No	No	No	No	No	No	No
No	No	No	No	No	No	Yes	No	No
No	No	No	No	No	No	No	No	No
No	No	No	No	No	No	No	No	No
No	No	No	No	No	No	No	No	No
No	No	No	No	No	No	No	No	No
No	No	No	No	No	No	No	No	No
No	No	No	No	No	No	No	No	No
No	No	No	No	No	No	No	No	No
No	No	No	No	No	No	No	No	No
No	No	No	No	No	No	No	No	No
No	No	No	No	No	No	No	No	No
No	No	No	Yes	No	No	No	No	No
No	No	No	No	No	No	No	No	No
No	No	No	No	No	No	No	No	No
No	No	No	No	No	No	No	No	No
No	No	No	No	No	No	No	No	No
No	No	No	No	No	No	No	No	No
No	No	No	No	No	Yes	No	No	No
No	No	No	No	No	No	No	No	No
No	No	No	No	No	No	No	No	No
No	No	No	No	No	No	No	No	No
No	No	No	Yes	No	No	No	No	No
No	Yes	No	No	No	No	No	No	No
No	No	No	No	No	No	No	No	No
No	No	No	No	No	No	No	No	No

No	No	No	Yes	No	No	No	No	No
No	No	No	No	No	No	No	No	No
No	No	No	Yes	No	No	No	No	No
No	No	No	Yes	Yes	No	No	No	No
No	No	No	No	No	Yes	No	Yes	No
No	No	No	Yes	Yes	No	No	No	No
No	No	No	No	No	No	No	No	No
No	No	No	No	No	Yes	No	Yes	No
No	No	No	No	No	No	No	No	No
No	No	No	No	No	No	No	Yes	No
No	No	No	No	No	No	No	No	No
No	No	No	No	No	No	No	No	No
No	No	No	Yes	Yes	No	No	No	No
No	No	No	Yes	Yes	No	Yes	No	No
No	No	No	Yes	Yes	No	No	No	No
No	No	No	No	No	No	No	No	No
No	Yes	No	No	No	No	No	No	No
No	No	No	No	No	No	No	No	No
No	No	No	No	No	No	No	No	No
No	No	No	Yes	Yes	No	No	No	No
No	No	No	No	No	No	No	No	No
No	No	No	No	No	No	No	No	No
No	No	No	No	No	No	No	No	No
No	No	No	No	No	No	No	No	No

skin_prob	age_cat	income_ca	n_allergic	n_trauma	n_other	n_acne
1	21-35 year	30000-600	0	0	1	0
1	21-35 year	30000-600	1	0	0	0
0	21-35 year	30000-600	0	0	0	0
0	> 35 years	30000-600	0	0	0	0
0	> 35 years	30000-600	0	0	0	0
1	21-35 year	30000-600	0	1	1	1
1	<=20 years	30000-600	0	0	1	1
0	> 35 years	30000-600	0	0	0	0
1	21-35 year	30000-600	0	0	0	1
0	> 35 years	30000-600	0	0	0	0
0	21-35 year	30000-600	0	0	0	0
1	21-35 year	30000-600	1	0	1	1
1	> 35 years	30000-600	1	1	1	0
0	21-35 year	>60000	0	0	0	0
0	<=20 years	<30000	0	0	0	0
1	> 35 years	<30000	0	1	1	0
1	> 35 years	30000-600	1	1	0	0
0	21-35 year	30000-600	0	0	0	0
0	> 35 years	30000-600	0	0	0	0
1	> 35 years	30000-600	1	1	1	0
0	> 35 years	>60000	0	0	0	0
0	<=20 years	30000-600	0	0	0	0
0	> 35 years	30000-600	0	0	0	0
0	> 35 years	<30000	0	0	0	0
1	21-35 year	<30000	1	0	0	0
0	> 35 years	30000-600	0	0	0	0
1	<=20 years	30000-600	0	0	0	1
1	<=20 years	>60000	1	1	1	1
1	<=20 years	>60000	1	0	0	1
0	<=20 years	30000-600	0	0	0	0
1	21-35 year	<30000	0	1	0	0
1	> 35 years	30000-600	0	1	0	0
0	21-35 year	30000-600	0	0	0	0
1	<=20 years	30000-600	0	0	0	1
0	21-35 year	>60000	0	0	0	0
1	21-35 year	30000-600	0	0	0	1
1	<=20 years	>60000	1	0	1	0
1	21-35 year	30000-600	0	1	0	0
1	<=20 years	30000-600	1	0	0	1
0	<=20 years	<30000	0	0	0	0
0	21-35 year	<30000	0	0	0	0
1	21-35 year	30000-600	1	0	0	1
0	21-35 year	30000-600	0	0	0	0
0	<=20 years	30000-600	0	0	0	0
1	<=20 years	30000-600	0	0	0	1
1	21-35 year	>60000	1	0	1	0
0	21-35 year	30000-600	0	0	0	0
1	> 35 years	30000-600	1	0	0	0
0	21-35 year	>60000	0	0	0	0
1	> 35 years	30000-600	1	0	0	0
0	21-35 year	30000-600	0	0	0	0

0	<=20 years	>60000	0	0	0	0
1	<=20 years	<30000	0	1	0	0
0	21-35 years	<30000	0	0	0	0
1	21-35 years	30000-600	0	0	1	1
1	<=20 years	30000-600	1	1	0	1
0	21-35 years	<30000	0	0	0	0
0	<=20 years	>60000	0	0	0	0
0	<=20 years	30000-600	0	0	0	0
1	<=20 years	<30000	1	1	1	0
0	<=20 years	30000-600	0	0	0	0
1	<=20 years	30000-600	1	0	0	1
1	<=20 years	30000-600	1	1	0	1
0	21-35 years	>60000	0	0	0	0
0	<=20 years	30000-600	0	0	0	0
0	<=20 years	<30000	0	0	0	0
0	21-35 years	<30000	0	0	0	0
1	<=20 years	30000-600	0	0	1	0
1	<=20 years	30000-600	1	1	0	0
0	<=20 years	30000-600	0	0	0	0
1	> 35 years	>60000	1	0	0	0
1	<=20 years	<30000	1	0	0	0
1	21-35 years	30000-600	0	0	0	1
0	<=20 years	>60000	0	0	0	0
1	> 35 years	<30000	1	0	0	0
0	<=20 years	<30000	0	0	0	0
0	21-35 years	>60000	0	0	0	0
1	<=20 years	>60000	1	0	0	0
0	21-35 years	<30000	0	0	0	0
1	21-35 years	30000-600	0	1	0	1
1	<=20 years	30000-600	1	0	0	0
1	<=20 years	>60000	0	1	0	0
0	<=20 years	30000-600	0	0	0	0
0	21-35 years	30000-600	0	0	1	0
0	<=20 years	<30000	0	0	0	0
1	21-35 years	>60000	1	0	0	0
1	21-35 years	>60000	1	1	0	1
1	> 35 years	>60000	0	1	0	0
0	<=20 years	>60000	0	0	0	0
0	21-35 years	>60000	0	0	0	0
0	<=20 years	30000-600	0	0	0	0
0	21-35 years	<30000	0	0	0	0
0	<=20 years	>60000	0	0	0	0
0	21-35 years	>60000	0	0	0	0
0	<=20 years	>60000	0	0	0	0
0	<=20 years	<30000	0	0	0	0
0	21-35 years	30000-600	0	0	0	0
1	<=20 years	30000-600	1	1	0	1
0	<=20 years	30000-600	0	0	0	0
0	<=20 years	>60000	0	0	0	0
0	21-35 years	>60000	0	0	0	0
1	21-35 years	>60000	0	1	0	0
0	21-35 years	<30000	0	0	0	0

0	<=20 years	<30000	0	0	0	0
0	<=20 years	<30000	0	0	0	0
0	> 35 years	30000-600	0	0	0	0
1	21-35 year	30000-600	1	0	0	1
1	<=20 years	30000-600	0	1	0	0
1	<=20 years	30000-600	0	1	0	0
0	<=20 years	30000-600	0	0	1	0
1	21-35 year	>60000	1	1	1	0
0	21-35 year	<30000	0	0	0	0
0	21-35 year	30000-600	0	0	0	0
1	<=20 years	30000-600	0	1	0	0
0	21-35 year	>60000	0	0	0	0
1	21-35 year	<30000	1	0	0	0
0	> 35 years	30000-600	0	0	0	0
1	<=20 years	30000-600	0	1	1	0
1	21-35 year	>60000	1	0	1	1
0	21-35 year	30000-600	0	0	0	0
1	<=20 years	30000-600	1	0	0	0
0	<=20 years	>60000	0	0	0	0
0	21-35 year	30000-600	0	0	0	0
0	21-35 year	30000-600	0	0	0	0
1	<=20 years	<30000	0	0	0	1
1	21-35 year	>60000	0	1	0	0
0	<=20 years	>60000	0	0	0	0
0	> 35 years	>60000	0	0	0	0
0	21-35 year	30000-600	0	0	0	0
1	<=20 years	30000-600	0	0	1	1
0	<=20 years	30000-600	0	0	0	0
1	<=20 years	>60000	0	1	0	1
0	<=20 years	30000-600	0	0	0	0
0	21-35 year	>60000	0	0	0	0
0	<=20 years	>60000	0	0	0	0
1	21-35 year	30000-600	1	1	1	0
0	21-35 year	30000-600	0	0	0	0
0	21-35 year	<30000	0	0	0	0
0	<=20 years	>60000	0	0	0	0
0	<=20 years	30000-600	0	0	0	0
0	<=20 years	>60000	0	0	0	0
0	21-35 year	30000-600	0	0	0	0
1	21-35 year	30000-600	1	0	0	0
0	21-35 year	<30000	0	0	0	0
1	21-35 year	30000-600	0	1	0	0
0	21-35 year	30000-600	0	0	0	0
0	21-35 year	30000-600	0	0	0	0
0	> 35 years	30000-600	0	0	0	0
0	> 35 years	30000-600	0	0	0	0
0	21-35 year	>60000	0	0	0	0
0	21-35 year	>60000	0	0	0	0
0	> 35 years	30000-600	0	0	0	0
0	<=20 years	<30000	0	0	0	0
0	21-35 year	30000-600	0	0	0	0
1	<=20 years	30000-600	0	0	0	1

0	21-35 year	30000-600	0	0	0	0
0	<=20 years	>60000	0	0	0	0
0	21-35 year	30000-600	0	0	0	0
0	21-35 year	30000-600	0	0	1	0
0	<=20 years	>60000	0	0	0	0
0	<=20 years	30000-600	0	0	0	0
1	<=20 years	30000-600	1	1	1	1
0	<=20 years	30000-600	0	0	0	0
1	21-35 year	>60000	0	1	0	0
1	<=20 years	30000-600	0	1	0	0
1	<=20 years	<30000	1	1	1	1
0	21-35 year	>60000	0	0	0	0
0	<=20 years	30000-600	0	0	0	0
0	21-35 year	30000-600	0	0	0	0
1	21-35 year	30000-600	1	1	1	1
0	<=20 years	>60000	0	0	0	0
0	21-35 year	30000-600	0	0	0	0
0	21-35 year	30000-600	0	0	0	0
1	21-35 year	30000-600	0	1	0	0
1	21-35 year	30000-600	0	0	0	1
1	<=20 years	>60000	1	1	1	0
0	<=20 years	>60000	0	0	0	0
1	<=20 years	>60000	0	1	0	0
1	21-35 year	>60000	0	0	0	1
0	<=20 years	30000-600	0	0	0	0
0	21-35 year	<30000	0	0	0	0
1	21-35 year	30000-600	0	0	1	1
0	21-35 year	30000-600	0	0	0	0
1	21-35 year	>60000	0	0	0	1
0	<=20 years	30000-600	0	0	0	0
0	<=20 years	30000-600	0	0	0	0
1	<=20 years	>60000	1	1	0	1
1	21-35 year	30000-600	0	1	0	0
0	21-35 year	30000-600	0	0	1	0
1	<=20 years	>60000	1	1	0	1
0	<=20 years	>60000	0	0	0	0
0	<=20 years	>60000	0	0	0	0
0	21-35 year	>60000	0	0	0	0
1	<=20 years	30000-600	0	1	0	1
0	<=20 years	30000-600	0	0	0	0
0	<=20 years	30000-600	0	0	0	0
1	21-35 year	30000-600	0	0	0	1
0	21-35 year	>60000	0	0	0	0
0	<=20 years	30000-600	0	0	0	0
1	21-35 year	>60000	0	1	0	1
0	<=20 years	30000-600	0	0	0	0
0	> 35 years	>60000	0	0	0	0
0	<=20 years	30000-600	0	0	0	0
0	21-35 year	>60000	0	0	0	0
0	<=20 years	30000-600	0	0	0	0
0	21-35 year	30000-600	0	0	0	0
0	<=20 years	<30000	0	0	0	0

1	<=20 years	30000-600	1	0	1	1
0	<=20 years	30000-600	0	0	0	0
1	> 35 years	30000-600	1	0	1	0
0	> 35 years	30000-600	0	0	0	0
1	> 35 years	<30000	1	0	1	1
1	21-35 year	<30000	1	1	0	1
1	21-35 year	<30000	0	1	0	0
0	<=20 years	30000-600	0	0	0	0
1	21-35 year	30000-600	1	0	0	1
1	> 35 years	<30000	0	1	0	0
0	<=20 years	<30000	0	0	0	0
1	21-35 year	30000-600	1	0	0	0
0	21-35 year	30000-600	0	0	0	0
1	21-35 year	>60000	0	0	0	1
0	21-35 year	30000-600	0	0	0	0
1	21-35 year	<30000	0	1	0	0
0	21-35 year	<30000	0	0	0	0
1	21-35 year	30000-600	0	0	0	1
1	21-35 year	>60000	1	1	0	1
1	21-35 year	>60000	1	0	0	1
0	21-35 year	>60000	0	0	0	0
0	21-35 year	30000-600	0	0	0	0
0	<=20 years	<30000	0	0	0	0
0	21-35 year	30000-600	0	0	0	0
1	21-35 year	>60000	1	1	0	0
0	> 35 years	<30000	0	0	0	0
1	> 35 years	<30000	0	0	1	0
0	> 35 years	>60000	0	0	0	0
1	21-35 year	30000-600	1	0	0	0
1	21-35 year	<30000	1	1	1	1
0	21-35 year	<30000	0	0	0	0
1	21-35 year	<30000	0	0	1	0
0	21-35 year	30000-600	0	0	0	0
1	21-35 year	30000-600	0	1	0	0
0	<=20 years	30000-600	0	0	0	0
1	<=20 years	30000-600	0	0	0	1
0	21-35 year	>60000	0	0	0	0
0	21-35 year	>60000	0	0	0	0
1	21-35 year	<30000	1	1	0	0
1	21-35 year	>60000	0	1	0	0
1	<=20 years	>60000	0	0	0	1
0	<=20 years	30000-600	0	0	0	0
0	<=20 years	30000-600	0	0	0	0
1	<=20 years	>60000	1	1	0	0
1	21-35 year	30000-600	0	1	1	0
0	<=20 years	30000-600	0	0	0	0
1	<=20 years	>60000	0	0	0	1
1	> 35 years	30000-600	1	0	0	0
1	<=20 years	>60000	1	0	1	1
1	21-35 year	30000-600	0	1	0	0
1	21-35 year	>60000	0	1	1	0
0	21-35 year	30000-600	0	0	0	0

0	21-35 years	30000-600	0	0	0	0
0	21-35 years	30000-600	0	0	0	0
1	<=20 years	>60000	1	0	0	0
0	<=20 years	30000-600	0	0	0	0
1	21-35 years	30000-600	1	0	0	1
1	<=20 years	<30000	0	0	0	1
1	> 35 years	>60000	0	1	0	0
0	21-35 years	>60000	0	0	0	0
0	> 35 years	>60000	0	0	0	0
0	<=20 years	30000-600	0	0	0	0
0	21-35 years	30000-600	0	0	0	0
0	21-35 years	30000-600	0	0	0	0
0	> 35 years	30000-600	0	0	0	0
1	> 35 years	30000-600	0	0	1	1
1	> 35 years	30000-600	1	0	1	0
0	> 35 years	30000-600	0	0	0	0
0	> 35 years	<30000	0	0	0	0
1	> 35 years	30000-600	1	1	1	0
1	> 35 years	30000-600	0	1	1	0
1	21-35 years	>60000	0	1	1	0
1	21-35 years	>60000	0	1	0	0
0	21-35 years	30000-600	0	0	0	0
1	21-35 years	>60000	0	0	0	1
0	<=20 years	30000-600	0	0	0	0
0	<=20 years	<30000	0	0	0	0
1	<=20 years	30000-600	1	0	0	0
0	<=20 years	<30000	0	0	0	0
0	<=20 years	30000-600	0	0	0	0
0	21-35 years	<30000	0	0	0	0
0	<=20 years	30000-600	0	0	0	0
1	21-35 years	30000-600	0	1	1	0
1	<=20 years	30000-600	0	0	0	1
0	<=20 years	30000-600	0	0	0	0
0	<=20 years	30000-600	0	0	0	0
0	<=20 years	<30000	0	0	0	0
1	<=20 years	<30000	1	0	0	0
1	21-35 years	30000-600	1	1	0	0
1	21-35 years	<30000	0	1	0	0
0	21-35 years	30000-600	0	0	0	0
0	21-35 years	<30000	0	0	0	0
0	21-35 years	30000-600	0	0	0	0
0	> 35 years	30000-600	0	0	0	0
1	> 35 years	30000-600	0	1	0	0
0	> 35 years	30000-600	0	0	0	0
0	> 35 years	30000-600	0	0	0	0
0	<=20 years	30000-600	0	0	0	0
0	21-35 years	30000-600	0	0	0	0
1	<=20 years	>60000	1	0	1	0
0	<=20 years	>60000	0	0	0	0
0	<=20 years	<30000	0	0	0	0
1	21-35 years	30000-600	0	0	1	1
0	> 35 years	30000-600	0	0	0	0

1	> 35 years	>60000	1	0	0	0
0	> 35 years	<30000	0	0	0	0
0	> 35 years	<30000	0	0	0	0
0	> 35 years	>60000	0	0	0	0
0	21-35 year	<30000	0	0	0	0
0	<=20 years	30000-600	0	0	0	0
1	21-35 year	>60000	0	1	0	0
0	<=20 years	30000-600	0	0	0	0
0	21-35 year	<30000	0	0	0	0
0	<=20 years	>60000	0	0	0	0
0	> 35 years	>60000	0	0	0	0
0	21-35 year	>60000	0	0	0	0
0	<=20 years	>60000	0	0	0	0
0	21-35 year	<30000	0	0	0	0
1	<=20 years	30000-600	1	1	1	1
0	> 35 years	30000-600	0	0	0	0
0	21-35 year	30000-600	0	0	0	0
1	21-35 year	30000-600	0	1	0	0
1	21-35 year	30000-600	1	0	0	1
0	21-35 year	<30000	0	0	0	0
0	21-35 year	30000-600	0	0	0	0
1	<=20 years	30000-600	0	0	0	1
1	21-35 year	30000-600	1	0	1	1
1	<=20 years	30000-600	1	0	1	1
0	<=20 years	<30000	0	0	0	0
0	<=20 years	30000-600	0	0	0	0
1	<=20 years	>60000	0	0	0	1
0	<=20 years	<30000	0	0	0	0
0	<=20 years	<30000	0	0	0	0
0	> 35 years	<30000	0	0	0	0
0	21-35 year	<30000	0	0	0	0
1	> 35 years	30000-600	1	0	0	0
1	<=20 years	>60000	0	0	0	1
0	21-35 year	>60000	0	0	0	0
1	21-35 year	30000-600	1	0	0	0
0	21-35 year	30000-600	0	0	0	0
0	<=20 years	30000-600	0	0	0	0
0	21-35 year	30000-600	0	0	0	0
0	<=20 years	30000-600	0	0	1	0
0	21-35 year	>60000	0	0	0	0
0	<=20 years	30000-600	0	0	0	0
1	<=20 years	>60000	1	0	0	1
1	<=20 years	30000-600	0	1	1	0
1	21-35 year	<30000	1	1	1	1
0	21-35 year	>60000	0	0	0	0
1	21-35 year	30000-600	1	0	1	1
1	21-35 year	30000-600	1	0	0	0
1	21-35 year	>60000	1	0	0	0
0	21-35 year	>60000	0	0	0	0
1	21-35 year	30000-600	0	1	0	0
1	21-35 year	>60000	0	1	0	0
0	21-35 year	30000-600	0	0	0	0

0	21-35 year	<30000	0	0	0	0
0	<=20 years	>60000	0	0	0	0
0	21-35 year	30000-600	0	0	0	0
1	21-35 year	30000-600	1	0	1	1
0	21-35 year	30000-600	0	0	0	0
1	21-35 year	30000-600	1	1	1	1
1	21-35 year	30000-600	0	1	0	0
1	<=20 years	>60000	1	0	0	0
0	21-35 year	30000-600	0	0	0	0
0	<=20 years	30000-600	0	0	0	0
0	> 35 years	30000-600	0	0	0	0
0	> 35 years	30000-600	0	0	0	0
0	<=20 years	30000-600	0	0	0	0
0	<=20 years	>60000	0	0	0	0
0	21-35 year	30000-600	0	0	0	0
0	21-35 year	<30000	0	0	0	0
0	<=20 years	30000-600	0	0	0	0
0	<=20 years	<30000	0	0	0	0
0	> 35 years	30000-600	0	0	0	0
0	> 35 years	30000-600	0	0	0	0
0	> 35 years	>60000	0	0	0	0
0	> 35 years	>60000	0	0	0	0
1	> 35 years	<30000	0	1	1	0
1	21-35 year	<30000	0	1	0	0
0	> 35 years	<30000	0	0	0	0
1	> 35 years	>60000	0	1	0	0
1	21-35 year	30000-600	1	1	1	0
0	21-35 year	>60000	0	0	0	0
0	21-35 year	<30000	0	0	0	0
0	21-35 year	>60000	0	0	0	0
0	21-35 year	>60000	0	0	0	0
0	21-35 year	30000-600	0	0	0	0
1	21-35 year	30000-600	1	0	0	1
0	21-35 year	30000-600	0	0	0	0
0	21-35 year	<30000	0	0	0	0
0	<=20 years	<30000	0	0	0	0
1	21-35 year	>60000	0	1	0	0
0	21-35 year	30000-600	0	0	0	0
0	> 35 years	30000-600	0	0	0	0
1	21-35 year	30000-600	1	1	0	1
1	<=20 years	<30000	1	0	0	1
1	21-35 year	30000-600	0	1	0	0
0	21-35 year	>60000	0	0	0	0
1	21-35 year	<30000	0	1	0	0
0	21-35 year	30000-600	0	0	0	0
0	21-35 year	30000-600	0	0	0	0
1	21-35 year	30000-600	1	0	0	0
1	21-35 year	>60000	0	1	0	0
0	21-35 year	30000-600	0	0	0	0
0	> 35 years	30000-600	0	0	0	0
0	21-35 year	30000-600	0	0	0	0
0	21-35 year	30000-600	0	0	0	0

0	21-35 year:	30000-600	0	0	0	0
1	21-35 year:	30000-600	0	1	0	0
0	> 35 years	>60000	0	0	0	0
1	21-35 year:	>60000	1	0	0	0
0	21-35 year:	>60000	0	0	0	0
0	21-35 year:	30000-600	0	0	0	0
1	21-35 year:	30000-600	1	0	1	0
1	21-35 year:	<30000	1	0	0	0
1	21-35 year:	30000-600	0	1	0	0
1	21-35 year:	30000-600	0	1	0	0
0	<=20 years	30000-600	0	0	0	0
0	21-35 year:	>60000	0	0	1	0
0	21-35 year:	30000-600	0	0	0	0
0	21-35 year:	<30000	0	0	0	0
0	21-35 year:	>60000	0	0	0	0
1	21-35 year:	30000-600	1	0	0	0
0	<=20 years	30000-600	0	0	0	0
1	21-35 year:	>60000	0	1	0	1
0	21-35 year:	30000-600	0	0	0	0
0	<=20 years	<30000	0	0	0	0
1	21-35 year:	30000-600	0	1	0	0
1	<=20 years	30000-600	0	0	0	1
0	21-35 year:	>60000	0	0	0	0
0	21-35 year:	30000-600	0	0	0	0
1	21-35 year:	>60000	1	0	0	0
0	21-35 year:	<30000	0	0	0	0
0	<=20 years	<30000	0	0	0	0
1	21-35 year:	>60000	0	1	0	1
1	21-35 year:	30000-600	0	0	0	1
0	<=20 years	30000-600	0	0	0	0
0	21-35 year:	30000-600	0	0	0	0
0	21-35 year:	>60000	0	0	0	0
1	21-35 year:	<30000	1	0	0	0
0	21-35 year:	<30000	0	0	0	0
1	21-35 year:	30000-600	1	0	0	1
0	21-35 year:	<30000	0	0	0	0
1	21-35 year:	30000-600	1	1	0	0
0	> 35 years	<30000	0	0	0	0
1	21-35 year:	30000-600	1	0	0	0
0	21-35 year:	30000-600	0	0	0	0
1	> 35 years	30000-600	1	0	0	0
0	21-35 year:	30000-600	0	0	0	0
0	> 35 years	>60000	0	0	0	0
1	<=20 years	30000-600	0	1	0	1
0	21-35 year:	30000-600	0	0	0	0
0	21-35 year:	30000-600	0	0	1	0
1	<=20 years	30000-600	0	0	1	0
0	> 35 years	>60000	0	0	0	0
1	21-35 year:	30000-600	0	1	0	1
1	21-35 year:	30000-600	0	1	1	0
0	<=20 years	>60000	0	0	0	0
0	<=20 years	>60000	0	0	0	0

0	21-35 years	>60000	0	0	0	0
0	21-35 years	>60000	0	0	0	0
1	> 35 years	30000-6000	0	1	1	0
1	> 35 years	30000-6000	0	1	0	1
0	21-35 years	<30000	0	0	0	0
0	<=20 years	>60000	0	0	0	0
0	<=20 years	<30000	0	0	0	0
0	<=20 years	<30000	0	0	0	0
0	> 35 years	<30000	0	0	0	0
0	21-35 years	<30000	0	0	0	0
0	21-35 years	<30000	0	0	0	0
0	21-35 years	<30000	0	0	0	0
1	21-35 years	<30000	0	0	1	0
0	<=20 years	30000-6000	0	0	0	0
1	<=20 years	30000-6000	0	1	0	1
0	21-35 years	<30000	0	0	0	0
1	> 35 years	<30000	0	1	0	0
0	<=20 years	<30000	0	0	0	0
0	21-35 years	<30000	0	0	0	0
0	21-35 years	<30000	0	0	0	0
0	> 35 years	<30000	0	0	0	0
0	21-35 years	<30000	0	0	0	0
0	<=20 years	<30000	0	0	0	0
0	21-35 years	<30000	0	0	0	0
0	21-35 years	<30000	0	0	0	0
1	<=20 years	30000-6000	1	0	0	0
1	<=20 years	30000-6000	1	0	0	1
1	<=20 years	<30000	1	0	1	0
1	21-35 years	30000-6000	1	1	0	0
0	21-35 years	30000-6000	0	0	0	0
0	> 35 years	30000-6000	0	0	0	0
1	> 35 years	30000-6000	1	0	0	0
1	21-35 years	<30000	0	1	1	0
1	21-35 years	<30000	0	1	1	0
1	21-35 years	<30000	1	1	1	0
0	<=20 years	<30000	0	0	0	0
1	21-35 years	30000-6000	1	0	0	0
1	21-35 years	<30000	0	1	0	0
0	21-35 years	<30000	0	0	0	0
1	> 35 years	<30000	1	0	1	0
0	21-35 years	>60000	0	0	0	0
0	> 35 years	>60000	0	0	0	0
0	> 35 years	>60000	0	0	0	0
0	> 35 years	30000-6000	0	0	0	0
0	> 35 years	30000-6000	0	0	0	0
0	> 35 years	>60000	0	0	0	0
0	21-35 years	30000-6000	0	0	0	0
0	> 35 years	>60000	0	0	0	0
0	> 35 years	<30000	0	0	0	0
1	> 35 years	<30000	0	1	0	0
1	<=20 years	30000-6000	0	1	0	1
1	21-35 years	30000-6000	0	1	0	0

0	> 35 years	30000-600	0	0	0	0
0	21-35 year	>60000	0	0	0	0
0	21-35 year	>60000	0	0	0	0
0	21-35 year	>60000	0	0	0	0
0	21-35 year	>60000	0	0	0	0
0	> 35 years	<30000	0	0	0	0
1	<=20 years	>60000	0	0	0	1
1	21-35 year	30000-600	1	0	0	0
1	> 35 years	>60000	0	1	0	0
0	21-35 year	>60000	0	0	0	0
0	> 35 years	30000-600	0	0	0	0
1	21-35 year	<30000	0	1	0	0
1	21-35 year	<30000	1	0	0	0
0	> 35 years	30000-600	0	0	0	0
0	> 35 years	30000-600	0	0	0	0
0	21-35 year	<30000	0	0	0	0
0	> 35 years	>60000	0	0	0	0
0	> 35 years	>60000	0	0	0	0
0	> 35 years	>60000	0	0	0	0
0	21-35 year	>60000	0	0	0	0
1	> 35 years	>60000	1	0	1	1
0	> 35 years	>60000	0	0	0	0
0	> 35 years	<30000	0	0	0	0
0	> 35 years	30000-600	0	0	0	0
0	> 35 years	30000-600	0	0	0	0
1	<=20 years	>60000	0	0	0	1
0	> 35 years	>60000	0	0	0	0
1	> 35 years	<30000	1	0	1	0
1	> 35 years	<30000	1	1	0	0
1	> 35 years	30000-600	0	1	1	0
1	> 35 years	<30000	0	1	0	0
1	21-35 year	>60000	0	1	0	0
0	> 35 years	>60000	0	0	0	0
0	> 35 years	>60000	0	0	0	0
1	> 35 years	<30000	1	0	0	0
1	21-35 year	>60000	1	0	0	0
1	> 35 years	<30000	1	0	1	0
1	<=20 years	>60000	1	0	0	0
0	> 35 years	<30000	0	0	0	0
1	> 35 years	<30000	1	1	0	0
1	> 35 years	30000-600	0	1	0	0
1	> 35 years	<30000	1	0	0	0
1	> 35 years	<30000	0	1	0	0
0	> 35 years	<30000	0	0	0	0
0	> 35 years	<30000	0	0	0	0
0	> 35 years	30000-600	0	0	0	0
0	21-35 year	30000-600	0	0	0	0
0	> 35 years	>60000	0	0	0	0
0	> 35 years	30000-600	0	0	0	0
0	> 35 years	<30000	0	0	0	0
0	> 35 years	>60000	0	0	0	0
0	> 35 years	>60000	0	0	0	0

0	21-35 year	<30000	0	0	0	0
1	21-35 year	30000-600	0	1	0	0
1	> 35 years	30000-600	1	0	0	0
0	> 35 years	30000-600	0	0	0	0
0	> 35 years	<30000	0	0	0	0
0	> 35 years	>60000	0	0	0	0
0	> 35 years	>60000	0	0	0	0
0	> 35 years	>60000	0	0	0	0
0	21-35 year	<30000	0	0	0	0
1	> 35 years	30000-600	0	1	1	0
0	> 35 years	30000-600	0	0	0	0
1	21-35 year	30000-600	0	0	0	1
0	21-35 year	30000-600	0	0	0	0
1	21-35 year	>60000	1	0	1	0
1	21-35 year	30000-600	0	0	0	1
1	21-35 year	>60000	1	1	1	1
1	21-35 year	30000-600	1	0	0	0
0	21-35 year	<30000	0	0	0	0
0	21-35 year	<30000	0	0	0	0
1	21-35 year	30000-600	0	1	1	0
1	21-35 year	<30000	0	1	0	1
1	<=20 years	30000-600	1	1	0	0
1	21-35 year	<30000	0	0	1	1
0	21-35 year	<30000	0	0	0	0
0	<=20 years	30000-600	0	0	0	0
1	21-35 year	30000-600	0	0	1	0
0	21-35 year	<30000	0	0	0	0
1	<=20 years	30000-600	1	0	0	0
0	21-35 year	30000-600	0	0	0	0
0	21-35 year	30000-600	0	0	0	0
1	<=20 years	>60000	0	1	0	0
1	<=20 years	30000-600	0	1	0	0
1	21-35 year	>60000	0	0	1	0
0	<=20 years	30000-600	0	0	0	0
1	21-35 year	>60000	0	0	1	0
1	<=20 years	>60000	0	1	0	0
0	<=20 years	30000-600	0	0	0	0
0	<=20 years	30000-600	0	0	0	0
0	> 35 years	>60000	0	0	0	0
0	> 35 years	>60000	0	0	0	0
0	> 35 years	>60000	0	0	0	0
0	> 35 years	>60000	0	0	0	0
0	> 35 years	>60000	0	0	0	0
0	> 35 years	>60000	0	0	0	0
0	> 35 years	>60000	0	0	0	0
0	> 35 years	>60000	0	0	0	0
1	> 35 years	<30000	0	0	1	0
1	> 35 years	<30000	0	1	0	0
0	> 35 years	<30000	0	0	0	0
1	> 35 years	30000-600	1	1	0	0
1	> 35 years	<30000	0	0	1	0
1	> 35 years	<30000	1	1	0	0

0	> 35 years	<30000	0	0	0	0
0	> 35 years	<30000	0	0	0	0
1	<=20 years	<30000	0	0	1	0
0	> 35 years	<30000	0	0	0	0
1	> 35 years	<30000	0	0	1	0
1	> 35 years	<30000	0	1	0	0
1	> 35 years	30000-600	0	0	1	0
0	> 35 years	30000-600	0	0	0	0
0	> 35 years	<30000	0	0	0	0
0	> 35 years	30000-600	0	0	0	0
1	> 35 years	<30000	0	0	1	0
0	> 35 years	30000-600	0	0	0	0
0	> 35 years	<30000	0	0	0	0
0	21-35 years	<30000	0	0	0	0
0	21-35 years	<30000	0	0	0	0
0	21-35 years	30000-600	0	0	0	0
1	> 35 years	<30000	1	0	0	0
1	> 35 years	30000-600	1	0	0	0
1	21-35 years	<30000	1	1	0	0
1	21-35 years	30000-600	1	0	0	0
0	<=20 years	30000-600	0	0	0	0
0	<=20 years	30000-600	0	0	0	0
1	<=20 years	>60000	0	1	1	1
0	21-35 years	30000-600	0	0	0	0
0	> 35 years	30000-600	0	0	0	0
1	> 35 years	30000-600	0	1	0	0
0	> 35 years	30000-600	0	0	0	0
0	<=20 years	>60000	0	0	0	0
0	> 35 years	30000-600	0	0	0	0
1	21-35 years	30000-600	1	1	0	0
0	> 35 years	30000-600	0	0	0	0
0	21-35 years	>60000	0	0	0	0
1	21-35 years	30000-600	0	1	1	0
0	21-35 years	30000-600	0	0	0	0
0	> 35 years	30000-600	0	0	0	0
1	> 35 years	30000-600	0	1	0	0
0	> 35 years	30000-600	0	0	0	0
1	21-35 years	30000-600	1	1	0	0
0	> 35 years	30000-600	0	0	0	0
0	21-35 years	30000-600	0	0	0	0
0	21-35 years	30000-600	0	0	0	0
1	> 35 years	30000-600	0	1	0	1
0	> 35 years	30000-600	0	0	0	0
1	<=20 years	30000-600	0	1	0	0
1	<=20 years	30000-600	0	1	0	0
0	21-35 years	>60000	0	0	0	0
1	<=20 years	30000-600	0	0	0	1
0	> 35 years	<30000	0	0	0	0
0	21-35 years	>60000	0	0	0	0
0	21-35 years	30000-600	0	0	0	0
1	21-35 years	>60000	1	1	0	1
1	21-35 years	30000-600	1	0	0	0

0	21-35 year	<30000	0	0	0	0
1	21-35 year	<30000	0	1	0	0
0	21-35 year	30000-600	0	0	0	0
1	21-35 year	30000-600	1	0	0	0
1	21-35 year	30000-600	0	1	0	0
0	<=20 years	30000-600	0	0	0	0
1	> 35 years	>60000	0	1	0	0
0	> 35 years	30000-600	0	0	0	0
1	21-35 year	30000-600	0	1	1	1
1	<=20 years	>60000	1	0	0	1
1	<=20 years	30000-600	0	0	0	1
1	<=20 years	30000-600	0	0	0	1
0	21-35 year	30000-600	0	0	0	0
0	21-35 year	30000-600	0	0	0	0
0	<=20 years	<30000	0	0	0	0
0	21-35 year	30000-600	0	0	0	0
0	21-35 year	>60000	0	0	0	0
1	21-35 year	30000-600	1	1	1	1
0	> 35 years	30000-600	0	0	0	0
0	> 35 years	30000-600	0	0	0	0
0	21-35 year	30000-600	0	0	0	0
1	<=20 years	<30000	0	0	1	1
1	21-35 year	30000-600	1	0	1	0
0	21-35 year	30000-600	0	0	0	0
0	21-35 year	30000-600	0	0	0	0
1	<=20 years	<30000	0	0	0	1
1	21-35 year	>60000	0	0	0	1
0	21-35 year	>60000	0	0	0	0
0	21-35 year	30000-600	0	0	0	0
0	21-35 year	>60000	0	0	0	0
1	21-35 year	<30000	0	0	0	1
1	> 35 years	>60000	1	0	1	1
1	> 35 years	>60000	0	1	1	1
1	<=20 years	>60000	1	0	0	0
1	> 35 years	>60000	1	0	0	0
1	21-35 year	30000-600	0	1	1	1
0	21-35 year	30000-600	0	0	0	0
1	21-35 year	<30000	0	1	0	1
1	<=20 years	>60000	0	0	0	1
0	21-35 year	30000-600	0	0	0	0
0	21-35 year	30000-600	0	0	0	0
0	21-35 year	>60000	0	0	0	0
0	<=20 years	>60000	0	0	0	0
1	<=20 years	>60000	1	0	0	0
0	> 35 years	>60000	0	0	0	0
0	21-35 year	30000-600	0	0	0	0
0	21-35 year	30000-600	0	0	0	0
0	<=20 years	>60000	0	0	0	0
0	21-35 year	30000-600	0	0	0	0
1	21-35 year	30000-600	1	0	1	0
1	21-35 year	<30000	1	1	1	1
1	<=20 years	>60000	0	1	1	0

0	> 35 years	>60000	0	0	0	0
0	<=20 years	>60000	0	0	0	0
0	21-35 years	>60000	0	0	0	0
0	21-35 years	30000-6000	0	0	0	0
0	21-35 years	30000-6000	0	0	0	0
0	21-35 years	30000-6000	0	0	0	0
0	21-35 years	>60000	0	0	0	0
0	> 35 years	30000-6000	0	0	0	0
0	> 35 years	30000-6000	0	0	0	0
0	21-35 years	30000-6000	0	0	0	0
1	21-35 years	30000-6000	0	0	0	1
0	21-35 years	30000-6000	0	0	0	0
0	21-35 years	<30000	0	0	0	0
0	21-35 years	<30000	0	0	0	0
0	> 35 years	<30000	0	0	0	0
0	21-35 years	30000-6000	0	0	0	0
0	21-35 years	30000-6000	0	0	0	0
1	<=20 years	<30000	1	0	0	0
1	21-35 years	30000-6000	0	1	1	1
1	21-35 years	<30000	1	0	0	1
1	21-35 years	>60000	1	0	1	0
1	21-35 years	>60000	0	1	0	0
0	> 35 years	>60000	0	0	0	0
0	> 35 years	>60000	0	0	0	0
0	> 35 years	>60000	0	0	0	0
0	> 35 years	>60000	0	0	0	0
0	> 35 years	>60000	0	0	0	0
1	21-35 years	30000-6000	1	1	0	0
1	21-35 years	<30000	1	0	0	0
0	> 35 years	>60000	0	0	0	0
0	> 35 years	>60000	0	0	0	0
0	> 35 years	>60000	0	0	0	0
0	> 35 years	>60000	0	0	0	0
0	21-35 years	>60000	0	0	0	0
0	> 35 years	>60000	0	0	0	0
0	21-35 years	>60000	0	0	0	0
0	> 35 years	>60000	0	0	0	0
0	> 35 years	>60000	0	0	0	0
1	21-35 years	30000-6000	0	1	0	0
0	<=20 years	>60000	0	0	0	0
0	21-35 years	30000-6000	0	0	0	0
0	21-35 years	<30000	0	0	0	0
0	21-35 years	<30000	0	0	0	0
0	> 35 years	<30000	0	0	0	0
1	<=20 years	<30000	1	0	0	0
0	21-35 years	<30000	0	0	0	0
0	> 35 years	<30000	0	0	0	0
0	> 35 years	<30000	0	0	0	0
1	> 35 years	<30000	0	1	0	0
1	> 35 years	<30000	0	1	0	0
0	21-35 years	>60000	0	0	0	0
0	21-35 years	30000-6000	0	0	0	0

1	> 35 years	<30000	0	1	0	0
0	> 35 years	<30000	0	0	0	0
1	> 35 years	<30000	0	1	0	0
1	> 35 years	<30000	0	1	0	0
1	> 35 years	<30000	1	0	0	0
1	> 35 years	<30000	0	1	1	0
1	> 35 years	<30000	0	0	1	0
1	> 35 years	<30000	1	0	0	0
1	> 35 years	<30000	0	0	1	0
1	> 35 years	<30000	1	0	0	0
1	> 35 years	<30000	1	0	0	0
1	> 35 years	<30000	0	0	1	0
1	> 35 years	<30000	0	1	1	0
1	> 35 years	30000-600	0	1	0	0
1	> 35 years	<30000	0	1	1	0
0	> 35 years	<30000	0	0	0	0
1	> 35 years	<30000	0	1	0	0
1	> 35 years	<30000	0	0	1	0
0	> 35 years	30000-600	0	0	0	0
1	> 35 years	30000-600	0	1	1	0
0	21-35 year	30000-600	0	0	0	0
0	> 35 years	<30000	0	0	0	0
0	> 35 years	30000-600	0	0	0	0
0	<=20 year	30000-600	0	0	0	0
