# Supplementary material for: Prevalence of dermatological manifestations due to face mask use and its associated factors during COVID-19 among the general population of Bangladesh: A nationwide cross-sectional survey
Source: PLoS One. 2022 Jun 13;17(6):e0269922. doi: 10.1371/journal.pone.0269922 (PMC9191724; doi:10.1371/journal.pone.0269922)
Supplement: Attachment — Submitted filename: Response to Reviewers.docx [file pone.0269922.s002.docx]

**Rebuttal Letter**

Date: 18 January 2022

Dear Editor,

We thank the editor and the two reviewers for their valuable comments on our manuscript. Below is our response to each point raised by the academic editor and reviewers. We hope that we satisfyingly addressed them and the manuscript will be now suited for publication in your journal.

Sincerely,

Dr. Rifat Ara

Research Physician

Infectious Disease Division

Icddr,b, Dhaka, Bangladesh

On behalf of all authors,

**Response to Reviewers**

ACADEMIC EDITOR:

Title: Please change from: its’ To: its

**Author’s response:** Changed from: its’ to its, as per the suggestion.

Title: Please write the title in a Sentence form. Look for titles of published articles in PLOS ONE.

**Author’s response:** Title has been changed to a Sentence form following published articles in PLOS ONE.

Sampling: Please describe the ‘fixed-step sampling technique’ and report what type of sampling method it is.

**Author’s response:** Our sampling technique is random route sampling. In lines 98-99, sampling technique has been re-written to make it more understandable.

Results: Please avoid repetition of data both in text and tables such as Table 1 and Text (lines 123-145)..

**Author’s response:** Repeated information has been deleted (lines 126-137 and also in the table). To avoid repetition of the information, demographic data along with co-morbidity history have been included in the Table 1. Information related to pattern of facemask use has been given only in the writing part.

Reporting of P values: Table 2 includes p values reported in in 3, 4 and 5 digits. Please report exact p-values for all values greater than or equal to 0.001. P-values less than 0.001 may be expressed as p < 0.001, or as exponentials in studies of genetic associations. For more details, please refer to statistical reporting for PLOS ONE available at <https://journals.plos.org/plosone/s/submission-guidelines>.

**Author’s response:** According to the statistical reporting guideline of PLOS ONE, changes have been made both in tables and writing in case of reporting the P-values. Exact p-values have been reported for all values greater than or equal to 0.001 and P-values less than 0.001 have been expressed as p < 0.001.

Please report how was obesity defined and determined.

**Author’s response:** All co-morbidities including obesity was self-reported and only those were included who were confirmed by physician in the past. This information has been added in the methodology (line 89-91).

Please report strengths and limitations of the study.

**Author’s response:** Strength and limitation of our study have been reported in the discussion (lines 259-262)

Reviewers' comments:

Reviewer's Responses to Questions

Comments to the Author

1. Is the manuscript technically sound, and do the data support the conclusions?

The manuscript must describe a technically sound piece of scientific research with data that supports the conclusions. Experiments must have been conducted rigorously, with appropriate controls, replication, and sample sizes. The conclusions must be drawn appropriately based on the data presented.

Reviewer #1: Yes

Reviewer #2: Yes

**Author’s response:** Thank you.

2. Has the statistical analysis been performed appropriately and rigorously?

Reviewer #1: Yes

Reviewer #2: Yes

**Author’s response:** Thank you.

3. Have the authors made all data underlying the findings in their manuscript fully available?

The PLOS Data policy requires authors to make all data underlying the findings described in their manuscript fully available without restriction, with rare exception (please refer to the Data Availability Statement in the manuscript PDF file). The data should be provided as part of the manuscript or its supporting information, or deposited to a public repository. For example, in addition to summary statistics, the data points behind means, medians and variance measures should be available. If there are restrictions on publicly sharing data—e.g. participant privacy or use of data from a third party—those must be specified.

Reviewer #1: Yes

Reviewer #2: Yes

**Author’s response:** Thank you.

4. Is the manuscript presented in an intelligible fashion and written in standard English?

PLOS ONE does not copyedit accepted manuscripts, so the language in submitted articles must be clear, correct, and unambiguous. Any typographical or grammatical errors should be corrected at revision, so please note any specific errors here.

Reviewer #1: Yes

Reviewer #2: Yes

**Author’s response:** Thank you.

5. Review Comments to the Author

Please use the space provided to explain your answers to the questions above. You may also include additional comments for the author, including concerns about dual publication, research ethics, or publication ethics. (Please upload your review as an attachment if it exceeds 20,000 characters)

Reviewer #1: The manuscript is technically sound and author has made all data in manuscript fully available. The manuscript is well written and in intelligent manner. However few references are not in Vancouver style, which need to be revised. Reference no 11, 14, 15, 16, 17, 18, 24. The new idea from the study is that the dermatoloical manifestion with face mask are higher and usually this was seen in the cases with wearing of surgical mask and when they are used for longer duration as well as those person who are obese or having any skin problem so for prevention of all these problems there is strong need to take the dermatoloical expert opinion regarding the quality type of face mask in the mass population.

**Author’s response:** Reference no. 11, 14, 15, 16, 17, 18, 24 have been corrected to Vancouver style. From the outcome of our study, it is clear that opinions from dermatology experts regarding the quality of different types of masks for the general population are strongly needed.

Reviewer #2: The article is a survey study questioning the dermatological findings related to the use of face mask.

The article is written in a generally understandable flowing style. However, some minor errors were detected.

I suggest the following corrections.

- In the sentence of 'Surgical mask users reported more complaints than regular masks, and prolonged use caused more skin symptoms.' on line 35, regular mask can be specified more clearly and other expression can be used instead of regular, which may be better if it includes other masks in parentheses.

**Author’s response:** It was mistakenly written. The word “regular masks” has been replaced with “other masks” in the revised file.

- In the survey, it should be clearly stated how the definition of obesity is made. Is it according to the participant's own definition, according to the interviewer, or body mass index? In addition, participants who are obese are not specified in Table 1.

**Author’s response:** All co-morbidities including obesity was self-reported and only those were included who were confirmed by physician in the past. This information has been updated in the methodology (line 89). Information regarding obesity as well as other co-morbidities have been added in Table 1.

- There are 4 types of masks in Table 1, the other mask is not specified.

**Author’s response:** Sponge facemask, Snorkel facemask, Full face mask, these were the other types of masks considered in our study. We have also added this in methodology (line 92).

- I think in line 152, figure 1 is placed in the wrong place.

**Author’s response:** As figure 1 explains the percentages of different dermatological manifestations, so it has been added right after the prevalence rates. This has been re-checked and it is alright.

- In Table 2, which statistical test is used should be indicated as a note under the table.

**Author’s response:** We have used Pearson Chi square statistical test in table 2 and this information has been mentioned as footnote (line 169).

- In Table 2, the p value related to others and age is not specified.

**Author’s response:** Accept our apology, it was a major mistake. The missing P-value has been given in the revised file.

- In Table 2, the p value between Average facemask use per week and acne could be <0.001 instead of 0.0005. Again, it would be better if other 5-digit p values were written as 4-digits.

- p<0.001 might be better than p=0.000 in line 172 and table 3.

**Author’s response:** All the P-values (both in tables and writing) have been corrected according to the PLOS ONE statistical reporting guideline.

6. PLOS authors have the option to publish the peer review history of their article (what does this mean?). If published, this will include your full peer review and any attached files.

If you choose “no”, your identity will remain anonymous but your review may still be made public.

Do you want your identity to be public for this peer review? For information about this choice, including consent withdrawal, please see our Privacy Policy.

Reviewer #1: Yes: Prof Dr Meharunnissa Khaskheli

Reviewer #2: No

**Author’s response:** Thank you very much. Best regards.
