# Supplementary material for: Prevalence of dermatological manifestations due to face mask use and its associated factors during COVID-19 among the general population of Bangladesh: A nationwide cross-sectional survey
Source: PLoS One. 2022 Jun 13;17(6):e0269922. doi: 10.1371/journal.pone.0269922 (PMC9191724; doi:10.1371/journal.pone.0269922)
Supplement: Attachment — Submitted filename: Response to Reviewers.docx [file pone.0269922.s003.docx]

**Rebuttal Letter**

Date: 6 April 2022

Dear Editor,

We thank the academic editor and reviewers for their valuable comments on our manuscript. Below is our response to each point raised by the academic editor and reviewers. We reviewed the reference list and added information in methodology and discussion based on the reviewer’s comments. We hope that we satisfyingly addressed them and that the manuscript will be now suited for publication in your journal.

Sincerely,

Dr. Rifat Ara

Research Physician

Infectious Disease Division

Icddr,b, Dhaka, Bangladesh

On behalf of all authors.

**Response to Reviewers**

Journal Requirements:

Please review your reference list to ensure that it is complete and correct. If you have cited papers that have been retracted, please include the rationale for doing so in the manuscript text, or remove these references and replace them with relevant current references. Any changes to the reference list should be mentioned in the rebuttal letter that accompanies your revised manuscript. If you need to cite a retracted article, indicate the article’s retracted status in the References list and also include a citation and full reference for the retraction notice.

**Author’s response:** Thank you very much. We have checked and reviewed the reference list. No cited articles have been retracted so far. So, we ensure that the reference list is complete and correct.

Additional Editor Comments (if provided):

Please address the issues raised by the reviewer(s).

Reviewers' comments:

Reviewer's Responses to Questions

Comments to the Author

1. If the authors have adequately addressed your comments raised in a previous round of review and you feel that this manuscript is now acceptable for publication, you may indicate that here to bypass the “Comments to the Author” section, enter your conflict of interest statement in the “Confidential to Editor” section, and submit your "Accept" recommendation.

Reviewer #1: All comments have been addressed

Reviewer #2: All comments have been addressed

Reviewer #3: All comments have been addressed

2. Is the manuscript technically sound, and do the data support the conclusions?

The manuscript must describe a technically sound piece of scientific research with data that supports the conclusions. Experiments must have been conducted rigorously, with appropriate controls, replication, and sample sizes. The conclusions must be drawn appropriately based on the data presented.

Reviewer #1: Yes

Reviewer #2: Yes

Reviewer #3: Yes

3. Has the statistical analysis been performed appropriately and rigorously?

Reviewer #1: Yes

Reviewer #2: Yes

Reviewer #3: Yes

4. Have the authors made all data underlying the findings in their manuscript fully available?

The PLOS Data policy requires authors to make all data underlying the findings described in their manuscript fully available without restriction, with rare exception (please refer to the Data Availability Statement in the manuscript PDF file). The data should be provided as part of the manuscript or its supporting information, or deposited to a public repository. For example, in addition to summary statistics, the data points behind means, medians and variance measures should be available. If there are restrictions on publicly sharing data—e.g. participant privacy or use of data from a third party—those must be specified.

Reviewer #1: Yes

Reviewer #2: Yes

Reviewer #3: Yes

5. Is the manuscript presented in an intelligible fashion and written in standard English?

PLOS ONE does not copyedit accepted manuscripts, so the language in submitted articles must be clear, correct, and unambiguous. Any typographical or grammatical errors should be corrected at revision, so please note any specific errors here.

Reviewer #1: Yes

Reviewer #2: Yes

Reviewer #3: Yes

6. Review Comments to the Author

Please use the space provided to explain your answers to the questions above. You may also include additional comments for the author, including concerns about dual publication, research ethics, or publication ethics. (Please upload your review as an attachment if it exceeds 20,000 characters)

Reviewer #1: Manuscript is fulfilling the criteria of Plosone , accepted to for publication. This is good effort best research work by the author for writing the manuscript and fulfilling the criteria of manuscript for the joural. So this acceptableb for the publication.

**Author’s response:** Thank you very much for your appreciation and kind words.

Reviewer #2: (No Response)

Reviewer #3: Recommendations reviewer

I think that there is an important and relevant story in this study and I recommend your publication. The prevalence of dermatological manifestations due to face mask use during covid-19 is a very relevant theme. However minor revision is necessary.

Method

A structured questionnaire was utilized to evaluate fot Facemask-related dermatological. This questionnaire was submittig to evaluate for specialists before to study? Or carried out pilot test?

**Author’s response:** Thank you very much for mentioning such an important point. A pilot test was carried out prior to the main study to validate the questionnaire. We have included this information in line 96.

Discussion

In the line 237: From the opinions about the preference of face mask types, cotton cloth masks were more 238 preferable and comfortable among our study participants. Rather, dermatological problems were 239 profound among the surgical facemask users. This outcome is noteworthy, as this predilection 240 significantly can help in decreasing the demand for surgical masks among general people and 241 should be reserved for the healthcare providers during the COVID-19 pandemic. A A prospective 242 survey was conducted in Thailand, where the effects of facemask use have been compared among 243 the healthcare providers and non-healthcare general citizens (24). This survey showed some 244 similar outcomes along with a higher risk of skin problems among the healthcare workers. This is 245 very obvious, as health care workers (HCW) use facemask more frequently and for a longer 246 duration than the non-HCWs.

The comparison with health professionals and the time of use of masks with population data does not seem to be fair, as several factors must be taken into account, as well as the time of use, which may be longer among some individuals in the general population who works for long periods with masks depending on their work activity, not just health professionals.

**Author’s response:** Thank you for your opinion. We also agree with your point but this comparison was made based on general ideas, as the use of masks was compulsory for healthcare providers during the Covid pandemic. However, it is also true that general people also had to wear face masks for longer duration depending on the types and extent of their work. We have added this point in line 247.

7. PLOS authors have the option to publish the peer review history of their article (what does this mean?). If published, this will include your full peer review and any attached files.

**Author’s response:** Thank you for such an important option. We do not have any objection to publishing the peer review history of our article.

If you choose “no”, your identity will remain anonymous but your review may still be made public.

Do you want your identity to be public for this peer review? For information about this choice, including consent withdrawal, please see our Privacy Policy.

Reviewer #1: Yes: Meharunnissa Khaskheli

Reviewer #2: No

Reviewer #3: Yes: Fernanda Ávila

**Author’s response:** Thank you very much for your valuable comments and opinions.
